# Safety and efficacy of bio-engineered, autologous dermo-epidermal skin grafts in adolescent and adult burn patients: 1-year results of a prospective, randomized, controlled, multicenter phase IIB clinical trial

**DOI:** 10.1016/j.eclinm.2025.103665

**Published:** 2025-11-28

**Authors:** Martin Meuli, Clemens Schiestl, Fabienne Hartmann-Fritsch, Esther Middelkoop, Bong-Sung Kim, Kathrin Neuhaus, Jan A. Plock, Daniel Rittirsch, Cornelis H. van der Vlies, Bruno Azzena, Daniela Marino, Kathi Mujynya, Melinda Farkas, Jenny Bressan, Ernst Reichmann, Sophie Böttcher-Haberzeth

**Affiliations:** a University of Zurich, Zurich, Switzerland; b CUTISS AG, Schlieren, Switzerland; c Department of Surgery, Pediatric Burn Center, Children's Skin Center, University Children's Hospital Zurich, University of Zurich, Zurich, Switzerland; d Children’s Research Center, University Children's Hospital Zurich, University of Zurich, Zurich, Switzerland; e Department of Plastic, Amsterdam UMC, Vrije Universiteit Amsterdam, Reconstructive and Hand Surgery, Amsterdam, the Netherlands; f Amsterdam Movement Sciences (AMS), Tissue Function and Regeneration, Amsterdam UMC, Amsterdam, the Netherlands; g Alliance of Dutch Burn Care (ADBC) and Burn Centre, Red Cross Hospital, Beverwijk, the Netherlands; h Department of Plastic Surgery and Hand Surgery, Burn Center, University Hospital Zurich, Zurich, Switzerland; i Department of Plastic Surgery and Hand Surgery, Cantonal Hospital Aarau, Aarau, Switzerland; j Departments of Trauma and Burn Surgery, Maasstad Hospital, Rotterdam, the Netherlands; k Department of Surgery, Trauma Research Unit, Erasmus MC, University Medical Centre Rotterdam, Rotterdam, the Netherlands; l U.O.C. Grandi Ustionati, Azienda Osepdale Università Padova, Padova, Italy; m Department of Surgery, Tissue Biology Research Unit, University Children’s Hospital Zurich, University of Zurich, Schlieren, Switzerland

**Keywords:** Cultured autologous dermo-epidermal skin graft, Burns, Donor site, Scar, Bioengineering, Regenerative medicine, Clinical trial

## Abstract

**Background:**

For burn surgery, major therapeutic challenges are shortage of autologous donor sites for split-thickness skin, and massive devastating scarring. We investigated the therapeutic role of denovoSkin™, a bio-engineered autologous skin, in a prospective, randomized, controlled phase IIb clinical trial compared to the gold standard the split thickness skin graft (STSG).

**Methods:**

Patients, ≥12 years, with deep partial and/or full-thickness burns requiring surgical wound coverage were enrolled at four sites in Switzerland, the Netherlands, and Italy. Two comparable skin defects (max. 98 cm^2^ each) per patient were intra-patient randomized to denovoSkin™ (experimental) or autologous STSG (control) treatment. Safety assessments were performed in all patients. Primary efficacy endpoint was the ratio of biopsy size to grafted area with take 4 weeks post-grafting. The analyses of the primary efficacy variable were performed on the modified Full Analysis Set (mFAS) and the per protocol set (PPS) with the analysis on the mFAS as the primary analysis. Other efficacy endpoints included wound closure and scar quality. Efficacy and safety follow-ups were conducted up to 12 months post-grafting. The trial started in March 2018 and completed recruitment in July 2022 and is registered at clinicaltrials.gov (NCT03227146).

**Findings:**

21 patients were enrolled between March 26, 2018, and July 26 2022. 13 patients were included in the PPS. There were no significant safety differences between denovoSkin™ and STSG. 164 serious adverse events were observed (81%). Only two (hematoma and partial skin necrosis) could be related to denovoSkin™. They rapidly healed spontaneously. The expansion ratio for denovoSkin™ versus STSG was 7·0 times larger for denovoSkin™ (p < 0·001). In the mFAS the mean expansion ratio for denovoSkin™ was 10·76 (SD 6·03), and for STSG it was 1·70 (SD 0·68). The mean ratio of the two expansion ratios (denovoSkin™/STSG) was 7·41 (SD 4·87). By month 3, experimental and control areas were fully epithelialized. Scar evaluation up to 12 months post-grafting revealed no clinically relevant scarring for denovoSkin™, but mostly hypertrophic scarring for STSG.

**Interpretation:**

DenovoSkin™ is a novel and safe treatment option for deep burns. It lessens the need for conventional grafting and so spares donor sites. In sharp contrast to STSG, denovoSkin™ grafting showed minimal scarring, a finding never evidenced before in a randomized trial.

**Funding:**

This study was financed by Wyss Zurich Translational Center (project “denovoSkin”) and CUTISS AG.

**Research in context**

**Evidence before this study**

The use of cultured epithelial autografts (CEA), that is epidermal layers only, for burn treatment has been known since the 1980s. However, over the past four decades, numerous—mostly retrospective—studies have reported inconsistent graft take rates and poor functional and aesthetical long-term clinical outcomes. Despite this, keratinocyte sheets have demonstrated usefulness as additional autologous coverage over widely expanded autologous skin grafts. The first complex, two-layer autologous skin substitutes, incorporating both epidermal and dermal components, were introduced in the late 1990s. While clinical reports have described their use in patients with massive burns (>80% TBSA), no prospective, randomized clinical trial has been published assessing safety, efficacy, and long-term outcomes. A recent systematic review of clinical trials with cell-based therapies for thermal burn wounds by Yassaghi et al. (2024) as well as a review focusing on the challenges during clinical translation of bioengineered skin tissues by Dearman et al. (2021) both confirmed the lack of phase I or phase II clinical trials evaluating advanced dermo-epidermal skin substitutes such as the one investigated here. Thus, our work fills a critical gap regarding evidence-based data generated by innovative bioengineering approaches for severe burns.

**Added value of this study**

To the best of our knowledge, this is the first prospective, intra-patient randomized, controlled, international multicenter trial assessing the benefits of a bio-engineered complex autologous skin with a minimum of 12-month follow-up in comparison to the current gold standard which is meshed split thickness skin grafts (STSG). This trial provides robust clinical evidence for the feasibility, safety, and effectiveness of applying a novel laboratory grown skin substitute in patients with severe burns. The two most striking pieces of evidence, never reported on before in a formal trial, are the very high *expansion rate* from biopsy to cultured graft (expansion options for conventional grafts are small-scale) and the very expeditious graft maturation without *scarring* (conventional grafts, such as STSG, notoriously scar).

**Implications of all the available evidence**

The ground breaking advent of isolating and then culturing keratinocytes to form clinically applicable confluent epidermis-like layers (1975), the introduction of more complex dermo-epidermal skin substitutes for clinical use (1990s), and then, starting in 2000, our own development of denovoSkin™, a bilayer engineered collagen hydrogel-based skin graft composed of autologous keratinocytes and fibroblasts tested in phase I, II, and III (ongoing) clinical trials with favorable preliminary results opens a new prospect: Grafting full thickness skin defects of whatever origin and size with a laboratory grown autologous basically non-scarring skin substitute with theoretically unlimited supply might have a huge impact on clinical practice. The evidence generated by the present study nurtures the hypothesis that the use of bio-engineered skin maturing into a close to natural skin might become a new gold standard for skin replacement. The preliminary experiences from treating massively burned patients suggest that denovoSkin™ enhances survival rates, long-term skin outcomes, and, ultimately, quality of life of burn survivors.

## Introduction

The skin, the body's largest organ, serves as a vital barrier against external threats, protecting against microorganisms, dehydration, ultraviolet radiation, and mechanical damage. Also, the intact skin is a unique interface between the inner and outer realm of human beings and so harbors countless ways to bidirectionally convey and perceive myriads of signals that contribute to many colors of the spectrum between naked survival and sumptuous quality of life.

Severe burn injuries compromise these protective and communicative functions, often leading to life-threatening complications and life-long disabilities. Despite advancements in diagnostics and treatment, burns remain a significant global health issue. According to a 2023 report by the WHO, an estimated 180,000 people die from burns every year and non-fatal burn injuries are a leading cause of morbidity.^1^

Over the past two decades, survival rates in the acute phase of severe burn injuries have markedly improved, even for patients with massive burns of up to 95% of total body surface area (TBSA). This progress is largely attributed to advancements in intensive care medicine that have optimized hemodynamic and homeostatic stabilization, nutritional management, infection control, and respiratory support, as well as the introduction of early burn eschar excision as a surgical standard. These developments have significantly reduced early mortality. However, with more patients surviving the initial phase, favorable long-term recovery now, and more than ever before, critically depends on rapid and effective wound coverage to minimize complications, notably devastating scarring, and to improve functional outcomes.

Yet, achieving rapid and definitive wound coverage remains a major challenge, particularly in extensive burns. Limitations are the availability of donor sites, which decrease as burn size increases, as well as the quite restricted expansion options for autologous split-thickness skin grafts (STSG). The more mechanical standard expansion techniques (mesh or Meek micrograft, from 1:2 to 1:9) augment the area of coverage, the worse becomes scar quality, ultimately contributing to poor quality of life. Since the 1980s, CEA sheets have become an additional treatment option, but their clinical application has been hampered by inconsistent graft take, vulnerable skin, and a high incidence of hypertrophic scar formation. More complex cultured two-layered autologous skin substitutes, incorporating in addition to keratinocytes also fibroblasts and extracellular matrix components, have been developed and used on patients since the late 1990s. However, to our knowledge, no prospective, randomized phase I or phase II clinical trials have assessed their safety and efficacy.

For 25 years, the Tissue Biology Research Unit at the University Children's Hospital Zurich, Switzerland, has focused on tissue engineering of autologous skin substitutes. In 2014, a prospective, randomized, controlled phase I clinical trial was conducted successfully to evaluate the safety of a novel two-layer, dermo-epidermal, autologous skin substitute (termed denovoSkin™), consisting of a hydrogel matrix embedded with autologous fibroblasts and covered with confluent autologous keratinocytes.

The aim of this study is to evaluate safety and efficacy of an autologous, bio-engineered skin substitute (denovoSkin™) by reporting the results of a first prospective, randomized, controlled phase IIb clinical trial comparing it with the current gold standard which is autologous meshed STSG in an international multicenter setting, with a 12-month follow-up.

## Methods

### Materials

denovoSkin™ was manufactured as previously described in the GMP-certified clean room facility of Wyss Zurich Translational Center, Zurich, Switzerland, until February 2020, thereafter in the GMP-certified clean room facility of CUTISS AG, Schlieren, Switzerland.^2^ Briefly, a split-thickness skin biopsy of about 4 cm^2^ (mean 4·49 cm^2^) was harvested from the patient. Fibroblasts and keratinocytes were isolated and expanded. Fibroblasts were submerged in an acid-soluble bovine collagen type I hydrogel (Symatese, Chaponost, France). After plastic compression and a cultivation period of 5–8 days, keratinocytes were seeded onto the graft. After additional cultivation of 5–8 days, one to two sheets of denovoSkin™ (each 45 ± 4 cm^2^, thickness 0·5–2·0 mm) was shipped in temperature-controlled conditions to the study sites under Good Distribution Practice. Limitation to two grafts per patient was based on ethical and study quality considerations for performing a phase II clinical trial. The mean production time of denovoSkin™ from skin biopsy to grafting took 34·5 days (SD 6·2 days, median 35·0 days, minimum 28 days, maximum 50 days).

### Study design

**Fig. 1:**
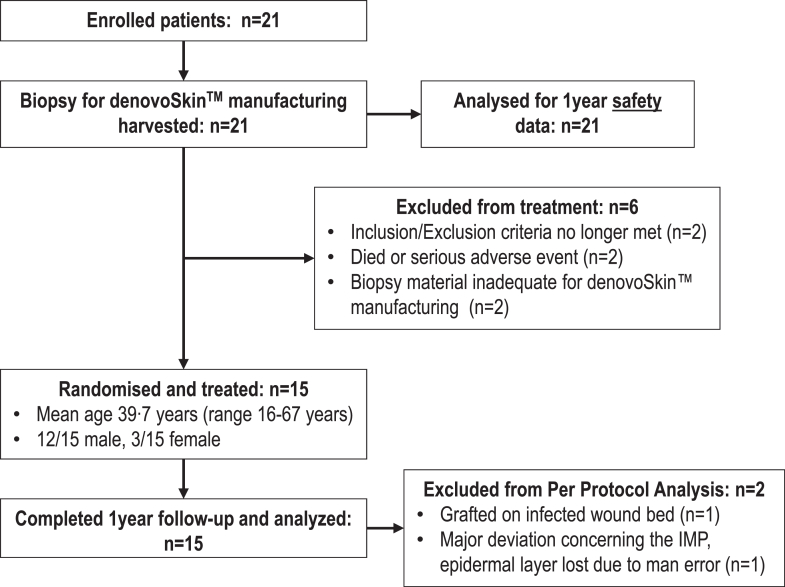
Trial Cohort flow-Chart.

This phase IIb, prospective, intra-patient randomized, controlled, open-labeled, multi-center clinical trial to evaluate safety and efficacy of an autologous bio-engineered dermo-epidermal skin substitute to treat deep dermal and full-thickness burns in adults and adolescents in comparison to autologous STSG, the established gold standard for treating burn wounds, was conducted at four burn centers in Switzerland, the Netherlands, and Italy. Participants flow is depicted in Fig. 1.

The first patient was enrolled in March 2018. The analysis of the 1-year data was conducted after 15 patients had reached data maturity for the primary endpoint. Recruitment to the trial was completed in July 2022. This study included an *intra-*patient comparator rather than a between-patient matched control design to avoid potential variability associated with individual physiological and healing differences that may occur between patients.

## Ethics

This trial was conducted in accordance with the ethical principles of the Declaration of Helsinki (2013) and is consistent with the International Council for Harmonization of Technical Requirements for Pharmaceuticals for Human Use (ICH), Good Clinical Practice (GCP) guidelines (ICH E6), and applicable local regulatory requirements and laws. The study protocol was approved by all national authorities (NL: CCMO NL62252.000.17; IT: AIFA 28428; CH: Swissmedic ID 2017TpP2001) and responsible ethics committees (NL: NL62252.000.17; IT: CS2/1421; CH: KEK-ZH ID 2016-00494). This trial is registered with ClinicalTrials.gov (NCT03227146).

Each patient or his/her legally authorized representative provided written informed consent prior to the conduct of any study-related procedures in accordance with national applicable laws. Whenever possible, verbal assent was sought from participants when consent was obtained from a legally authorized representative. Additionally, as soon as the patients were able to provide consent themselves, full written informed consent was obtained.

### Patients

In general, two areas of a same included patient with a debrided area were randomized to be covered either with denovoSkin™ or with a conventional meshed STSG. More precisely:

The trial population consisted of adolescents and adults with deep partial and full-thickness burns requiring surgical removal of burned tissue and then autologous wound coverage. Inclusion and exclusion criteria are listed in the Supplementary materials. Sex was reported by participants at enrolment, based solely on sex assigned at birth. The response options were “female” and “male.” Enrolled patients were grafted with denovoSkin™ according to the study protocol while conventional STSG served as controls.

The study comprised a limited number of patients. Patients enrolled were all ≥12 years old, male and female, and suffered from deep dermal and/or full-thickness burns requiring surgical wound coverage. Burn extension of >20% TBSA was taken as guideline but was not a formal inclusion criterion.

### Randomization and masking

Web-based randomization for the treatment allocation of the two study areas of each study patient was performed during surgery in the electronic case report form (eCRF). The randomization scheme was “block randomization” without stratification. Two wound areas of comparable depth and size were identified by the surgeon based on study protocol pre-defined metrics and randomized to receive either denovoSkin™ or STSG (meshed 1:3) such that every patient simultaneously received both treatments for comparison. To avoid any bias in wound bed preparation, surgeons were masked for treatment allocation until application of the two treatments. Due to the marked difference in texture and appearance of denovoSkin™ and STSG, blinding of surgeons and participants was not feasible.

### Procedures

All enrolled patients received one to two grafts of denovoSkin™ (size each 45 ± 4 cm^2^, thickness 0·5–2·0 mm) and a meshed (1:3) STSG of the same size as control onto the intra-patient randomized study areas. Grafting was performed 28–50 days after biopsy harvesting (mean 34·5 days, SD 6·2 days). For wound bed preparation, dermal templates were not allowed for methodological reasons in both experimental and control areas, except for Integra® Dermal Regeneration Template. All visit timepoints were defined relative to the grafting. The first dressing change was performed 6–10 days (mean 7 days) post-grafting; thereafter, standard graft checks were performed at day 21 ± 2 and day 28 ± 3. The protocol-defined follow-up visits were conducted 2, 3, 6, and 12 months post-grafting. After 12 months, further visits were considered as long-term follow-up visits and were conducted up to 36 months post-grafting. There was no difference in wound care or long-term therapy between the experimental and the control area.

### Safety

Safety assessments included monitoring of adverse events, clinical laboratory evaluation, and clinical and microbiological signs of infection.

### Outcomes

#### Primary efficacy outcome—Expansion rates/donor site sparing capacity

The primary endpoint was defined as the expansion rate, i.e., the ratio of the initial biopsy size to the grafted wound area successfully closed 4 weeks after transplantation equally for denovoSkin™ and for STSG. More precisely, assessed as the ratio of denovoSkin™ grafted area with take versus harvested biopsy site, and STSG grafted area with take versus harvested donor site at day 28 ± 3 post-grafting hypothesizing that the experimental treatment will result in a better ratio.

#### First secondary efficacy outcome—Wound closure

Assessed as percentage of wound epithelialization 3 months post-grafting.

#### Secondary efficacy outcome—Scar quality and functionality 3, 6, and 12 months post-grafting

Skin elasticity was assessed using the Cutometer®, skin color was measured using the DSM ColorMeter®, and scar quality and functionality assessment was performed using the validated POSAS (Patient and Observer Scar Assessment Scale) questionnaire (10-point scale), as well as photography.^3^

#### Safety outcomes

All adverse events (AEs) and all serious adverse events (SAEs) occurring from biopsy harvesting until the 1-year assessment were fully investigated and documented.

#### Exploratory outcomes

Graft take was visually assessed at first dressing change (6–10 days post-grafting) as percentage of viable denovoSkin™ or STSG, time to full epithelialization and Quality of Life (QoL) (assessed by the European Quality of Life 5 Dimensions generic tool for patient-reported outcomes measurement (EQ-5D), and the Burns Specific Health Scale-Brief questionnaire (BSHS-B)) at visits 8–12 (3 and 6 months post-grafting, and 1, 2, and 3 years post-grafting) were also evaluated.

### Statistical analysis

In this study, we report uncorrected p-values. Our analyses were based on a limited number of a priori hypotheses, rather than an exploratory screen of a large space of potential associations. While we acknowledge that multiple testing corrections are commonly used to control familywise error rates, such corrections can be overly conservative in this context and risk obscuring potentially meaningful effects. To provide a balanced interpretation, we emphasize effect sizes and confidence intervals alongside p-values, allowing readers to evaluate both the magnitude and precision of observed effects. These results should therefore be interpreted as evidence consistent with our theoretical predictions, while recognizing the need for replication in independent datasets. The following assumption was used for statistical calculations: The ratio of covered surface area to biopsy site/donor site surface area at 4 weeks post grafting was expected to be at least 145% higher for denovoSkin™ than for STSG (corresponding, for instance, to ratios of 7·35 and 3·00, respectively). Assuming a standard deviation of 1·00 for the paired difference between the 2 log (ratios), a target sample size of 12 evaluable patients was calculated to be required in order to achieve a power of 80% to detect a treatment effect corresponding to an increase of 145% with denovoSkin™ (i.e., to a difference of 0·90 on a logarithmic scale) when using a one-sample t-test at two-sided significance level of 5%. A claim of study success was to be made when the two-sided test would pass the significance level of 0·05. All secondary endpoints were to be tested in a strictly exploratory fashion only.

The following analysis sets were defined for this study: Safety Set (SAF, all patients who underwent a pre-treatment biopsy harvest); Grafting Safety Set (GSAF, all patients of the SAF who underwent skin grafting procedures); Full Analysis Set (FAS, all patients who underwent a pre-treatment biopsy harvest); Modified Full Analysis Set (mFAS, all randomized patients who underwent skin grafting procedures). Any analysis performed on the mFAS was to consider the treatment as randomized; per protocol Set (PPS, all patients of the mFAS without major protocol violations that might impact the primary efficacy endpoint). The efficacy analysis was primarily performed on the mFAS. Supportive analyses were performed on the PPS. The safety analysis was performed on the SAF and GSAF, as applicable for the variables assessed.

The statistical analysis was performed using the SAS (Statistical Analysis System) software for WINDOWS, Version 9·4.

For the primary analysis, two tests (one-sample t-test and Wilcoxon signed-rank test) were pre-specified in the Statistical Analysis Plan (SAP) and were conducted. As also described in the SAP, the Wilcoxon test is more robust, in particular for small analyses sets, and less influenced by the values that were used for imputation of missing data. Hence, in case the two tests yield contradicting results, it was decided (in the SAP) to consider the Wilcoxon test as the primary analysis.

An independent data safety monitoring board was established for this trial. Board members were fully independent from the clinical trial and were not involved in any trial-related procedures and data analysis (Trial Registration under clinicaltrials.gov identifier NCT03227146).

### Role of the funding source

The funder Wyss Zurich Translational Center had no role in study design, data analysis, data interpretation, or writing of the report, but was involved in data collection. The funder CUTISS AG of the study was involved in study design, data collection, data analysis, data interpretation, and writing of the report.

## Results

Between March 26, 2018, and July 26, 2022, 21 patients were recruited at the University Children's Hospital Zurich, Switzerland; the University Hospital Zurich, Switzerland; Red Cross Hospital Beverwijk, The Netherlands; Maasstad Ziekenhuis Rotterdam, The Netherlands; and the Azienda Ospedale Università Padova, Italy. 21 underwent a biopsy, 15 were grafted, and 13 were included in the per protocol set (PPS) (Fig. 1). Of the six patients excluded from the treatment, two patients passed away following biopsy harvesting, prior to undergoing denovoSkin™ transplantation. Briefly, the demographic data are shown in Table 1. The mean total body surface area (TBSA) burned (deep second degree and third degree burns requiring necrosectomy and grafting) was 46·2% (range 21–80%) (Table 1). The mean area expected still lacking definitive skin coverage, i.e., skin defects to be grafted, 4 weeks post-burn was 1098·1 cm^2^ (range 90–6000 cm^2^). The causes of thermal injuries were fire (n = 13), scalds (n = 3), electricity (n = 1), and “unknown” (n = 4).

**Table 1:** Study patients demographics.

	Randomized and treated patients (n = 15)	Enrolled patients (n = 21)
Age [years]		
Mean (SD)	40 (16)	42 (17)
Median	40	40
Q1; Q3 (IQR)	21; 55 (34)	31; 55 (24)
Min; Max	16; 67	16; 74
Gender n (%)		
Female	3/15 (20%)	4/21 (19%)
Male	12/15 (80%)	17/21 (81%)
Race n (%)		
Caucasian	14/15 (93%)	19/21 (91%)
Black	0/15 (0%)	1/21 (5%)
Other	1/15 (7%)	1/21 (5%)
Total body surface area burned (%)		
Mean (SD)	46 (17)	46 (18)
Median	45	42
Q1; Q3 (IQR)	33; 60 (27)	32; 60 (28)
Min; Max	21; 80	21; 80
Area open at 4 w post-burn [cm^2^]		
Mean (SD)	1098 (1684)	896 (1463)
Median	200	200
Q1; Q3 (IQR)	100; 1500 (1400)	100; 1000 (900)
Min; Max	90; 6000	90; 6000
Area covered with denovoSkin™ [cm^2^]		
Mean (SD)	64 (14)	–
Median	65	–
Q1; Q3 (IQR)	50; 74 (24)	–
Min; Max	39; 91	
Area covered with control STSG [cm^2^]		
Mean (SD)	82 (36)	–
Median	81	–
Q1; Q3 (IQR)	50; 101 (51)	–
Min; Max	35; 177	
Area of biopsy site for denovoSkin™ manufacturing [cm^2^]		
Mean (SD)	4 5 (1 3)	–
Median	4	–
Q1; Q3 (IQR)	4; 4 (0)	–
Min; Max	4; 8	
Area of STSG donor site for control area [cm^2^]		
Mean (SD)	56 (33)	–
Median	49	–
Q1; Q3 (IQR)	35; 82 (47)	–
Min; Max	12; 130	

n = number of patients; SD = standard deviation; Min = minimum; Max = maximum.

**Fig. 2:**
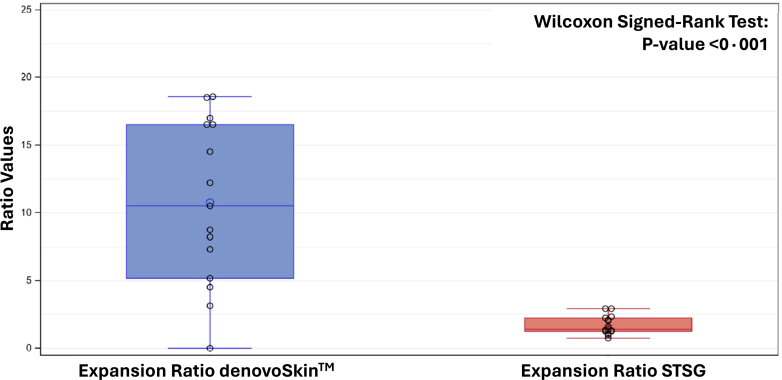
Expansion ratios for denovoSkin™ (blue) and STSG (red) 4 weeks post grafting (n = 15).

Regarding the primary efficacy outcome, the mean expansion ratio for denovoSkin™ was 10·76 (95% CI: [7·43; 14·10], range 0·0–18·6). For STSG, it was 1·70 (95% CI: [1·32; 2·08], range 0·8–2·9). The median ratio of the two expansion ratios was 7·0, indicating that the ratio for denovoSkin™ was 7·0 times as large as that for STSG (Fig. 2).

The mean ratio of the 2 expansion ratios (denovoSkin™/STSG) was 7·41 (95% CI: [4·71; 10·10], range 0·0–17·3).

In the PPS the mean expansion ratio for denovoSkin™ was 12·02 (95% CI: [8·80; 15·24], range 3·1–18·6), and for STSG it was 1·68 (95% CI: [1·25; 2·12], range 0·8–2·9). The mean ratio of the 2 expansion ratios (denovoSkin™/STSG) was 8·25 (95% CI: [5·47; 11·04], range 1·5–17·3).

The assessment of the percentage of epithelialization (wound closure) 3 months post grafting revealed no difference between denovoSkin™ and STSG (100% epithelialization for both).

**Fig. 3:**
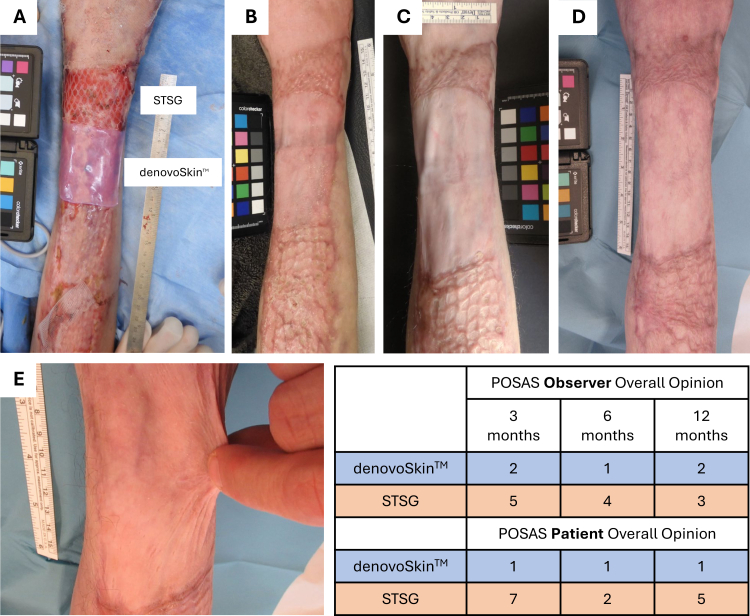
Photographic documentation of a study case, where one sheet of denovoSkin™ is grafted onto the right forearm. The control area with STSG is adjacent and distal to the experimental area, between the denovoSkin™ area and the right hand. (A) Day 0 = randomized grafting of denovoSkin™ and STSG. The two areas are labeled accordingly (in the following pictures B-E, the orientation is identical to picture A). (B) 3 months post-transplantation (PT). STSG shows the typical initial phase of graft maturation with redness (hyperperfusion) and hypertrophic scar formation while denovoSkin™ does not exhibit these features and already appears like a matured, i.e., end stage graft. (C) 6 months PT and (D) 12 months PT. Grafted areas demonstrate about the same findings as at 3 months: STSG is still an immature hypertrophic scar while denovoSkin™ is smooth and has not scarred and approximates normal skin. (E) Excellent pliability and elasticity of denovoSkin™ 6 months PT, in contrast to the control area where pinching the skin was not possible due to its stiffness. POSAS Observer Overall Opinion and Patient Overall Opinion are shown for this case in the inserted table. Evident growth of the denovoSkin™ area over a 1-y time can be observed in pictures A-D (from 7 cm length in A to approximately 14 cm in D), whereas marked shrinking can be observed for the control area (from 5 cm in A to approximately 3 cm in D).

Scar quality and functionality was assessed 3, 6, and 12 months post-grafting (Fig. 3). The Cutometer® based analysis of elasticity and pliability as well as the DSM ColorMeter® for color assessment did not provide any large differences between denovoskin™ and STSG, and unwounded skin.

**Fig. 4:**
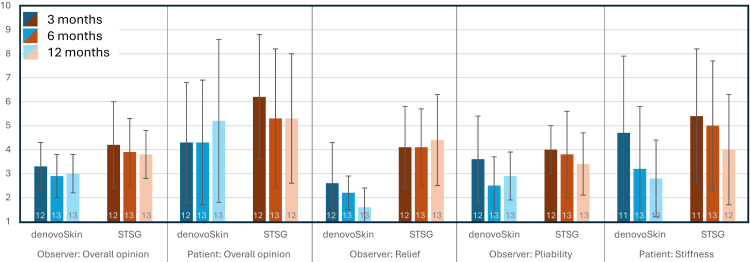
Overview of POSAS data, graph indicates mean values with standard deviations of the assessments 3, 6, and 12 months post-transplantation. All questions are assessed using a 10-point scale (1 = normal skin, 10 = worst scar imaginable), generally, a value between 1 and 3 is considered a good scar.

For the POSAS 2·0, each of the questions is assessed on a 10-point ordinal scale (1–10), whereas 1 = normal skin, and 10 = worst scar imaginable. The POSAS Observer Overall Opinion for denovoSkin™ was numerically lower, so closer to normal skin than to STSG (3 months: p = 0·043, 6 months: p = 0·078, 12 months: p = 0·074). Regarding the relief score (observer), the difference between normal skin and denovoSkin™ was smaller than between normal skin and STSG (p = 0·005 at 6 months, and p < 0·001 at 12 months). The pliability score (observer) was more favorable for denovoSkin™ versus STSG (for overview of POSAS data see Fig. 4). There was little evidence for a difference between experimental and control areas for color, thickness, and textural regularity at any visit. POSAS ratings supported the appearance of the scar quality observed on the photographic documentation (table insert in Fig. 3). The Patient Overall Opinion appeared to be favorable for the denovoSkin™ treated area at month 12 compared to the control area (p = 0·016). In the patient's assessment for the stiffness score (difference from normal skin), the denovoSkin™ rating was numerically better at 6 months (p = 0·017) and 12 months (p = 0·047) post-grafting. There was little evidence for any difference in pain, itching, color, thickness, and regularity.

The mean ratio between graft take and grafted area for denovoSkin™ was 72·57% (range 0%–100%). For STSG, it was 90·36% (range 59·3%–100%) (p = 0·008) at visit four (6–10 days post grafting). The median time to complete epithelialization (i.e., full wound closure) was 63 days for denovoSkin™ (95% CI: [57; 187 days]) and 24 days for STSG (95% CI: [21, 57 days]). QoL seemed to improve during the period between 3 months and 1 year post-grafting. However, no distinction could be made between denovoSkin™ and STSG since the study settings rely on an intra-patient comparison and on a small study area.

Local infections of both denovoSkin™ and meshed STSG areas were assessed clinically and microbiologically at three consecutive dressing change visits. Three patients showed infections in the denovoSkin™-grafted area; however, all tested negative by 4 weeks post-grafting. Two Procedure Emergent Adverse Events (PEAEs) related to study treatment were reported for two patients (9·5%), none of which was serious. Both two PEAEs occurred after the grafting procedure (i.e., one case of hematoma 1 week after grafting, and one case of a necrotic zone at the experimental area 1 week after grafting) (Table 2). A total of 164 adverse events were observed (Table 3). The vast majority were related to the underlying pathology of severe burn injury, which commonly affects multiple organ systems, and to pre-existing comorbidities. Only two of these (hematoma and partial skin necrosis) could be related to denovoSkin™. They rapidly healed spontaneously. Most of the hematology data recorded at the screening visit, grafting visit, and 1 week post-grafting were normal or slightly abnormal but of no clinical relevance.

**Table 2:** Patients with PEAEs related to treatment by SOC and PT.

System organ class	Biopsy period n = 21	Post-grafting period n = 15	Total safety period n = 21
Preferred term	n (%) nae	n (%) nae	n (%) nae
Any PEAE related to treatment	0	2 (13%) 2	2 (10%) 2
Injury, poisoning and procedural complications	0	1 (7%) 1	1 (5%) 1
Subcutaneous hematoma	0	1 (7%) 1	1 (5%) 1
Skin and subcutaneous tissue disorders	0	1 (7%) 1	1 (5%) 1
Skin necrosis	0	1 (7%) 1	1 (5%) 1

n = number of patients in the analysis set; n = number of patients with an event; nae = number of adverse events; SOC = system organ class; PT = preferred term; PEAE = Procedure-Emergent Adverse Event.

**Table 3:** Patients with PEAEs by SOC and PT, reported by ≥3 patients in any period.

System organ class	Biopsy period N = 21	Post-grafting period N = 15	Total safety period N = 21
Preferred term	n (%) nae	n (%) nae	n (%) nae
Any PEAE	17 (81%) 86	12 (80%) 78	17 (81%) 164
Infections and infestations	12 (57%) 33	11 (73%) 27	14 (67%) 60
Pneumonia	3 (14%) 3	3 (20%) 4	6 (29%) 7
Enterococcal infection	3 (14%) 3	0 (0%) 0	3 (14%) 3
Pseudomonas infection	3 (14%) 3	0 (0%) 0	3 (14%) 3
Wound infection bacteria	2 (10%) 3	3 (20%) 4	3 (14%) 7
Injury, poisoning, and procedural complications	4 (19%) 4	9 (60%) 13	10 (48%) 17
Skin scar contracture	0 (0%) 0	4 (27%) 6	4 (19%) 6
Psychiatric disorders	9 (43%) 9	3 (20%) 4	10 (48%) 13
Delirium	5 (24%) 5	0 (0%) 0	5 (24%) 5
Insomnia	3 (14%) 3	1 (7%) 1	4 (19%) 4
General disorders and administration site conditions	6 (29%) 7	5 (33%) 7	8 (38%) 14
Pyrexia	5 (24%) 5	0 (0%) 0	5 (24%) 5
Impaired healing	0 (0%) 0	4 (27%) 6	4 (19%) 6
Metabolism and nutrition disorders	3 (14%) 4	3 (20%) 3	6 (29) 7
Vascular disorders	3 (14%) 6	3 (20%) 4	6 (289%) 10
Skin and subcutaneous tissue disorders	2 (10%) 2	4 (27%) 8	5 (24%) 10
Pruritus	1 (5%) 1	3 (20%) 3	4 (19%) 4
Nervous system disorders	2 (10%) 3	3 (20%) 3	4 (19%) 6
Respiratory, thoracic, and mediastinal disorders	3 (14%) 6	2 (13%) 2	4 (19%) 8
Blood and lymphatic system disorders	3 (14%) 3	0 (0%) 0	3 (14%) 3
Cardiac disorders	1 (5%) 2	2 (13%) 2	3 (14%) 4
Renal and urinary disorders	1 (5%) 1	2 (13%) 2	3 (14%) 3

N = number of patients in the analysis set; n = number of patients with an event; nae = number of adverse events; SOC = system organ class; PT = preferred term; PEAE = procedure-emergent adverse event.

## Discussion

This prospective, multicenter, intra-patient randomized, open-labeled, controlled phase IIb clinical trial indicates that transplantation of the bio-engineered autologous skin, denovoSkin™, to treat deep dermal and full-thickness burns is primarily safe. It compares favorably with the control area yielding a significantly greater expansion rate from biopsy/donor site to grafted area and from a clinical point of view resulted in close to normal skin, in particular devoid of clinically relevant scarring. Such significant progress regarding notorious key problems has, to our knowledge, not previously been reported in a formal prospective clinical trial.^4^^,^^5^ These findings could have implications for clinical practice. The key aspects of this study are discussed below.

Safety was evaluated based on the occurrence of adverse events, clinical laboratory parameters, and signs of infection. Throughout the study, 164 adverse events were recorded, but only two were attributed to the product itself: one hematoma and one partial skin necrosis, both of which resolved spontaneously under conservative management. Other serious adverse events were observed in low numbers and were linked to the severity of the burn injury (e.g., infections, respiratory complications, and systemic disorders) rather than to the skin substitute.

Clinical and laboratory parameters, assessed at multiple time points, remained largely within normal or slightly abnormal ranges without clinical relevance. Wound infections, a common problem in burn patients and a specific focus of our analysis, occurred in three patients (20·0%) in the denovoSkin™ area and in one patient (6·7%) in the STSG area (ns). These findings show that denovoSkin™ does not increase infection susceptibility compared to conventional grafting.^6^

The expansion ratio assessed as the ratio of grafted area with take versus harvested biopsy found for denovoSkin™ (transplanted as sheet graft) was seven times higher than the one for a 1:3 meshed STSG (p < 0·001), a magnitude of difference not reported so far in a prospective clinical trial such as ours. Obviously, the higher the expansion ratio, the more donor sites are spared.

The true dimension and meaning of these high expansion ratios for bio-engineered skin must be set into context: The introduction of early and radical excision of all dead skin was a key advancement in burn surgery about 30 years ago, but brought about very large skin defects in particular in case of extended deep burns.^7^, ^8^, ^9^ These should be rapidly and definitively grafted with autologous skin, an almost impossible task in the absence of sufficient donor sites. To combat, efforts focused on mechanical expansion techniques, such as mesh and MEEK grafting. However, these methods were, notoriously, limited by scarce donor sites, modest expansion ratios of at best 1:9, and poor long-term results.^10^ Adversely, repeated harvesting of STSG resulted in delayed donor site healing, even scarring of these, and increased patient morbidity.^11^^,^^12^

Alternatively, cell expansion methods, such as cell culturing and bio-engineering used in this study, offer high expansion ratios by design. Cultured epithelial autografts (CEAs) can achieve an expansion ratio of up to 10,000-fold, meaning that a 1 cm^2^ skin biopsy has the potential to generate approximately 1 m^2^ of epithelial grafts within 2–3 weeks under optimal culture conditions.^13^^,^^14^ Culturing of isolated keratinocytes into confluent layers and transplantable sheets, a veritable milestone, was first realized by Rheinwald and Green in 1975.^15^ Boyce later demonstrated clinically that more complex autologous skin substitutes, i.e., fibroblasts seeded in a matrix combined with keratinocytes, enabled donor skin expansion of over 100-fold, hypothetically allowing full body resurfacing with less than 1% TBSA of donor skin.^16^^,^^17^

With sizeable facilities, the quantity of usable denovoSkin™ reaches far beyond the framework of this study. While here, due to regulatory reasons, only two denovoSkin™ grafts (max. 98 cm^2^) per patient were allowed, a 4 cm^2^ biopsy as taken in this trial could, possibly, yield enough bio-engineered skin for total body coverage of an adult patient. The tenable hypothesis of being able to supply high amounts of bio-engineered skin for one single patient in due time might induce a fundamental shift of paradigm. Burn surgery and other types of reconstructive surgery might no longer rely on traditional donor sites but benefit from large supplies delivered by automated skin factories currently being developed.

Several formally approved compassionate-use cases support this theory. Patients with massive burns of up to 95% TBSA, were grafted with denovoSkin™ in multiple sessions (up to 28% TBSA). Results were highly encouraging, with graft take rates between 70% and 90%, expansion ratios from 56 to 193×, and rapid graft maturation without significant scarring.^18^, ^19^, ^20^ Obviously, the more denovoSkin™ is used, the lesser conventional donor sites are (re)used, and the iatrogenic burden of repetitive STSG (re)harvesting is reduced accordingly. For patients with massive burns, large scale application of denovoSkin™ may contribute to improved outcomes and to an enhanced survival rate.^19^

Generally speaking, and as exemplified by our compassionate use patients, major burns are typically managed according to a structured surgical masterplan. We have already shown that even multiple denovoSkin™ deliveries can be reliably planned and seamlessly inserted into treatment lines.^18^^,^^19^

This approach could be relevant not only for burn surgery but also for elective reconstructive procedures. Regarding the latter, a biopsy is taken on a planned outpatient basis, and the surgical intervention scheduled once denovoSkin™ is ready (clinicaltrials.gov NCT03394612).

In addition to the donor site sparing potential (primary endpoint assessed at 4 weeks post grafting), to assess the efficacy of denovoSkin™, we evaluated wound closure, scar quality, and QoL. Three months post-transplantation, epithelialization rates of denovoSkin™ and STSG were comparable. However, complete epithelialization of denovoSkin™ occurred slower. This is likely due to the thicker dermal compartment of denovoSkin™ that initially depends on diffusion-based nourishment, followed by neovascularization, before epithelial maturation is completed. In contrast, STSG is much thinner and contains a normally stratified epidermis and a normal dermal capillary network that rapidly connects to wound bed capillaries through inosculation.

In a retrospective case series, Boyce also described a significantly (p < 0·05) lower engraftment rate of a cultured skin substitute compared to STSG.^21^

Importantly, despite delayed epidermal maturation, denovoSkin™ was not more susceptible to infection than STSG. This may be attributed to the presence of a confluent multi-layered epidermis and dermal fibroblasts instantly providing a protective barrier.^22^

To assess scar quality, we examined specific parameters such as elasticity (Cutometer®) and pigmentation (ColorMeter®) and used the Patient and Observer Scar Assessment Scale (POSAS). Taken together, our findings show that denovoSkin™ matures faster than STSG and, unlike STSG, was associated with minimal clinically relevant scarring.^23^

Elasticity and pliability measurements using the Cutometer® did not yield conclusive differences between denovoSkin™, STSG, and unwounded skin, likely due to small patient numbers and inter-center variability. However, POSAS assessments and photographic documentation revealed clear advantages of denovoSkin™ over STSG, particularly regarding pliability, relief, stiffness, texture, and aesthetic appearance.

Interestingly, despite lacking melanocytes, denovoSkin™-treated areas did not show significant color discrepancies compared to STSG in POSAS, as assessed by both patients and observers (most patients were Caucasian). However, ColorMeter® readings confirmed hypopigmentation in denovoSkin™-treated areas, although no clear differences between treated and control sites were observed at any time point.

We recently published an illustrative compassionate use case of an infant highlighting the two fundamentally different biological behaviors of denovoSkin™ versus STSG. Areas grafted with cultured skin exhibited normal elasticity and growth over 2 years, while adjacent STSG massively scarred and mandated early reconstructive surgery.^20^ This case suggests that denovoSkin™ may help to minimize subsequent scar revisions, particularly in growing children.

QoL assessments showed general improvement between 3 months and 1 year post-grafting. Of note, due to the intra-patient comparison, no differences could be evaluated between denovoSkin™ and STSG.

There are a number of limitations to cast light on. First, under Orphan Drug Designation for deep partial and full-thickness burns, the protocol allowed only a small number of patients, recruited in four different centers. More data, however, have been and will be generated in reconstructive surgical, in compassionate use patients, as well as in an ongoing phase III clinical trial involving 70 patients.

Second, a production time of approximately 3–4 weeks from biopsy to graft availability is required to bioengineer the dermo-epidermal tissue. While this interval might seem a limitation, it can be efficiently bridged with temporary wound coverage. The use of allografts or dermal templates such as Polynovo™ or Integra™ represents an established approach to prepare a well-vascularized and clean wound bed for subsequent denovoSkin™ grafting.^24^, ^25^, ^26^ Moreover, there is growing evidence that this sort of bridging is enhancing scar quality and so improves outcomes.^27^^,^^28^

Third, regulatory constraints for this trial prevented the use of more than two transplants. However, results from compassionate use patients receiving large quantities of denovoSkin™ support the conclusions drawn here.^18^, ^19^, ^20^

Finally, the product investigated is devoid of melanocytes and thereby causes dyspigmentation. While this was less evident in the predominantly Caucasian population studied here, it could have a more noticeable impact in dark-skinned individuals. Further research directions include analyses of long-term results of denovoSkin™ in various clinical settings as well as development of additionally pigmented and vascularized dermo-epidermal skin substitutes in prospective clinical trials.^29^^,^^30^

The key results of this prospective, randomized, controlled phase IIb clinical trial show that denovoSkin™ sheet grafting compares favorably with the current gold standard, i.e., meshed STSG grafting. Within a few months after transplantation, this novel bio-engineered product yields functionally and aesthetically close to normal skin, devoid of clinically relevant scarring. An ongoing laboratory production of denovoSkin™ offers abundant supply for multiple subsequent grafting sessions. Thus, using denovoSkin™ substantially reduces the requirement for STSG and thereby spares conventional donor sites. This approach has the potential to influence both burn and reconstructive surgery and may contribute to future developments in skin replacement strategies.

## Contributors

CS, MM, DM, FH, ER had the idea for and designed the study. MM, CS, FH, EM, BSK, KN, JP, DR, CvdV, BA, DM, KM, MF, JB, SB contributed to data collection and assembly. MM, CS, FH, DM, KM, SB interpreted and analyzed data. MM, CS, FH, DM, KM, JB, MF, SB had full access to all the data in the study, and all these authors accessed and verified the underlying data. All authors wrote and reviewed the report, and approved the final version for submission. All authors had final responsibility for the decision to submit for publication.

## Data sharing statement

Deidentified individual participant data that support the findings of this study may be made available to qualified researchers upon written request and subject to review. If access is granted, synopses of key supporting documents (protocol, statistical analysis plan, informed consent form) will also be provided. Data will be available from the date of publication for a period of 5 years. Requests should be directed to [daniela.marino@cutiss.swiss]. Data access will be provided only upon approval of a scientifically valid research proposal and execution of a data access agreement. No additional data or investigator support will be provided. The sponsor reserves the right to decline requests at its discretion.

## Declaration of interests

EM, BSK, SB, JP, DR, CvdV, BA, MF, and JB declare that they have no conflict of interest. CS, FH and KM are shareholders of CUTISS AG. FH is an employee of CUTISS AG and was the project co-coordinator at the start-up accelerator Wyss Zurich for the project “denovoSkin”. MM, DM, and ER are shareholders and Board Members of CUTISS AG. (MM is President of the Board, DM is CEO.)

For the phase II clinical trials with denovoSkin™ (NCT03229564, NCT03227146, NCT03394612), ER was the trial sponsor representative at University of Zurich (2017–2022), CS was trial Coordinating Investigator, and MM (2017-06/2020), SB (from 07/2020), EM, and BSK (from 01/2022) were trial principal investigators. Since 2023, MM serves as a medical consultant and since April 2025 also as a phase III medical trainer for CUTISS AG. EM is a CUTISS trainer supporting and mentoring principal investigators of the phase III clinical trial.

The institution of the KN, MF, JB, SB has received a Writing Support, captured in an Agreement between Cutiss AG and the University Children's Hospital Zurich.

## Appendix A Supplementary data

Study Protocol
