# Supplementary material for: Safety and efficacy of bio-engineered, autologous dermo-epidermal skin grafts in adolescent and adult burn patients: 1-year results of a prospective, randomized, controlled, multicenter phase IIB clinical trial
Source: eClinicalMedicine. 2025 Nov 28;90:103665. doi: 10.1016/j.eclinm.2025.103665 (PMC12702297; doi:10.1016/j.eclinm.2025.103665)
Supplement: Study Protocol [file mmc1.pdf]

CUTISS
personalized skin

# CLINICAL STUDY PROTOCOL

| | |
|---|---|
| **Protocol Title:** | **A Phase IIb, prospective, intra-patient randomised controlled, multicentre study to evaluate the safety and efficacy of an autologous bio-engineered dermo-epidermal skin substitute (EHSG-KF) for the treatment of partial deep dermal and full-thickness burns in adults and adolescents in comparison to autologous split-thickness skin grafts (STSG)**<br><br>**With a sub-study at the sites a) Azienda Ospedaliera di Rilievo Nazionale Antonio Cardarelli (Italy), b) Santobono Napoli (Italy), and c) Azienda Ospedale Università Padova (Italy)**<br>**SUBSTUDY TO PROTOCOL TBRU-dS-BA-PIIb-Study**<br><br>**Title: An open-label, prospective, non-randomised, non-controlled study to evaluate the survival of the adult and adolescent patients with deep partial and full-thickness burns in a life-threatening situation treated with EHSG-KF, and to evaluate the safety and efficacy of EHSG-KF** |
| **Product:** | EHSG-KF (Synonym "denovoSkin") |
| **Short Title/Study ID:** | TBRU-dS-BA-PIIb |
| **Protocol Version:** | Version 12, 2022-10-13 |
| **EudraCT Number:** | 2017-002460-41 |
| **Sponsor:** | CUTISS AG<br>Grabenstrasse 11<br>8952 Schlieren, Switzerland<br>Phone: +41 44 244 36 60<br>Email: clinicaltrials@cutiss.swiss |


| | | |
|---|---|---|
| **Monitors:** | Julius Clinical<br>Broederplein 41-43<br>3703 CD Zeist,<br>The Netherlands<br>Phone: +31 (0)30 656 99 00<br>Fax: +31 30 656 99 90<br>Email: info@juliusclinical.com | Sintesi Research S.r.l.<br>C.so di P.ta Romana, 132<br>20122 Milano, Italy<br>Phone +39 02 87 35 121<br>Fax : +39 02 97 37 43 01<br>Email:<br>p.desimoni@sintesiresearch.com |

# TABLE OF CONTENTS


**SPONSOR PROTOCOL APPROVAL ..... 7**

**COORDINATING INVESTIGATOR AGREEMENT ..... 8**

**PRINCIPAL INVESTIGATOR AGREEMENT ..... 9**

**STUDY ADMINISTRATIVE STRUCTURE ..... 10**

**ABBREVIATIONS AND DEFINITIONS ..... 12**

**PROTOCOL SYNOPSIS ..... 14**

**1. INTRODUCTION ..... 20**

1.1 BACKGROUND AND RATIONALE ..... 20
*1.1.1 Non-Clinical Summary ..... 21*
*1.1.2 Clinical Summary ..... 21*
*1.1.3 Rationale for the Study Product ..... 21*
1.2 INVESTIGATIONAL MEDICINAL PRODUCT ..... 22
1.3 BENEFIT / RISK ASPECTS ..... 23
*1.3.1 Benefits of EHSG-KF ..... 23*
*1.3.2 Risks of EHSG-KF and Risk Mitigation Strategies ..... 24*
1.4 DOSE RATIONALE ..... 25
1.5 JUSTIFICATION OF CHOICE OF STUDY POPULATION ..... 26
1.6 CONCLUSION ..... 26

**2. STUDY OBJECTIVES ..... 27**

2.1 PRIMARY OBJECTIVE ..... 27
2.2 FIRST SECONDARY OBJECTIVE ..... 27
2.3 SECONDARY OBJECTIVE ..... 27

**3. INVESTIGATIONAL MEDICINAL PRODUCT INFORMATION ..... 28**

3.1 PRODUCTION OF IMP ..... 28
3.2 PACKAGING, LABELLING, SUPPLY, ACCOUNTABILITY, DESTRUCTION ..... 28
*3.2.1 Packaging ..... 28*
*3.2.2 Transport ..... 28*
*3.2.3 Labelling ..... 28*
*3.2.4 Supply ..... 29*
*3.2.5 Storage Conditions ..... 29*
*3.2.6 Study Product Accountability ..... 29*
*3.2.7 Return or Destruction of Study Product ..... 29*
*3.2.8 Out of Specification Product ..... 29*
3.3 EXPERIMENTAL INTERVENTION ..... 29
3.4 CONTROL INTERVENTION (CURRENT GOLD STANDARD) ..... 30
3.5 RANDOMISATION ..... 30
3.6 COMPLIANCE WITH STUDY INTERVENTION ..... 31
3.7 CONCOMITANT MEDICATION ..... 32
*3.7.1 Prohibited Concomitant Medication ..... 32*
*3.7.2 Allowed Concomitant Medication ..... 32*
*3.7.3 Concomitant Medication to be used with Caution ..... 32*
*3.7.4 Allowed Dressing ..... 32*

**4. SELECTION OF STUDY POPULATION ..... 33**

4.1 INCLUSION CRITERIA ..... 33
4.2 EXCLUSION CRITERIA ..... 33

4.2.1 *Contraception Methods* ... *33*
4.3 CRITERIA FOR WITHDRAWAL / DISCONTINUATION OF PATIENTS ... 34
4.3.1 *Early Withdrawal of Patients* ... *34*
4.3.2 *Criteria for Early Withdrawal of Patients by Investigator* ... *34*
4.3.3 *Replacement of Patients* ... *35*
4.4 DATA COLLECTION AND FOLLOW-UP FOR WITHDRAWN PATIENTS ... 35

**5. STUDY DESIGN AND COURSE OF THE STUDY ... 36**

5.1 STUDY CENTRES ... 36
5.2 STUDY DESIGN ... 36
5.3 STUDY PROCEDURES ... 36
5.4 SELECTION OF STUDY AREAS ... 37
5.5 END OF STUDY ... 37

**6. STUDY ENDPOINTS ... 38**

6.1 PRIMARY ENDPOINT ... 38
6.2 FIRST SECONDARY ENDPOINT ... 38
6.3 SECONDARY ENDPOINTS ... 38
6.4 EXPLORATORY ENDPOINTS ... 38
6.5 DURATION OF PATIENT PARTICIPATION ... 39

**7. STUDY ASSESSMENT ... 41**

7.1 STUDY FLOW CHART / TABLE OF STUDY PROCEDURES AND ASSESSMENTS ... 41
7.2 PROCEDURES AT EACH VISIT ... 42
7.2.1 *Overview of Study Visits* ... *42*
7.2.2 *Visit 1 - Pre-treatment phase (Screening)* ... *42*
7.2.3 *Visit 2 - Pre-treatment phase (Biopsy)* ... *43*
7.2.4 *Visit 3 - Treatment phase (Grafting, day 0)* ... *43*
7.2.5 *Visit 4 - Treatment phase (First Graft Check, day 6-10)* ... *44*
7.2.6 *Visit 5 - Treatment phase (Second Graft Check, day 21 ± 2)* ... *44*
7.2.7 *Visit 6 - Treatment phase (Third Graft Check, day 28 ± 3)* ... *44*
7.2.8 *Visit 7 - Post-treatment phase (Follow-up, day 60 ± 3)* ... *45*
7.2.9 *Visit 8 - Post-treatment phase (Follow-up, day 90 ± 5)* ... *45*
7.2.10 *Visit 9 - Post-treatment phase (Safety-Follow-up, 6 months ± 10 days)* ... *45*
7.2.11 *Visit 10 - Post-treatment phase (Safety-Follow-up, 1 year ± 30 days)* ... *45*
7.2.12 *Visit 11 - Post-treatment phase (Safety-Follow-up, 2 years ± 30 days )* ... *46*
7.2.13 *Visit 12 - Post-treatment phase (Safety-Follow-up, 3 years ± 30 days)* ... *46*
7.3 GENERAL DESCRIPTION OF STUDY PROCEDURES ... 47
7.3.1 *Recruitment Procedure* ... *47*
7.3.2 *Skin Biopsy for IMP Manufacturing* ... *47*
7.3.3 *Laboratory Analyses* ... *48*
7.3.4 *Clinical Evaluation of Signs of Infection* ... *48*
7.3.5 *Grafting* ... *49*
7.3.6 *Facultative Biopsies at Visit 3 (during Grafting)* ... *51*
7.3.7 *Optional Biopsies at Visit 8 and 10 (~90 days and ~1 year after Grafting)* ... *51*
7.3.8 *Photography* ... *51*
7.3.9 *Study Visits Outside Time Window* ... *52*
7.3.10 *Unscheduled Visits* ... *52*

**8. ASSESSMENTS OF ENDPOINTS ... 53**

8.1 PRIMARY ENDPOINT ... 53
8.1.1 *Skin Amplification* ... *53*
8.2 SECONDARY AND EXPLORATORY ENDPOINT ... 54
8.2.1 *Epithelialization %, Time to Complete Epithelialization, and Incidence of Wound Closure* *54*

8.2.2 Graft Take ... 54
8.2.3 Scar Quality ... 54
8.2.4 POSAS ... 54
8.2.5 Cutometer® ... 54
8.2.6 Colour ... 55
8.2.7 Clinical Signs of Infection ... 56
8.2.8 Number and Type of Adverse Events ... 56
8.2.9 Quality of Life Assessment ... 56
8.2.10 Healthcare Resource Utilization ... 57
8.2.11 Additional Safety Measures ... 58
8.2.12 Growth measurement ... 58
8.2.13 Assessments in Patients Who Prematurely Stop the Study ... 58

9. SAFETY ... 59

9.1 DEFINITIONS OF (SERIOUS) ADVERSE EVENTS AND OTHER SAFETY RELATED EVENTS ... 59
9.1.1 Adverse Events ... 59
9.1.2 Serious Adverse Events ... 59
9.1.3 Adverse Reaction and Suspected Adverse Reaction ... 60
9.1.4 Unexpected Adverse Drug Reaction ... 60
9.1.5 Suspected Unexpected Serious Adverse Reaction (SUSAR) ... 60
9.1.6 Safety Signals ... 60
9.2 RECORDING OF ADVERSE EVENTS AND OTHER SAFETY RELATED EVENTS ... 60
9.2.1 Recording of Intermittent Adverse Events ... 61
9.3 ASSESSMENT OF ADVERSE EVENTS AND OTHER SAFETY RELATED EVENTS ... 61
9.3.1 Relationship Categorization ... 61
9.3.2 Severity Grading ... 62
9.3.3 Expectedness ... 63
9.4 REPORTING OF SERIOUS ADVERSE EVENTS AND OTHER SAFETY RELATED EVENTS ... 63
9.4.1 Reporting of SAEs ... 63
9.4.2 Reporting of Safety Signals ... 64
9.4.3 IRB / IECs Safety Reporting by the PI ... 64
9.4.4 Regulatory Authorities Safety Reporting by the Sponsor ... 64
9.5 REPORTING AND HANDLING OF PREGNANCIES ... 64
9.6 FOLLOW-UP OF (SERIOUS) ADVERSE EVENTS ... 65
9.7 ANTICIPATED ADVERSE EVENTS ... 65

10. STATISTICAL ANALYSES ... 66

10.1 ANALYSIS PLAN ... 66
10.1.1 Final analysis ... 66
10.1.2 Long-term follow-up analysis ... 66
10.1.3 Interim analysis ... 66
10.2 DETERMINATION OF SAMPLE SIZE ... 66
10.3 PLANNED ANALYSES ... 67
10.3.1 Analysis Populations ... 67
10.3.2 Primary Analyses ... 68
10.3.3 Secondary Analyses ... 68
10.3.4 First Secondary Analyses – Epithelialization at Visit 8 ... 68
10.3.5 Secondary Analyses - Clinical and Microbiological Signs of Infection at Visits 4 and 5: 68
10.3.6 Secondary Analyses – Scar Quality (Elasticity of the Study Areas at Visit 10 (Cutometer®)) ... 69
10.3.7 Secondary Analyses – Scar Quality (General Quality at Visit 10 (POSAS)) ... 69
10.3.8 Secondary Analyses – AE Reporting (during full study duration) ... 69
10.3.9 Secondary Analyses – Epithelialization at Visit 6 ... 69
10.3.10 Exploratory Analyses ... 69

10.3.11 *Exploratory Analyses - Graft Take at Visit 4* ... 69
10.3.12 *Exploratory Analyses - Epithelialization at Visits 5, 7, 9* ... 69
10.3.13 *Exploratory Analyses – Clinical Signs of Infection at Visit 6* ... 70
10.3.14 *Exploratory Analyses – Incidence of Wound Closure at Visits 6, 7, and 8* ... 70
10.3.15 *Exploratory Analyses – Scar Quality, Elasticity of the Study Areas at Visits 8, 9, 11 and 12 (Cutometer®)* ... 70
10.3.16 *Exploratory Analyses – General Scar Quality at Visits 8, 9, 11 and 12 (POSAS)* ... 70
10.3.17 *Exploratory Analyses – Scar Erythema and Pigmentation at Visits 8, 9, 10 11 and 12 (DSM Colormeter®)* ... 70
10.3.18 *Exploratory Analyses – Quality of Life Assessment at Visits 8, 9, 10, 11, 12* ... 70
10.3.19 *Exploratory Analyses – Healthcare Resource Utilization* ... 70

**11. DATA QUALITY ASSURANCE AND CONTROL ... 72**

11.1 DATA HANDLING AND RECORD KEEPING / ARCHIVE ... 72
11.1.1 *Case Report Forms* ... 72
11.1.2 *Specification of Source Documents* ... 73
11.1.3 *Record Keeping / Archiving* ... 73
11.1.4 *Retention of Records* ... 74
11.2 DATA MANAGEMENT ... 74
11.2.1 *Data Management System* ... 74
11.2.2 *Data Security, Access, and Backup* ... 74
11.2.3 *Data Exports / Transfer for Analysis* ... 75
11.2.4 *Electronic and Central Data Validation* ... 75
11.3 STAFF TRAINING ... 75
11.4 MONITORING ... 75
11.5 INDEPENDENT DATA SAFETY MONITORING BOARD ... 75
11.6 AUDITS AND INSPECTIONS ... 76
11.7 PROCESSING OF PERSONAL DATA ... 76
11.7.1 *Study Patients' Personal Data* ... 76
11.7.2 *Study Staff* ... 77
11.8 CLINICAL LABORATORY EVALUATION ... 78
11.9 VITAL SIGNS ... 78

**12. ETHICAL AND REGULATORY ASPECTS ... 79**

12.1 STUDY REGISTRATION ... 79
12.2 STUDY CATEGORIZATION ... 79
12.3 INDEPENDENT ETHICS COMMITTEE (IEC) ... 79
12.4 REGULATORY AUTHORITY (RA) ... 80
12.5 PATIENT PRIVACY AND CONFIDENTIALITY ... 80
12.6 NON-DISCLOSURE ... 80
12.7 DECLARATION OF INTEREST ... 81
12.7.1 *Oversight Committee (OC)* ... 81
12.8 PATIENT INFORMATION AND INFORMED CONSENT ... 81
12.9 STUDY SITE DISCONTINUATION ... 83
12.10 MODIFICATIONS OF THE PROTOCOL ... 83
12.11 DEVIATIONS FROM STUDY PROTOCOL ... 83
12.12 GOOD CLINICAL PRACTICE (GCP) COMPLIANCE ... 84
12.13 STUDY COMPLETION ... 84

**13. PUBLICATION AND DISSEMINATION POLICY ... 85**

**14. FUNDING AND SUPPORT ... 86**

**15. INSURANCE ... 87**

**16. APPENDIX ... 88**

16.1 APPENDIX 1 - STUDY FLOW SHEET ..... 88
16.2 APPENDIX 2 – ADDENDUM 1 FOR SUB-STUDY ..... 89

**17. REFERENCES ..... 99**

The experimental protocol for this study has been designed in accordance with the general ethical principles outlined in the Declaration of Helsinki. The review of this protocol by the Institutional Review Board and the performance of all aspects of the study, including the methods used for obtaining informed consent, must also be in accordance with principles enunciated in the declaration, the ICH E6 (R2) guidelines of Good Clinical Practice (GCP), 21 CFR 21.50 Protection of Human Patients and 21 CFR 21.56 Institutional Review Boards, and all applicable regulatory authority (RA) requirements.

## Sponsor Protocol Approval

Protocol title:
"A Phase IIb, prospective, intra-patient randomised controlled, multicentre study to evaluate the safety and efficacy of an autologous bio-engineered dermo-epidermal skin substitute (EHSG-KF) for the treatment of partial deep dermal and full-thickness burns in adults and adolescents in comparison to autologous split-thickness skin grafts (STSG)"

With a sub-study at the sites a) Azienda Ospedaliera di Rilievo Nazionale Antonio Cardarelli (Italy), b) Santobono Napoli (Italy), and c) Azienda Ospedale Università Padova (Italy)
SUBSTUDY TO PROTOCOL TBRU-dS-BA-PIIb

Title: An open-label, prospective, non-randomised, non-controlled study to evaluate the survival of the adult and adolescent patients with deep partial and full-thickness burns in a life threatening situation treated with EHSG-KF, and to evaluate the safety and efficacy of EHSG-KF

Study Identifiers:
TBRU-dS-BA-PIIb

Sponsor Approval:
The Sponsor has approved the clinical study protocol

TBRU-dS-BA-PIIb, version 12, dated 2022-10-13

and confirms hereby to conduct the study according to the protocol, the current version of the World Medical Association Declaration of Helsinki, ICH-GCP guidelines and the local legally applicable requirements.

CUTISS AG
Grabenstrasse 11
8952 Schlieren
Switzerland

| Name, Function | Place, Date | Signature |
| --- | --- | --- |
| Name, Function | Place, Date | Signature |

## Coordinating Investigator Agreement

Protocol title:
“A Phase IIb, prospective, intra-patient randomised controlled, multicentre study to evaluate the safety and efficacy of an autologous bio-engineered dermo-epidermal skin substitute (EHSG-KF) for the treatment of partial deep dermal and full-thickness burns in adults and adolescents in comparison to autologous split-thickness skin grafts (STSG)”

With a sub-study at the sites a) Azienda Ospedaliera di Rilievo Nazionale Antonio Cardarelli (Italy), b) Santobono Napoli (Italy), and c) Azienda Ospedale Università Padova (Italy)
SUBSTUDY TO PROTOCOL TBRU-dS-BA-PIIb

Title: An open-label, prospective, non-randomised, non-controlled study to evaluate the survival of the adult and adolescent patients with deep partial and full-thickness burns in a life threatening situation treated with EHSG-KF, and to evaluate the safety and efficacy of EHSG-KF

Study Identifiers:
TBRU-dS-BA-PIIb

Confidentiality statement:
This and related documents from the Sponsor contain privileged information that is confidential and may not be disclosed unless such disclosure is required by federal laws or regulations. In any event, persons to whom the information is disclosed must be informed that it is privileged and/or confidential and may not be further disclosed by them. Information from this study may not be reproduced in any form without the written permission of the Sponsor.

Protocol Compliance:
The signature of the Coordinating Investigator below constitutes his/her agreement to comply with the contents of this clinical study protocol

TBRU-dS-BA-PIIb, version 12, dated 2022-10-13

and to conduct this study according to Good Clinical Practices (GCP) and applicable requirements.

Coordinating Investigator:

Prof. Dr. med. Clemens Schiestl
Director, Paediatric Burn Centre
Department of Surgery
University Children’s Hospital Zurich

Place, Date ____________________ Signature ____________________

# Principal Investigator Agreement

For every study site, the Principal Investigator of the study site is requested to complete and sign this agreement page.

Protocol title:
“A Phase IIb, prospective, intra-patient randomised controlled, multicentre study to evaluate the safety and efficacy of an autologous bio-engineered dermo-epidermal skin substitute (EHSG-KF) for the treatment of partial deep dermal and full-thickness burns in adults and adolescents in comparison to autologous split-thickness skin grafts (STSG)”

With a sub-study at the sites a) Azienda Ospedaliera di Rilievo Nazionale Antonio Cardarelli (Italy), b) Santobono Napoli (Italy), and c) Azienda Ospedale Università Padova (Italy)
SUBSTUDY TO PROTOCOL TBRU-dS-BA-PIIb

Title: An open-label, prospective, non-randomised, non-controlled study to evaluate the survival of the adult and adolescent patients with deep partial and full-thickness burns in a life threatening situation treated with EHSG-KF, and to evaluate the safety and efficacy of EHSG-KF

Study Identifiers:
TBRU-dS-BA-PIIb

Confidentiality statement:
This and related documents from the Sponsor contain privileged information that is confidential and may not be disclosed unless such disclosure is required by federal laws or regulations. In any event, persons to whom the information is disclosed must be informed that it is privileged and/or confidential and may not be further disclosed by them. Information from this study may not be reproduced in any form without the written permission of the Sponsor.

Protocol Compliance:
The signature of the Principal Investigator below constitutes his/her agreement to comply with the contents of this clinical study protocol

TBRU-dS-BA-PIIb, version 12, dated 2022-10-13

and to conduct this study according to Good Clinical Practices (GCP) and applicable requirements.

| | |
|---|---|
| Study Site Name | Name Principal Investigator |
| Place, Date | Signature Principal Investigator |

# Study Administrative Structure

| Structure | Details |
|---|---|
| **Sponsor** | CUTISS AG<br>Grabenstrasse 11<br>8952 Schlieren, Switzerland<br>Phone: +41 44 244 36 60<br>Email: clinicaltrials@cutiss.swiss |
| **Coordinating Investigator** | Prof. Dr. med. Clemens Schiestl<br>Director, Paediatric Burn Centre<br>Department of Surgery<br>University Children's Hospital Zurich<br>Steinwiesstrasse 75<br>8032 Zurich, Switzerland<br>Phone: +41 44 266 74 13<br>Email: Clemens.Schiestl@kispi.uzh.ch |
| **Statistician** | Jean-Christophe Lemarie<br>Director of Statistics<br>EFFI-STAT<br>22, rue du Pont Neuf<br>75001 Paris, France<br>Phone: +33 15 534 96 00<br>Email: jean-christophe.lemarie@effi-stat.com |
| | Julius Clinical<br>Broederplein 41-43<br>3703 CD Zeist, The Netherlands<br>Phone: +31 (0)30 656 99 00<br>Fax Number: +31 30 656 99 90<br>Email: info@juliusclinical.com |
| **Monitoring Institution** | Julius Clinical<br>Broederplein 41-43<br>3703 CD Zeist, The Netherlands<br>Phone: +31 (0)30 656 99 00<br>Fax: +31 30 656 99 90<br>Email: tbru.safety@juliusclinical.com |
| | Sintesi Research S.r.l.<br>C.so di P.ta Romana, 132<br>20122 Milano, Italy<br>Phone +39 02 87 35 121<br>Fax : +39 02 97 37 43 01<br>Email: p.desimoni@sintesiresearch.com |
| **Independent Data Safety Monitoring Board** | Prof. Dr. Christoph Berger<br>Paediatrician, Infectious Diseases<br>University Children's Hospital Zurich<br>Department of Paediatrics<br>Steinwiesstrasse 75<br>8032 Zurich, Switzerland<br>Phone: +41 44 266 72 50<br>Email: christoph.berger@kispi.uzh.ch |

| | |
|---|---|
| | Dr. med. Abdul R. Jandali<br>Director, Hand and Plastic Surgery<br>Kantonsspital Winterthur<br>Brauerstrasse 15<br>Postfach 834<br>8401 Winterthur, Switzerland<br>Phone: +41 52 266 24 08<br>Email: abed.jandali@ksw.ch |
| | Prof. Dr. Med. Holger Bannasch<br>ÄD Klinik für Plastische-, Hand-, und Ästhetische Chirurgie Kliniken Donaueschingen<br>Sonnhaldenstr. 2<br>78166 Donaueschingen, Germany<br>Phone: +49 771 88-0<br>Email: holger.bannasch@sbk-vs.de |
| **Oversight Committee** | Univ. Prof. Dr. Med. Peter M. Vogt<br>Direktor<br>Medizinische Hochschule Hannover<br>Schwerbrandverletztenzentrum der Klinik für Plastische-, Hand und Wiederherstellungschirurgie<br>Carl-Neuberg-Str. 1<br>30625 Hannover, Germany<br>Phone: +49 511 532 – 0<br>Fax: +49 511 532 – 8890<br>Email: phw@mh-hannover.de |
| | Prof. Dr. Med. Hans-Oliver Rennekampff<br>Rhein-Maas Klinikum<br>Klinik für Plastische Chirurgie, Hand- und Verbrennungschirurgie<br>Mauerfeldchen 25<br>52146 Würselen, Germany<br>Phone: +49 2405 62 33 13<br>Email: hans-oliver.rennekampff@rheinmaasklinikum.de |
| | Prof. Dr. Med. Matthias Baumgartner<br>Abteilung für Stoffwechselkrankheiten Universitäts-Kinderspital Zurich<br>Steinwiesstrasse 75<br>8032 Zurich, Switzerland<br>Phone: +41 44 266 77 22<br>Email: matthias.baumgartner@kispi.uzh.ch |

CUTISS personalized skin

# Abbreviations and Definitions

| Abbreviation | Full text |
| --- | --- |
| AE | Adverse Event |
| AR | Adverse Reaction |
| ATMP | Advanced Therapy Medicinal Product |
| BP | Blood Pressure |
| BSA | Body Surface Area |
| BSHS-B | Burn Specific Health Scale – Brief Questionnaire |
| CEA | Cultured Epithelial Autografts |
| ClinO | Clinical Trials Ordinance |
| CrP | C-reactive Protein |
| CS | Clinical Success |
| CSR | Clinical Study Report |
| CTCAE | Common Terminology Criteria for Adverse Events |
| denovoSkin | Synonym for EHSG-KF |
| DIBD | Development International Birth Date |
| DSUR | Development Safety Update Report |
| eCRF | Electronic Case Report Form |
| EHSG-KF | Engineered hydrogel skin graft with incorporated keratinocytes and fibroblasts. Synonym denovoSkin |
| EMA | European Medicines Agency |
| EQ-5D | EuroQol 5 Dimensions Questionnaire for patients >18years |
| EQ-5DY | EuroQol 5 Dimensions Questionnaire for patients <18years |
| EU | European Union |
| FDA | Food and Drug Agency |
| GCP | Good Clinical Practice |
| GDPR | General Data Protection Regulation |
| GMP | Good Manufacturing Practice |
| H0 | Null hypothesis |
| H1 | Alternative hypothesis |
| HBV | Hepatitis B Virus |
| HCV | Hepatitis C Virus |
| HIV | Human Immunodeficiency Virus |
| IB | Investigator's Brochure |
| IC | Informed Consent |
| ICF | Informed Consent Form |
| ICH | International Conference of Harmonization |
| IDSMB | Independent Data Safety Monitoring Board |
| IEC | Independent Ethics Committee |
| IMP | Investigational Medicinal Product |
| IMPD | Investigational Medicinal Product Dossier |
| INR | International Normalized Ratio |
| IRB | Institutional Review Board |
| ISF | Investigator Site File |

| | |
|---|---|
| ITT | Intention to Treat |
| LLN | Lower Limit of Normal |
| MedDRA | Medical Dictionary for Regulatory Activities |
| N/A | Not applicable |
| PedsQL | Paediatric Quality of Life Questionnaire |
| PI | Principal Investigator |
| PIS | Patient Information Sheet |
| POSAS | Patient and Observer Scar Assessment Scale |
| premenopausal | Defined as: <2 years after last menstruation and not surgically sterile |
| PTT | Partial Thromboplastin Time |
| OC | Oversight Committee |
| OOS | Out of Specification |
| QOL | Quality of Life |
| RA | Regulatory Authority (e.g. Swissmedic) |
| SAE | Serious Adverse Event |
| SAP | Statistical Analysis Plan |
| SD | Standard Deviation |
| SDV | Source Data Verification |
| SmPC | Summary of Product Characteristics |
| SNCTP | Swiss National Clinical Trial Portal |
| SOP | Standard Operating Procedure |
| STSG | Split-Thickness Skin Graft |
| SUSAR | Suspected Unexpected Serious Adverse Reaction |
| TBSA | Total Body Surface Area |
| TESS | Tissue Engineered Skin Substitute |
| TMF | Trial Master File |
| TPP | Techno Plastic Products |
| ULN | Upper Limit of Normal |
| WI | Working Instruction |

## Protocol Synopsis

| | |
|---|---|
| Study Title: | A Phase IIb, prospective, intra-patient randomised controlled, multicentre study to evaluate the safety and efficacy of an autologous bio-engineered dermo-epidermal skin substitute (EHSG-KF) for the treatment of partial deep dermal and full-thickness burns in adults and adolescents in comparison to autologous split-thickness skin grafts (STSG) |
| Short Title/Study ID: | TBRU-dS-BA-PIIb |
| Protocol Version and Date: | Version 12, 2022-10-13 |
| Study Category: | Clinical trial with IMP Category C according to ClinO (Swiss law) |
| Clinical Phase: | Phase IIb |
| Background and Rationale: | The management of severe burns remains a significant challenge. The current gold standard, excision and coverage with meshed STSG, is limited by both donor site availability and the risk of disfiguring and functionally debilitating scars. The introduction of cultured epithelial autografts (CEA) has helped address donor site limitations; however, thirty years since its introduction, despite tremendous research efforts, CEA continues to yield unacceptable results when used independently for the coverage of deep burns. The role of CEA in contemporary burn care is, therefore, largely adjunctive, as is the case with other keratinocyte replacement techniques, such as keratinocyte spray.<br><br>The clinical introduction of now widely used dermal regeneration templates (e.g. IntegraDRT® and Matriderm®) has pushed the frontiers further, with potential for improved aesthetic and functional results. However, such templates still require coverage with an overlying skin graft. The evolution of an autologous tissue-engineered skin substitute, such as EHSG-KF, that can be used as an alternative to a STSG, represents the next step towards achieving coverage of severe burns with limited donor sites, thereby offering a potentially lifesaving therapy.<br>EHSG-KF is a tissue-engineered autologous dermo-epidermal skin substitute for the treatment of partial deep dermal and full-thickness skin burns. The proposed phase IIb clinical trial aims to evaluate the safety and efficacy of EHSG-KF in adult patients with severe burns, when compared to meshed STSG, the current gold standard.<br><br>The present proposal is based on the successful completion of the phase I clinical trial in which EHSG-KF was safely applied as an autologous skin substitute for burns and for the reconstruction of burn scars and congenital nevi in children. There were no cases of infection, and graft take was acceptable. In addition, the healed EHSG-KF yielded high quality scars and excellent tissue elasticity. These findings further substantiate the preclinical animal and laboratory studies, supporting EHSG-KF as a viable skin substitute.<br>The proposed multicentre phase IIb clinical trial will target adult patients and adolescents with severe burns to elucidate the benefit of a tissue-engineered autologous skin substitute for the patient group with the highest mortality rates. Particular emphasis, apart from safety, will be placed on efficacy, including the ratio of covered surface area to harvested surface area and scar quality, in comparison to meshed STSG. If the results of this phase IIb study support the hypothesis that EHSG-KF provides successful coverage of acute burn defects in adult patients and adolescents with severe burns, with greater skin amplification than conventional meshed STSGs, the goal of creating an autologous bio-engineered skin substitute that serves as a favourable alternative to STSGs will be realized. |

| | |
|---|---|
| | EHSG-KF carries the potential to be a life-saving therapy for patients with severe burns, and we believe it will also improve quality of life for our patients. |
| Objectives: | To evaluate the efficacy and safety of EHSG-KF in comparison to meshed STSG in adults and adolescents with partial deep dermal and full-thickness burns.<br><br><u>Primary Objective</u><br>To evaluate the efficacy of EHSG-KF in comparison to meshed STSG based on:<br>• Ratio of covered surface area to biopsy site/donor site surface area 4 weeks post grafting<br><br><u>First Secondary Objective</u><br>• % Epithelialization at 3 months post grafting<br><br><u>Secondary Objectives</u><br>To evaluate the safety and efficacy of EHSG-KF in comparison to meshed STSG based on the assessment of:<br>• Infection<br>• Scar quality:<br>    ○ Cutometer® 3, 6, 12, 24 and 36 months post grafting<br>    ○ DSM ColorMeter® 3, 6, 12, 24 and 36 months post grafting<br>    ○ POSAS-questionnaire 3, 6, 12, 24 and 36 months post grafting<br>• Graft take at 6-10 days post grafting<br>• %Epithelialization (to estimate 'time to complete epithelialization') at 3 and 4 weeks, and 2 and 6 months post grafting<br>• Incidence of wound closure at 4, 8, and 12 weeks post grafting<br>• Growth (% change in surface area, $cm^2$, between 1 and 3 years post grafting)<br>• Assessment and reporting of all observed adverse events<br>• QOL assessment (EQ-5D and BSHS-B for patients ≥18 years; EQ-5DY and PedsQL for patients <18 years)<br>• Healthcare resource utilization (direct and indirect healthcare costs, this questionnaire will not be handed out to patients) |
| Endpoints: | <u>Primary Endpoint</u><br>Efficacy evaluation, as a comparison between the EHSG-KF and control sites, based on:<br>• Ratio of covered surface area to biopsy site/donor site surface area at:<br>    ○ visit 6 (28 ± 3 days post grafting)<br><br>First Secondary Endpoint<br>• % Epithelialization at:<br>    ○ visit 8 (90 ± 5 days post grafting)<br><br><u>Secondary Endpoints</u><br>Safety and efficacy evaluation, as a comparison between the EHSG-KF and control sites, based on:<br>• Main secondary safety endpoint:<br>Clinical and microbiologic signs of infection at<br>    ○ visit 4 (6-10 days post grafting)<br>    ○ visit 5 (21 ± 2 days post grafting)<br>• Main secondary efficacy endpoints:<br>Scar quality at the study areas<br>    ○ Assessment of elasticity of the study areas using the Cutometer® at visit 10 (1 year ± 30 days post grafting) |

  - Assessment of general scar quality at the study areas using the POSAS, a reliable and validated scar assessment tool, at: visit 10 (1 year ± 30 days post grafting)
- Other secondary safety endpoint:
  Assessment and reporting of all observed adverse events will be carried out for the full duration of the study from visit 2 on.
- Other secondary efficacy endpoint:
  Epithelialization at:
  - visit 6 (28 ± 3 days post grafting)

<u>Exploratory Endpoints</u>

- Graft take of the study areas assessed in a standardized manner as percentage of the whole grafted area during the first dressing change at:
  - visit 4 (6-10 days post grafting)
- % Epithelialization (to estimate 'time to complete epithelialization') at:
  - visit 5 (21 ± 2 days post grafting)
  - visit 7 (60 ± 3 days post grafting)
  - visit 9 (6 months ± 10 days post grafting)
- Clinical and microbiologic signs of infection at:
  - visit 6 (28 ± 3 days post grafting)
- Incidence of wound closure at:
  - visit 6 (28 ± 3 days post grafting)
  - visit 7 (60 ± 3 days post grafting)
  - visit 8 (90 ± 5 days post grafting)
- Assessment of elasticity of the study areas using the Cutometer® at:
  - visit 8 (90 ± 5 days post grafting)
  - visit 9 (6 months ± 10 days post grafting)
  - visit 11 (2 years ± 30 days post grafting)
  - visit 12 (3 years ± 30 days post grafting)
- Assessment of general scar quality at the study areas using the POSAS, a reliable and validated scar assessment tool, at:
  - visit 8 (90 ± 5 days post grafting)
  - visit 9 (6 months ± 10 days post grafting)
  - visit 11 (2 years ± 30 days post grafting)
  - visit 12 (3 years ± 30 days post grafting)
- Assessment of colour (erythema and pigmentation) of the study areas using the DSM ColorMeter® at:
  - visit 8 (90 ± 5 days post grafting)
  - visit 9 (6 months ± 10 days post grafting)
  - visit 10 (1 year ± 30 days post grafting)
  - visit 11 (2 years ± 30 days post grafting)
  - visit 12 (3 years ± 30 days post grafting)
- Growth (% change in surface area, $cm^2$, between 1 year (visit 10) and 3 years (visit 12) post grafting)
- QOL assessment (EQ-5D and BSHS-B for patients ≥18 years; EQ-5DY and PedsQL for patients <18 years) at:
  - visit 8 (90 ± 5 days post grafting)
  - visit 9 (6 months ± 10 days post grafting)
  - visit 10 (1 year ± 30 days post grafting)
  - visit 11 (2 years ± 30 days post grafting)
  - visit 12 (3 years ± 30 days post grafting)
- Healthcare resource utilization (direct and indirect healthcare costs)
  - visit 9 (6 months ± 10 days post grafting)
  - visit 10 (1 year ± 30 days post grafting)
  - visit 11 (2 years ± 30 days post grafting)

| | |
|---|---|
| Study Design: | Open label, intra-patient randomised controlled, prospective, multicentre phase IIb clinical trial<br><br><u>Intra-patient randomisation:</u><br>Two sites, A and B, each an area of 45-90 $cm^2$ are selected. Each site is covered either with a meshed STSG, or EHSG-KF, and the type of graft for each site has been determined in advance.<br><br>The endpoint measures described above, including ratio of covered surface area to harvested surface area and presence of infection, will be determined for each site, and comparisons made. |
| Inclusion/Exclusion Criteria: | <u>Inclusion criteria:</u><br>• Age: ≥12 years of age<br>• Deep partial thickness and/or full-thickness burns requiring surgical wound coverage<br>• Expected that ≥90 $cm^2$ of wound (not counting the head and neck area for study patients in The Netherlands) will remain open at 4 weeks post burn despite proceeding with treatment in accordance with the standard of care. >20% TBSA burns can be taken as guideline, but TBSA is not an inclusion criterion.<br>• Signed informed consent from the patient or the parents/legally authorized representative.<br><br><u>Exclusion criteria:</u><br>• Patients tested positive for HBV, HCV, syphilis or HIV<br>• Patients with known underlying or concomitant medical conditions that may interfere with normal wound healing (e.g. systemic skin and connective tissue diseases, any kind of congenital defect of metabolism including insulin-dependent diabetes mellitus, Cushing syndrome or disease, scurvy, chronic hypothyroidism, congenital or acquired immunosuppressive condition, chronic renal failure, or chronic hepatic dysfunction (Child-Pugh class B or C), severe malnutrition, or other concomitant illness which, in the opinion of the Investigator, has the potential to significantly delay wound healing)<br>• Severe drug and alcohol abuse<br>• Pre-existing coagulation disorders as defined by INR outside its normal value, PTT >ULN and fibrinogen <LLN prior to the current hospital admission and / or at the Investigator's discretion<br>• Patients with known allergies to amphotericin B, gentamicin, penicillin, streptomycin, or bovine collagen<br>• Previous enrolment of the patient into the current phase II study<br>• Participation of the patient in another study with conflicting endpoints within 30 days preceding and during the present study<br>• Patients expected not to comply with the study protocol (including patients with severe cognitive dysfunction/impairment and severe psychiatric disorders)<br>• Pregnant or breast feeding females<br>• Intention to become pregnant during the clinical course of the study (12 months)<br>• Suspicion of non-accidental injury<br>• Wounds in the head and neck area as study target area (only applicable for study patients in The Netherlands)<br>• Enrolment of the Investigator, his/her family members, employees, and other dependent persons |

| | |
|---|---|
| Study Product/ Intervention: | Product:<br>EHSG-KF is an autologous tissue-engineered dermo-epidermal skin substitute based on a collagen type I hydrogel. The size per graft is 45 ± 4 $cm^2$ and the thickness is 0.5 - 2 mm.<br><br>Intervention:<br>Grafting of the wound bed (=experimental area) with 1 to 2 grafts of EHSG-KF |
| Control Intervention: | Product:<br>Autologous split-thickness skin graft (STSG) meshed at a ratio of 3:1<br><br>Intervention:<br>Grafting of the control wound bed (=control area) with meshed STSG, whereby size of control area=size of experimental area |
| Number of Patients: | 12 evaluable patients that could be inflated to 15 patients due to potential drop-outs |
| Study Duration/Schedule: | Clinical study (12 months):<br>• First-Patient-In: Q1 2018<br>• Last-Patient-Out: Q4 2023<br><br>Long-term follow-up (2 years):<br>• First-Patient-In: Q1 2021<br>• Last-Patient-Out: Q4 2025 |
| Study Procedure | Invasive procedures:<br>• Biopsy Nr. 1: mandatory biopsy, required for EHSG-KF production<br>• Biopsy Nr. 2-5: facultative biopsies before and after debridement for histological examination of the wound bed<br>• Blood samples for screening lab test<br>• Blood samples for routine lab test<br>• Grafting of EHSG-KF / STSG<br><br>Optional Invasive procedures:<br>• Optional biopsy Nr. 6-9: optional biopsy for routine histology and immune-histochemistry at visits 8 and 10<br><br>Non Invasive procedures:<br>• Physical examinations<br>• Elasticity assessment of the skin<br>• Colour assessment of the skin<br>• Photographic documentation of study areas<br>• Questionnaires<br>  ○ POSAS for all patients<br>  ○ EQ-5D and BSHS-B for patients ≥18 years<br>  ○ EQ-5DY and PedsQL for patients <18 years |
| Statistical Considerations: | • The primary endpoint (ratio of covered area to biopsy site / donor site surface area at 4 weeks post-grafting) will be analysed after logarithmic transformation by a one sample t-test at significance level of 5%<br>• Assuming a standard deviation of 1.00 for the paired difference between the 2 log (ratios), a sample size of 12 evaluable patients would be required in order to achieve a power of 80% to detect a treatment effect corresponding to an increase of 145% with EHSG-KF (i.e. to a difference of 0.90 on a logarithmic scale) when using a one-sample t-test at two-sided significance level of 5%. This calculated number may be inflated to 15 randomised patients in order to cover for possible drop-outs before the assessment of the primary endpoint. |

| | |
|---|---|
| | • A claim of study success will be made when the two-sided test passes the significance level of 0.05.<br>• All secondary endpoints will be tested in a strictly exploratory fashion only.<br>• The final analysis will be conducted once the last patient was treated with denovoSkin™ and completed the end of study visit at month 12 visit (V10). The long-term follow-up analysis will be conducted once all patients have completed the 2 follow-up visits, 2 and 3 years after denovoSkin™ application, V11 and V12 respectively. An interim analysis will be conducted on the month 3 data (V6) data from the first 12 patients who received denovoSkin™. |

# 1. Introduction

## 1.1 Background and Rationale

Despite tremendous prevention efforts, burn injuries remain a global health crisis. Eleven million people sought medical attention for burns in 2004, making them the fourth most common type of trauma after traffic accidents, falls, and interpersonal violence[1]. With advances in fluid resuscitation and critical care for burn patients, as well as surgical burn management, mortality rates for major burns have reduced significantly, and the cause of mortality has shifted from early burn shock to delayed multi-organ dysfunction syndrome and sepsis[2–4].

With increased survival of patients with extensive burns, clinicians and researchers face the challenge of providing definitive burn wound coverage in the setting of limited skin graft donor sites. The development of alternatives to autologous split-thickness skin grafts has thus become a cornerstone in burn research[5–11] and could prove to be life-saving and improve quality of life for patients with severe burns.

The current standard of care for the management of deep partial thickness and full-thickness burns is excision of the non-viable burned tissue and definitive coverage with autologous skin grafts[3,4,10,12,13]. In the developed world, temporary coverage is then typically achieved with cadaveric allograft or, less commonly, with biosynthetic skin substitutes until the patient's condition has stabilized and autologous split-thickness skin graft donor sites are available for harvest to provide definitive coverage[4,10]. Integra dermal regeneration templates® may also be used for coverage of excised burn defects providing a permanent dermal scaffold onto which a STSG can be grafted, if available, after 3 weeks[14,15].

Techniques such as conventional meshing and Meek micrografting allow expansion of autograft to increase the surface area of coverage in the setting of large burns with donor site limitations; however, when compared with non-meshed grafts, these techniques reduce the graft site healing rate, and decrease the area of dermal coverage, thereby compromising graft quality and leading to suboptimal long-term aesthetics and function[10]. Furthermore, autograft donor sites are painful, add to the wound healing burden of the patient, and can themselves lead to complications, such as unfavourable scars[10].

The introduction of cultured epithelial autografts (CEA) has helped address donor site limitations[16,17]; however, thirty years since its introduction, CEA continues to yield unacceptable results when used independently for the coverage of deep burns. The considerable problems associated with CEA include graft fragility, susceptibility to infection, poor graft take[11], propensity of healed grafts towards blistering and breakdown, and, finally and most importantly, unsatisfactory long-term results in terms of both functionality and aesthetics of the reconstituted skin[18–22]. The clinical introduction of now widely used dermal regeneration templates (e.g. IntegraDRT® and Matriderm®) has pushed the frontiers further, carrying the potential for improved aesthetic and functional results, yet such templates still require coverage with an overlying skin graft[14,15,23].

The evolution of an autologous tissue-engineered skin substitute, which can be used as an alternative to split-thickness skin grafts, represents the next step towards achieving coverage of severe burns with limited donor sites[5–8,11].

In the mid-1990s, the first cultured autologous dermo-epidermal skin substitute, developed by Boyce, was successfully applied clinically for 'compassionate use' in severe burns[24],and has more recently been investigated in a clinical trial setting[25]. Studies by Boyce et al., have confirmed that the ratio of defect coverage to skin harvested is substantially greater with their

bioengineered skin analogue[24–28]. However, twenty years later, this skin analogue has still not been brought to market and is not yet available for widespread use. Another bio-engineered dermo-epidermal skin substitute has been introduced in preclinical studies[29,30] and as a biologic dressing for refractory lower extremity ulcers[31].

The proposed phase IIb clinical trial aims to evaluate the safety and efficacy of EHSG-KF, a bioengineered autologous dermo-epidermal skin substitute, in adults and adolescents with severe burns, when compared to split-thickness skin grafts (STSG), the current gold standard.

### 1.1.1 Non-Clinical Summary

The proof-of-principle for EHSG-KF was demonstrated by the grafting of this bio-engineered skin analogue onto full-thickness defects, created surgically, in immuno-incompetent athymic rats and immuno-competent premium pigs[32,33]. Both animal models showed that the EHSG-KF readily heals full-thickness wounds by regenerating into a fully stratified epidermis supported by an organised and vascularised neodermis. The safety of EHSG-KF was demonstrated with local tolerance (immunotoxicity) and tumorigenicity studies carried out in immuno-competent house pigs and SCID-beige mice, respectively. For further details please refer to the Investigator's Brochure (IB).

### 1.1.2 Clinical Summary

The present proposal is based on the successful completion of the phase I clinical trial in which EHSG-KF was safely grafted as an autologous skin substitute for the reconstruction of burn scars and congenital nevi, and for the coverage of an acute burn, in 10 paediatric patients.
The primary endpoint was safety, as determined by the rates of local infection and graft take. There were no cases of infection, and graft take was acceptable. The mean graft take rate was 63.5% overall (median 78.0%, range 0-100%, SD 36.5%) and 70.6% (range 5-100%, SD 30.6%) when excluding one patient with 100% graft loss attributable to mechanical disruption of the graft post-operatively. Healed skin analogues have been stable thus far without blistering or breakdown.
There were three adverse events deemed to be possibly related to the study product/treatment. There were three severe adverse events (SAE), one that was deemed to be 'unrelated' to the study product/treatment and two that were deemed to be 'possibly related', but importantly all three of these SAEs are also expected complications of conventional grafting surgery. There were no suspected unexpected serious adverse reactions.
In addition, the healed EHSG-KF yielded high quality scars and excellent tissue elasticity, as supported by the POSAS scores. These findings further substantiate the preclinical animal and laboratory studies, supporting EHSG-KF as a viable skin substitute. The clinical phase of the study took place over two years (June 2014-June 2016), and long-term follow-up assessments were carried out annually for five years post grafting concluding in March 2021. For further details please refer to the IB.

### 1.1.3 Rationale for the Study Product

As noted above, currently available cell-based products are still plagued with considerable problems. We, therefore, embarked on the "skingineering" enterprise in order to develop an autologous dermo-epidermal skin analogue in the laboratory by means of bioengineering[6,9].

Over 15 years of laboratory research have been invested in the project of engineering an anatomically near-normal skin analogue. Professor Reichmann et al. succeeded in growing a hydrogel-based dermo-epidermal construct featuring an epidermal part that will correctly stratify upon grafting, a basement membrane, the characteristic structures of the dermo-epidermal junction, and a close to normal dermis[34]. These grafts, cultured from human cells, were successfully grafted onto immuno-incompetent rats[35–37] and, thereafter, a large animal

model (pig) allowing grafting of large (7 x 7 cm) laboratory grown autologous skin analogue[32,33] has been developed. These larger skin substitutes feature not only a near-normal anatomical architecture, but also mechanical properties allowing standard surgical handling, and are an appropriate size for the envisioned application in human patients.

With these preclinical studies successfully carried out, a first series of clinical applications was the next scientifically logical and ethically justifiable step, which started in June 2014 with the inclusion of the first patient in the phase I clinical trial denovoSkin(EHSG-KF)/denovoDerm (KEK-ZH-Nr. 2012-0573, Swissmedic 2013TpP1004). The clinical phase of the study concluded in June 2016 and the 5 year follow-up was completed in March 2021. As noted above, the grafts were applied successfully as autologous skin substitutes in children and young adults. Of note, at the time of grafting, two patients were 18 and 19 years old, demonstrating the successful use of EHSG-KF in young adult patients as well.

The further investigation of this innovative product EHSG-KF may offer great advantages. We feel that the promising results of the phase I clinical trial provide justification to proceed with the phase II clinical trial for EHSG-KF with the primary endpoint reflecting both safety and efficacy. The proposed phase IIb clinical trial will target adult and adolescent patients with severe burns, recognizing the potential benefit of a tissue-engineered autologous skin substitute that is not subject to donor site limitations, for this patient population.

The study will be led from the Zurich area in Switzerland, where there has been a 15 year-long collaboration between laboratory scientists and clinicians, and where the Good Manufacturing Practice (GMP) facility for the manufacturing of EHSG-KF is located, ensuring the best circumstances for the envisioned study.

## 1.2 Investigational Medicinal Product

EHSG-KF is an autologous cellular collagen hydrogel-based graft. The product is classified as a cell-based medicinal product (CBMP) in the US and an Advanced Therapy Medicinal Product (ATMP) in the EU and Switzerland.

It is a viable, tissue engineered autologous dermo-epidermal skin substitute. It is based on a plastically-compressed bovine collagen type I hydrogel seeded with human fibroblasts and overlaid by human keratinocytes (Figure 1). The dosage form of EHSG-KF is a square 45 ± 4 $cm^2$ graft with a thickness of 0.5 – 2 mm. A more detailed description of EHSG-KF can be found in the IB.

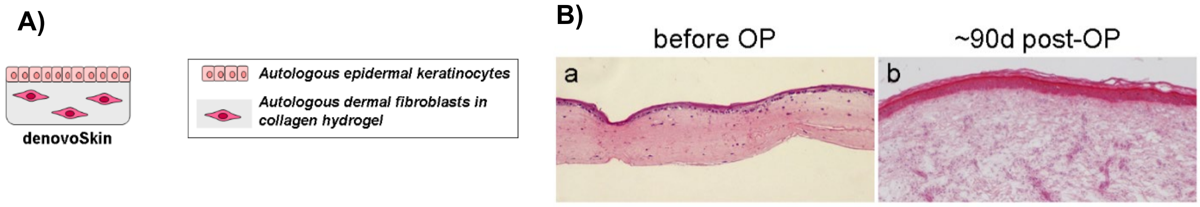


**Figure 1: EHSG-KF** is an autologous dermo-epidermal skin substitute based on a plastically compressed collagen type I hydrogel, seeded with autologous dermal fibroblasts and covered by autologous epidermal keratinocytes. A) Schematic view of EHSG-KF; B) Haematoxylin & Eosin staining of EHSG-KF a) before grafting (4x magnification) and b) approximately 90 days after grafting (10x magnification). a) fibroblasts (dark spots) are detectable in the dermal part of EHSG-KF. The epidermal part consists of 1-2 layers of keratinocytes. b) After grafting, fibroblasts are detectable in the dermal part of the biopsy, overlaid by a stratified epidermis, which was established by the thin keratinocyte layer present on top of EHSG-KF.

## 1.3 Benefit / Risk Aspects

The experimental product potentially offers a better therapeutic option than STSG alone. EHSG-KF has been successfully tested in the phase I clinical trial and several preclinical studies without significant adverse reactions and, as the skin grafts are autologous, no unusually high incidence of complications/adverse effects is anticipated. If the working hypothesis proves true, then graft take and wound healing dynamics will be similar to those of STSG, while the efficacy, in terms of scar quality and final functional and cosmetic results, will be better than obtained from STSG alone. Based on the available study results, we do not believe the risk associated with this product is greater than with the usual treatment.
Currently the product is under investigation and known as well as newly emerging risks are periodically assessed. New and updated risk mitigation strategies are included in the study protocol when appropriate.

### 1.3.1 Benefits of EHSG-KF

- Minimal donor site area, relative to STSG. A small biopsy site of only 4 $cm^2$ (as precise as possible) is required to generate a 45 ± 4 $cm^2$ piece of EHSG-KF, while the ratio of graft size to donor site for a conventional STSG is approximately 1:1.
  Although the latter ratio can be increased through meshing, the amount of expansion achieved is consistently less than expected based on the meshing ratio[38,39], and STSG quality is sacrificed[40].
- By reducing the donor site area, the wound healing burden placed on the patient is reduced, along with the donor site morbidity, such as pain, pruritus, textural and pigmentation changes, infection, bleeding, delayed wound healing, and hypertrophic scar formation.
- Due to backup procedures during the manufacturing of the skin analogue, secondary and even further manufacturing phases can be achieved with only one biopsy, without recruiting new donor sites, and with minimal additional time. From a 4 $cm^2$ biopsy, two sheets (90 $cm^2$) can be generated in an average of 32 days (SD 4, range 26-38 days). In approximately 35-44 days, an estimated 1000 $cm^2$ (20 sheets) of EHSG-KF could be manufactured from the 4 $cm^2$ biopsy, and, in 38-48 days, an estimated 2000 $cm^2$ (40 sheets) could be generated, as calculated using doubling time data acquired in phase 1, thereby offering a potentially life-saving intervention in the setting of severe burns with limited donor sites (burns greater than 40% TBSA). Without the production of skin substitutes in the setting of severe burns with limited donor sites, definitive coverage of burns is delayed until the previously harvested donor site has healed (approximately 7-14 days) and can thus be re-harvested for further grafting. Moreover, a higher graft quality can potentially be achieved with the use of EHSG-KF, when compared with the widely meshed, often thinner grafts available for coverage in large burns[40].
- Long-term scar quality of EHSG-KF is expected to be higher than a meshed STSG because EHSG-KF contains a dermal layer. This has been supported by the long-term results of the phase I trial, in which mean POSAS scores for thickness (2.88, range 1-5, SD 1.55), relief (3.00, range 1-6, SD 1.85), and pliability (3.13, range 1-8, SD 2.36), all of which reflect the risk of hypertrophic scarring, were found to be lower (and therefore better) than those seen in a comparable population with STSG (thickness 3.4, relief 3.4, and pliability 3.9) at one year post-operatively[41].
- The expected improvement in scar quality and confluent dermal layer should reduce scar contraction, and translate clinically to improved functional and aesthetic outcomes, when compared with meshed STSG.

### 1.3.2 Risks of EHSG-KF and Risk Mitigation Strategies

Standard surgical risks associated with biopsy harvest:

- Infection, bleeding, delayed wound healing, abnormal scar formation (hypertrophic, keloid scars), injury to local structures.

Standard surgical risks associated with grafting of EHSG-KF:

- Anaesthetic-related complications, infection, bleeding, delayed wound healing, and injury to local structures.
- Poor graft take due to infection, hematoma, or seroma; technical errors during grafting; and/or shearing/tangential forces on the graft, as with insufficient dressings or immobilization.
- Unfavourable scarring of EHSG-KF due to either patient factors or product factors (i.e. the hypothesis that EHSG-KF causes less scarring than STSG might prove to be incorrect, although clinical data from phase I support favourable scarring of the product).

In order to reduce the above mentioned risks as much as possible, all study interventions, including biopsy and grafting procedures, will be carried out by a surgeon with expertise in burn surgery at a specialized burn centre. Most participating burn centres are verified by the European Burn Association or analogous authority (see http://euroburn.org/burn-centres-2/verification-burn-centre/ for further information regarding Burn Centre Verification). The Sponsor provides Standard Operating Procedures (SOP) and Working Instructions (WI) to all study sites to aid in standardizing and optimizing the perioperative and operative care.
For patients in The Netherlands, the study will not be performed on the neck and the head area in order to be sure to avoid study related severe scarring in these very exposed areas of the body.
Furthermore, wound bed preparation, grafting, and postoperative wound management will be carried out in accordance with burn care and plastic surgical principles and standards that are widely understood and implemented at the study sites to reduce the risks of infection, bleeding/hematoma, and insufficient debridement, all of which may contribute to poor graft take and poor scar quality, as well as injury to local structures. Additional measures to reduce bleeding, such as spraying a fibrin glue to the wound bed prior to graft application, may also be implemented at the discretion of the surgeon. Post-operative dressings and/or splints will be selected and applied by the operating surgeon, and inpatient care between dressing changes will be carried out by an experienced multidisciplinary burn care team, under the guidance and supervision of the surgeon, to reduce the risk of graft manipulation or contamination, and for the early recognition of complications.

Additional risks and risk mitigation strategies associated with the use of EHSG-KF:

- Tumorigenicity: The safety of EHSG-KF was demonstrated with local tolerance tumorigenicity studies carried out in immuno-competent house pigs and SCID-beige mice, respectively, for further details refer to the IB. Likewise, clinical data from phase I, including the 1 year follow-ups have not revealed any signs of tumorigenicity. Thus, based on the scientific knowledge available currently, the probability of tumorigenicity is very low, however, there are not long-term experiences with this application in humans, thus a risk of EHSG-KF in terms of tumorigenicity has not been definitively excluded.
- Allergic reaction to a component of EHSG-KF: Patients with known allergies to bovine collagen, amphotericin B, gentamycin, penicillin, and streptomycin are excluded. The fibroblasts and keratinocytes are autologous and not expected to elicit an immune reaction.

- Rejection of EHSG-KF: The biological components of EHSG-KF consist of autologous skin cells, thus exhibiting no hazardous immunoreactive and graft rejection potential. A pre-clinical study and the clinical phase I trial did not show any immunoreactions to EHSG-KF.
- The patient's wounds are not open anymore at the time EHSG-KF is available: This is an inclusion criterion that is re-checked at the time of the transplantation of EHSG-KF. In order to avoid keeping wounds open specifically for the clinical trial, the experienced surgeon needs to justify why the wounds could not have been covered with the standard of care.
- EHSG-KF is applied to the wrong patient: EHSG-KF is an autologous product and cannot be used for any other patient than it has been manufactured for. Investigators and study site personnel are trained on this fact and it is mentioned in the protocols, the IB and on each label of EHSG-KF. Correspondence of batch and patient number are checked by trained study site staff, recorded and monitored. Transplantation of EHSG-KF on a different patient (allograft use) could result in immune reaction and rejection.

## 1.4 Dose Rationale

One to two grafts (41 - 98 $cm^2$) per patient will be grafted. In phase I, patients could not receive more than a single sheet of EHSG-KF (45 ± 4 $cm^2$), as the safety of the product remained unclear. However, with the successful completion of the phase I study, and the reassurance that there are no major safety concerns with the use of EHSG-KF, it is now important to study the effect of EHSG-KF when grafted over larger areas when possible. As noted, EHSG-KF could potentially one day offer a life-saving treatment to patients with severe burns and limited donor sites. The phase IIb study should better reflect this potential future large scale application.

It is also important to elucidate the effect that adjacent sheets of EHSG-KF could have on healing. Areas of graft loss that are located more centrally within the EHSG-KF covered area are isolated from potentially normal epithelial appendages that provide the necessary elements for skin wound healing. It is therefore critical that we demonstrate the capacity of the surrounding healed areas of EHSG-KF to fill such defects in a timely manner. Another benefit to studying adjacent sheets of EHSG-KF is that we can elucidate the nature of healing along the seams between grafts. With STSGs, there is a higher risk of hypertrophic scar formation along these seams, due in part to thinner dermis at the leading and trailing edges of grafts reflecting limitations of the harvest techniques, and the potential for gaps to be left at these seams. We hypothesize that hypertrophic scar formation will be reduced along the EHSG-KF seams given the sharp geometric edges, allowing adjacent grafts to be very carefully and precisely aligned, and providing consistent dermal thickness throughout the grafts.

Limiting the acceptable range to one to two grafts will aid patient recruitment, given the relative rarity of severe burns, the need for equally-sized control and experimental areas, and the four and a half weeks required for skin manufacturing. Furthermore, this limited range allowed for statistically-sound sample size calculations to be carried out.

## 1.5 Justification of Choice of Study Population

The current study will be conducted in adults and adolescents with acute burns, while a separate parallel phase IIb study will be conducted in paediatric patients up to < 12 years. This will reduce heterogeneity in the study populations, strengthening the validity of the results. It is our goal to elucidate the safety and efficacy of EHSG-KF for a broad patient population, since patients of all ages may benefit from a bioengineered skin analogue in the setting of severe burns.

In particular, it is important to include elderly patients in the current phase IIb study, as they are at particularly high risk of sustaining accidental burns, have higher rates of hospitalization than younger adults[1], and have less robust wound healing and thus are at higher risk of delayed STSG and donor site healing[40]. They also face a disproportionally high mortality rate compared with the general population, which could potentially be reduced with a bioengineered skin analogue to reduce donor sites, and speed coverage when autograft donor sites are limited.

## 1.6 Conclusion

In summary, EHSG-KF has been proven to be safe for the paediatric patients in the phase I clinical trial. Our multiple and carefully conducted preclinical studies, all of which are published in peer-reviewed leading journals in the field, together with the results of the phase I clinical trial, indicate that EHSG-KF can be reliably and reproducibly assembled at a GMP-facility and then successfully and safely grafted onto children and young adult patients. Thus, the next step is a further clinical application of EHSG-KF, covering larger areas, in the proposed phase IIb clinical trial in adults and adolescents, with emphasis on assessment of the efficacy of the product in comparison to standard treatment.

# 2. Study Objectives

**Overall Objective**
To evaluate the efficacy and safety of EHSG-KF in comparison to meshed STSG in adults and adolescents with deep partial and full-thickness burns.

## 2.1 Primary Objective

To evaluate the efficacy of EHSG-KF in comparison to meshed STSG based on:

- Ratio of covered surface area to biopsy site/donor site surface area 4 weeks post grafting

## 2.2 First Secondary Objective

To evaluate the efficacy of EHSG-KF in comparison to meshed STST based on:

- percentage of epithelialization at 3 months post grafting

## 2.3 Secondary Objective

To evaluate the safety and efficacy of EHSG-KF in comparison to meshed STSG based on the assessment of:

- Infection
- Scar quality:
  - Cutometer® 3, 6, 12, 24 and 36 months post grafting
  - DSM ColorMeter 3, 6, 12, 24 and 36 months post grafting
  - POSAS-questionnaire 3, 6, 12, 24 and 36 months post grafting
- Graft take at 6-10 days post-grafting
- %Epithelialization (to estimate 'time to complete epithelialization') at 3 and 4 weeks, and 2 and 6 months post grafting
- Incidence of wound closure at 4, 8, and 12 weeks post grafting
- Growth (% change in surface area, $cm^2$, between 1 and 3 years post grafting)
- Assessment and reporting of all observed adverse events
- Quality of life assessments of study patients based on the assessment of:
  - Patients ≥18years:
    - EQ-5D (EuroQol 5 Dimensions questionnaire)
    - BSHS-B (Burn Specific Health Scale – Brief questionnaire)
  - Patients <18years:
    - EQ-5DY (EuroQol 5 Dimensions questionnaire youth version)
    - PedsQL
- Healthcare resource utilization (direct and indirect healthcare costs)

# 3. Investigational Medicinal Product Information

EHSG-KF is an investigational medicinal product (IMP) supplied by the Sponsor. EHSG-KF is a tissue-engineered autologous dermo-epidermal skin substitute for the treatment of partial deep dermal and full-thickness skin burns.

## 3.1 Production of IMP

After biopsy harvesting, the biopsy will be shipped to the manufacturing site for IMP production. The manufacturing process of EHSG-KF consists of the following steps:

1. Biopsy preparation
2. Cell isolation (fibroblast and keratinocyte isolation)
3. Cell expansion (fibroblast and keratinocyte culture, fibroblast and keratinocyte passaging)
4. Gel preparation
5. Dermis *in vitro* cultivation
6. Keratinocyte seeding
7. Skin *in vitro* cultivation
8. Preparation of final product

A small piece of the tissue engineered product will be submitted to external service partners for quality control. Additionally, geometry, thickness and cell viability are checked. The samples will be destroyed in compliance with GMP-guidelines (GMP guide Annex 19). All details of the manufacturing process are described in the current version of the Investigational Medicinal Product Dossier (IMPD).

## 3.2 Packaging, Labelling, Supply, Accountability, Destruction

EHSG-KF is packaged at the manufacturing site, including appropriate labelling, and shipped to the study site. Upon receipt of the graft, transport conditions and the graft are checked according to working instructions and transport form signed by the person accepting the shipment. Any damaged or unusable graft in a given shipment will be documented in the study files.

### 3.2.1 Packaging

Packaging of EHSG-KF is performed at the manufacturing site according to internal GMP protocols. The primary packaging consists of a leak-tight TPP flask. Details can be found in the WIs.

### 3.2.2 Transport

After preparing for transport (see chapter 3.2.1), the TPP flask is shipped inside a qualified transport box. The temperature is monitored during transport and storage. The final product has an estimated maximum shelf life of 72h (time range between packaging at the GMP facility and clinical application). Date and time of packaging and shelf-life will be documented on the label and transport form. Date and time of clinical application will be documented in the study files and the corresponding control form.

### 3.2.3 Labelling

The labelling of the product and the packaging is performed according to internal GMP protocols. Details can be found in the WIs.

### 3.2.4 Supply

Each graft is supplied ready for use and intended for autologous application on a single patient. EHSG-KF is produced and packed under sterile conditions and free of mycoplasma or endotoxins. The grafts are delivered to the study sites maximally two days before the planned date for the surgical intervention. Details can be found in the WIs.

### 3.2.5 Storage Conditions

Upon arrival of the graft at the study site, the graft is stored inside the transport box. The complete transport box with the graft is stored at room temperature (15-25°C). Details can be found in the WIs.

### 3.2.6 Study Product Accountability

The IMP accountability and tracking of EHSG-KF will be provided by the maintenance of patient-specific logs to monitor amounts of shipment, grafting and destruction of the grafts. Investigators must maintain accurate and adequate records including dates, batch number, quantities received, individual usage, etc. Details can be found in the WIs.

### 3.2.7 Return or Destruction of Study Product

Rest of product or unused product will be destroyed at the study site and logged in the IMP accountability log or sent back to the manufacturer. Unused, explanted (e.g. due to graft failure) or expired grafts are destroyed by an authorised person or can be used for further analysis for research purposes after written informed consent of the patient / legal representative for the further use of data and biological material. Any destroyed IMP will also be recorded in the IMP Destruction Form. On completion/termination of the study, there will be a final reconciliation of IMP shipped, IMP grafted and IMP destroyed. Any discrepancies noted will be investigated, resolved, and documented by the investigational site.

### 3.2.8 Out of Specification Product

In case the product is out of specification (OOS) and cannot be released by the manufacturer, there is the possibility to administer the product nevertheless. The treating physician
can be provided with the manufacturer's evaluation of the risks and the out of specification product can be supplied to the investigator at his/her request. This is in line with EudraLex Volume 4, chapter 11.5 Administration of out of specification products. Details can be found in the IB and WIs.

## 3.3 Experimental Intervention

The treatment to be applied is a living autologous tissue-engineered skin substitute based on a collagen type I hydrogel scaffold (EHSG-KF). The dermo-epidermal graft, EHSG-KF, is produced from the patient's own cells, which are obtained from a 4 $cm^2$ (as precise as possible) split-thickness (or full-thickness in special cases after written confirmation of the manufacturer) healthy skin biopsy (refer to chapter 7.3.2 for further details regarding the biopsy). For each patient, one to two grafts (graft size: 45 ± 4 $cm^2$; graft thickness: 0.5 – 2 mm) are manufactured over approximately 26 - 32 days at a GMP facility of the manufacturer.

Once manufacturing of the EHSG-KF is complete, and when the patient's clinical condition allows, EHSG-KF is grafted by a routine skin grafting procedure. The surgery takes place in the operating theatre of the respective study centre and is performed by the Principal Investigator (PI) and/or an Investigator under routine sterile conditions under general anaesthesia or analgosedation. Wound bed preparation, grafting, and postoperative wound management will be carried out in accordance with plastic surgical principles and standards

that are widely understood and implemented at the study sites (refer to chapter 7.3.5 for further details regarding the grafting procedure). During the same operation the control area is covered with a STSG meshed with a 3:1 expansion ratio (refer to chapter 3.4 for further details regarding the control treatment).
Allocation of the treatment to the two study areas, the experimental and control areas, is performed as described in chapter 3.5 after wound bed preparation intraoperatively, or before start of the surgery if it is ensured that the randomisation allocation is not communicated to the treating surgeon before wound bed preparation is completed.

## 3.4 Control Intervention (Current Gold Standard)

The control intervention will consist of the current standard of care for coverage of large skin defects, meshed STSG. A meshing ratio of 3:1 has been selected in order to ensure a consistent meshing ratio amongst all control sites in the study, thereby reducing heterogeneity, whilst allowing for statistically-sound sample size calculations. This specific ratio has been selected to balance the advantages of skin graft expansion (i.e. achieving greater wound coverage in cases with donor site limitations) with the aesthetic and functional disadvantages of a widely meshed construct. Boyce used a similar, but slightly greater, ratio of 4:1 for the control STSG[25], but this reflects the different meshing ratios that are possible with various commercially available meshers. The centres in the current study will all have access to a mesher that yields grafts meshed at a 3:1 ratio.

The operations will be staged in accordance with the standard of care for severe burns. Excision of all deep partial thickness and full-thickness burns will be carried out as part of the acute management of the patient, typically during the first weeks. Temporary coverage of the excised burns will then be achieved with allograft or another means of coverage. While awaiting definitive coverage, the allograft may need to be changed, particularly if there is poor adherence to the wound bed, or signs of critical levels of microbial contamination. The use of dermal templates or other skin substitutes which have a therapeutic effect on the wound ground, for temporary coverage of the control or experimental area is not allowed. Integra might be used after consultation of the Sponsor and Coordinating Investigator. Important for the quality of the study is that there is an equal wound ground preparation for comparison between the patients.
STSGs will be harvested using an air-powered or electric dermatome with a thickness of 0.2-0.4 mm, from areas of healthy, unburned skin. These donor sites must be chosen according to the lesion pattern. Meshed grafts are fabricated with a meshing device. Both dermatomes and meshing apparatus are part of the basic instrumentation for operations to cover skin defects. Wounds outside the study area (including the experimental and control areas) will be treated according to the surgeon’s discretion.

## 3.5 Randomisation

Allocation of wound treatments will be performed using randomisation. One treatment modality will consist of treatment with EHSG-KF (experimental area), the other of meshed STSG (control area).

On each patient, there will be two locations, described as location A and location B, whereby the surface area ($cm^2$) of A = the surface area ($cm^2$) of B. Efforts will also be made to select areas as similar as possible i.e. regarding depth (see chapter 5.4). Assignment of the two areas is performed according to Table 1.

**Table 1:** Assignment of the two study areas

| Study area assignment | Body location | | | |
|---|---|---|---|---|
| Location A | left | lateral | cranial | proximal |
| Location B | right | medial | caudal | distal |

A randomisation list will be prepared before the start of the study and will be implemented in the web-based randomisation system. Patient allocation to randomisation group will be managed through the electronic case report forms (eCRF). A paper-based backup procedure is in place.
As noted in chapter 3.3, to avoid bias in wound bed preparation procedures, randomisation will ideally take place during the operation procedure, after completion of wound bed preparation. If this is not possible, randomisation can be performed before start of the surgery if it is ensured that the randomisation allocation is not communicated to the treating surgeon before wound bed preparation is completed. After having verified that the patient meets all inclusion criteria and has none of the exclusion criteria, the Investigator or study team member contacts the web-based randomisation system. The web-based randomisation system will sequentially use the randomisation list to assign the option to be retained for the patient, either option 1 or option 2, as described in Table 2. If use of the web-based randomisation system is not possible, the paper-based backup system can be used exceptionally, and randomisation in the eCRF must be made up as soon as possible.

**Table 2:** Options for assignment of study areas

| Option | Description | Treatment allocation for Location A | Treatment allocation for Location B |
|---|---|---|---|
| Option 1 | Location A is the experimental area<br>Location B is the control area | EHSG-KF | 3:1 meshed split-thickness skin graft |
| Option 2 | Location A is the control area<br>Location B is the experimental area | 3:1 meshed split-thickness skin graft | EHSG-KF |

## 3.6 Compliance with Study Intervention

Issues with patient compliance with the study intervention are not expected given that the graft procedure for the study product will be completed by the Investigator/surgeon. Once the patient has been discharged from hospital post-operatively, compliance issues may arise.
In conclusion, compliance issues that could affect the primary endpoint (i.e. compliance with treatment and during the early follow-up period) are not anticipated. However, compliance issues could also arise later in the treatment course if the patient does not fulfil their outpatient follow-up requirements, which could potentially influence the secondary and exploratory endpoints.
It is possible that the issue arises that a minor patient declines further participation by showing signs of resistance. In such a case, it is the responsibility of the Investigator that the minor patient will be excluded (in The Netherlands in accordance with the NVK behavioural code).

## 3.7 Concomitant Medication

Documentation of concomitant medication use will be recorded at each visit.
All routine drugs administered following skin defect closure by split-thickness skin grafting, including drugs against pruritus, local ointments, local antibiotic ointments, routine dressing devices etc. are allowed.
All concomitant and/or rescue treatments with the exception of standard perioperative medication have to be documented in the eCRF. The medication listed in Table 3 will not be documented in the eCRF (only in the patient record).

**Table 3:** Standard perioperative medication not to be documented in eCRF

| Active component | Active component |
|---|---|
| Midazolam | NaCl 0,9% |
| Propofol | Ephedrine |
| Alfentanil | Ondansetron |
| Fentanyl | Paracetamol |
| Atracurium | Sevoflurane |
| Ringeracetat-Glc. 1% | Atropine |
| Ketamine | Droperidol |
| Remifentanil | Nalbuphin |
| TLA-Solution (1000ml NaCl 0,9% + 1ml Adrenalin 0,1% + 6ml NaBic 8,4% + 50ml Xylonest 1%) | |

### 3.7.1 Prohibited Concomitant Medication

There are no known prohibited medications.

### 3.7.2 Allowed Concomitant Medication

All medications will be accepted.

### 3.7.3 Concomitant Medication to be used with Caution

There are no known medications to be used with caution.

### 3.7.4 Allowed Dressing

Mepilex Ag is the recommended dressing, especially until the first dressing change. However, all dressings commonly used with STSG are allowed.

# 4. Selection of Study Population

## 4.1 Inclusion Criteria

- Age: ≥ 12 years of age
- Deep partial thickness and/or full-thickness burns requiring surgical wound coverage
- Expected that ≥ 90 $cm^2$ of wound (not counting head and neck area for study patients in The Netherlands) will remain open at 4 weeks post burn despite proceeding with treatment in accordance with the standard of care. > 20% TBSA burns can be taken as guideline, but TBSA is not an inclusion criterion.
- Signed Informed consent from the patient or the parents/legally authorized representative

## 4.2 Exclusion Criteria

- Patients tested positive for HBV, HCV, syphilis or HIV
- Patients with known underlying or concomitant medical conditions that may interfere with normal wound healing (e.g. systemic skin and connective tissue diseases, any kind of congenital defect of metabolism including insulin-dependent diabetes mellitus, Cushing syndrome or disease, scurvy, chronic hypothyroidism, congenital or acquired immunosuppressive condition, chronic renal failure, or chronic hepatic dysfunction (Child-Pugh class B or C), severe malnutrition, or other concomitant illness which, in the opinion of the Investigator, has the potential to significantly delay wound healing)
- Severe drug and alcohol abuse
- Pre-existing coagulation disorders as defined by INR outside its normal value, PTT >ULN and fibrinogen <LLN prior to the current hospital admission and / or at the Investigator's discretion
- Patients with known allergies to amphotericin B, gentamicin, penicillin, streptomycin, or bovine collagen
- Previous enrolment of the patient into the current phase II study
- Participation of the patient in another study with conflicting endpoints within 30 days preceding and during the present study
- Patients expected not to comply with the study protocol (including patients with severe cognitive dysfunction/impairment and severe psychiatric disorders)
- Suspicion of non-accidental injury
- Pregnant or breast feeding females
- Intention to become pregnant during the clinical course of the study (12 months)
- Wounds in the head and neck area as study target area (only applicable for study patients in The Netherlands)
- Enrolment of the Investigator, his/her family members, employees, and other dependent persons

### 4.2.1 Contraception Methods

As to ensure appropriate contraception measures and avoid pregnancy in females post menarche, the Investigators are required to instruct the patients on the consistent and correct use of the chosen contraception at each visit during the clinical phase of the study (until visit 10). In addition, the Investigator instructs the patient to call immediately if the selected birth control method is discontinued or if pregnancy is known or suspected.

Highly effective methods of contraception are those that, alone or in combination, result in a failure rate of less than 1% per year when used consistently and correctly and include:

- Established use of oral, inserted, injected or implanted hormonal methods of contraception are allowed provided the patient remains on the same treatment throughout the clinical phase of the study (until visit 10) and has been using that hormonal contraceptive for an adequate period of time to ensure effectiveness.
- Correctly placed copper containing intrauterine device.
- Male condom or female condom used WITH a spermicide (this can be foam, gel, film, cream, suppository).
- Male sterilisation with appropriately confirmed absence of sperm in the post-vasectomy ejaculate.
- Bilateral tubal ligation or bilateral salpingectomy.

## 4.3 Criteria for Withdrawal / Discontinuation of Patients

### 4.3.1 Early Withdrawal of Patients

The patient himself/herself and the patient's parents/legally authorized representative have the right to withdraw the patient from the study at any time and for any reason.

### 4.3.2 Criteria for Early Withdrawal of Patients by Investigator

In case of matching one of the following criteria during the study, the patient is withdrawn from the study by the Investigator:

- The initial evaluation at the time of the biopsy is not confirmed in the further course of treatment and the new evaluation dictates that no skin grafting is necessary.
- Presence of infection at the study site(s), as per the clinical evaluation of an Investigator with expertise in burn surgery, which has not resolved by the time of the grafting procedure.
- Clear evidence of poor compliance of patient (e.g. not attending scheduled visits more than once without notification, not following medical advice concerning mobilization etc.). However, these patients will be followed up for safety purposes as per the schedule.
- Newly diagnosed or reported underlying medical conditions (up to the point of grafting) that meet the exclusion criteria (e.g. newly diagnosed diabetes mellitus during the study).
- Severe graft damage during transport from the manufacturing site to the operating room. Defect coverage is then performed in accordance with the standard of care. (The patient does not need to be withdrawn from the study if the Investigator and study patient agree to continue the study and it is expected that ≥90 cm$^2$ of wound (not counting head and neck area for study patients in The Netherlands) will remain open when newly produced IMP is ready, despite proceeding with treatment in accordance with the standard of care. IMP will then be produced from previously harvested cells or from a new biopsy.)
- Serious deterioration of the patient's general health status leading to switch to palliative care (up to the point of grafting).
- Patient's ineligibility for the study after biopsy due to poor cell numbers or vitality of the cells during the manufacturing process of the graft, and the patient and/or parents/legally authorized representative refuses to consent to have a second biopsy carried out for a second manufacturing process.
- Ineligibility of the graft for grafting (no release of the final product) as determined at the manufacturer's discretion and/or Investigator's discretion, and impossibility to manufacture a new graft either by absence of cryopreserved cellular stock or absence

of consent from the patient and/ legally authorized representative for a new biopsy. Graft manufacturing failure as described in this chapter will be documented in the eCRF.
- Patients with 100% loss of the grafted EHSG-KF will be withdrawn from the study following the Safety Follow-up at 1 year ± 30 days (visit 10).
- Minor patients show resistance to study participation, (in The Netherlands in accordance with the NVK behavioural code).

Furthermore, should a study patient experience an AE or SAE that meets the following criteria, the patient may be immediately discontinued from the study.
- Any AE or SAE in the judgment of the Investigator or the patient, justifies withdrawal due to its severity, its nature, or requirement for treatment, regardless of the causal relationship to the IMP
- Clinically relevant test procedure results which endanger the patient

### 4.3.3 Replacement of Patients

The following points describe the procedure of replacement/enrolment of patients:

- Drop-out prior to grafting: Patient is replaced
- Drop-out after grafting: Patient is not replaced

Patients will continue to be enrolled in the study until 12 patients were evaluated for primary endpoint (visit 6) (see chapter 10.2).

## 4.4 Data Collection and Follow-Up for Withdrawn Patients

In the case of premature withdrawal from the study, either due to patient preference or Investigator-led withdrawal, where possible, the reason(s) will have to be recorded on the ”Premature Study End” form. This form needs to be completed for each prematurely discontinued patient.

Patients who discontinue the study prematurely will take part in a safety examination including a physical examination, vital signs, and routine laboratory tests, if possible. The data should be recorded in the eCRF in the ”Premature Study End” form . Patients withdrawing after visit 10 due to 100% graft loss (before visit 10) do not need to perform the safety examination.
The PI needs to ensure appropriate, further medical care for the patient outside of the study. Ongoing AEs should be followed to resolution at the discretion of the Investigator. These follow-ups will not be recorded in the eCRF. Ongoing SAEs have to be followed up until recovery or stabilisation and need to be documented in the eCRF and reported according to chapter 9.

Data collected up to the time point of the withdrawal of the patient and data from the safety examination (if possible) will still be collected in the eCRF and analysed.

# 5. Study Design and Course of the Study

The proposed study is a Phase IIb, prospective, open-label, intra-patient randomised controlled, multicentre study.
The purpose is to evaluate the safety and efficacy of an autologous bio-engineered dermo-epidermal skin substitute for the treatment of partial deep dermal and full-thickness burns in adults and adolescents in comparison to autologous split-thickness skin grafts.

## 5.1 Study Centres

Investigational sites will be selected based on their expertise in this field and on their ability to conduct the study according to protocol requirements and ICH/local regulations.
Approximately ten investigational sites in Europe are expected to be part of the study. Approximately two additional sites, in the USA and in Canada, may be added during the course of the study.

## 5.2 Study Design

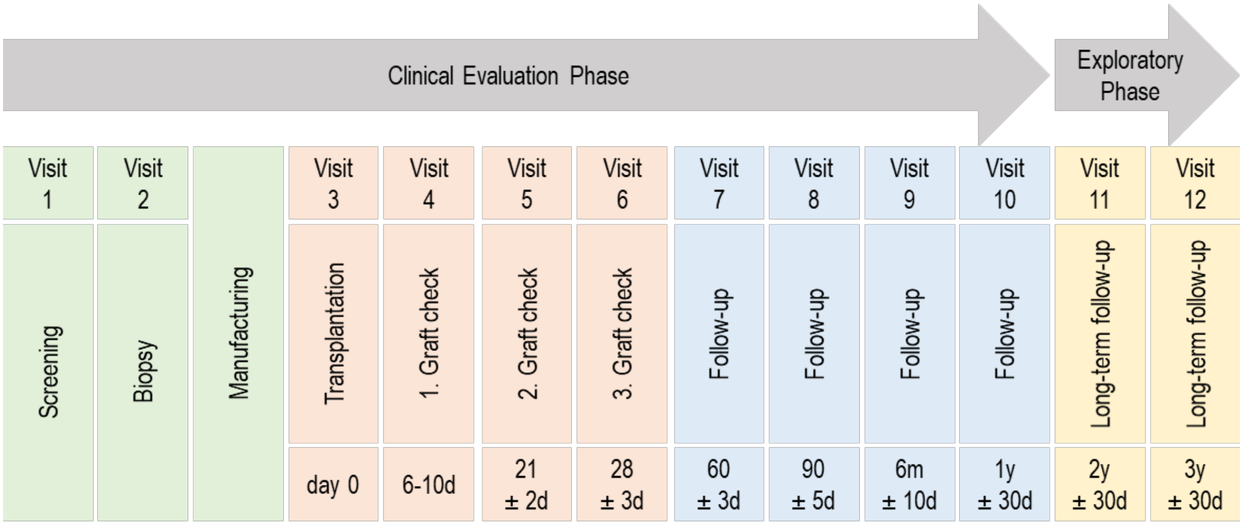


**Figure 2:** Scheme of Study Design. d: days; m: months; y: years

## 5.3 Study Procedures

<u>Invasive procedures:</u>

- Biopsy Nr. 1: mandatory biopsy, required for EHSG-KF production
- Biopsy Nr. 2: facultative histological examination of the wound ground at experimental area before wound bed preparation
- Biopsy Nr. 3: facultative histological examination of the wound ground at control area before wound bed preparation
- Blood samples for screening lab test
- Blood samples for routine lab test
- Grafting of EHSG-KF / STSG
- Biopsy Nr. 4: facultative histological examination of the wound ground at experimental area after wound bed preparation
- Biopsy Nr. 5: facultative histological examination of the wound ground at control area after wound bed preparation

<u>Non Invasive procedures:</u>

- Physical examinations
- Elasticity assessment of the skin
- Colour assessment of the skin
- Questionnaires (POSAS, EQ-5D and BSHS-B for patients ≥18 years; EQ-5DY and PedsQL for patients <18 years,)
- Photographic documentation of the study areas

<u>Optional invasive procedures:</u>

- Biopsies Nr. 6 and Nr. 7: optional biopsies at experimental area and control area at visit 8
- Biopsy Nr. 8 and Nr. 9: optional biopsies at experimental area and control area at visit 10

Details of all procedures are available in chapter 7.3. Prior to any study-specific procedure, written Informed Consent has to be obtained.

## 5.4 Selection of Study Areas

The study areas need to be as similar as possible depending on the individual situation of a patient. It is at the discretion of the Investigator to select appropriate study areas. The choice can be discussed with the Coordinating Investigator.

The following points should be considered for the selection:

- Body location: areas of high vs. low mobility (i.e. joints vs. chest), areas exposed to more or less sunlight (i.e. lower extremities vs. abdomen), areas exposed to more or less friction by clothes (i.e. lower abdomen vs. lower extremities)
- Type of wound; depth, wound ground (remaining dermis, fat, fascia, muscle, bone)
- Wound bed preparation; vascularization, bleeding

## 5.5 End of Study

The end of the clinical part of the study is reached when the last patient completes visit 10 (1 year follow-up visit). At this time point, data will be analysed and a Clinical Study Report (CSR) will be written. Visits 11 and 12 belong to long-term follow-up (over a period of 3 years post grafting). The end of the study coincides with the end of the long-term follow-up, which is reached when the last patient completes visit 12. At this point, data will once again be analysed and an Addendum to the CSR will be written.

# 6. Study Endpoints

## 6.1 Primary Endpoint

Efficacy evaluation, as a comparison between the EHSG-KF and control sites, based on:

- Ratio of covered surface area to biopsy site/donor site surface area at visit 6 (28 ± 3 days post grafting)

## 6.2 First Secondary Endpoint

Efficacy evaluation, as a comparison between the EHSG-KF and control site, based on:

- Epithelialization at visit 8 (90 ± 5 days post grafting)

## 6.3 Secondary Endpoints

Safety and efficacy evaluation, as a comparison between the EHSG-KF and control sites, based on:

- Main secondary safety endpoint:
    - Clinical and microbiologic signs of infection at visits 4 (6-10 days post grafting) and 5 (21 ± 2 days post grafting)
- Main secondary efficacy endpoints: Scar quality at the study areas
    - Assessment of elasticity of the study areas using the Cutometer® at visit 10 (1 year ± 30 days post grafting)
    - Assessment of general scar quality at the study areas using the POSAS, a reliable and validated scar assessment tool, at visit 10 (1 year ± 30 days post grafting)
- Other secondary safety endpoint:
    - Assessment and reporting of all observed adverse events will be carried out for the full duration of the study from visit 2 on
- Other secondary efficacy endpoint:
    - Epithelialization at visit 6 (28 ±3 days post grafting)

## 6.4 Exploratory Endpoints

- Graft take of the study areas assessed during the first dressing change at visit 4 (6-10 days post grafting) in a standardized manner as percentage of the whole grafted area.
- % Epithelialization (to estimate 'time to complete epithelialization') at:
    - visit 5 (21 ± 2 days post grafting)
    - visit 7 (60 ± 3 days post grafting)
    - visit 9 (6 months ± 10 days post grafting)
- Clinical and microbiologic signs of infection at:
    - visit 6 (28 ± 3 days post grafting)
- Incidence of wound closure at:
    - visit 6 (28 ± 3 days post grafting)
    - visit 7 (60 ± 3 days post grafting)
    - visit 8 (90 ± 5 days post grafting)
- Assessment of elasticity of the study areas using the Cutometer® at:
    - visit 8 (90 ± 5 days post grafting)
    - visit 9 (6 months ± 10 days post grafting)

- visit 11 (2 years ± 30 days post grafting)
- visit 12 (3 years ± 30 days post grafting)

- Assessment of scar colour (erythema and pigmentation) using the DSM ColorMeter® at:
  - visit 8 (90 ± 5 days post grafting)
  - visit 9 (6 months ± 10 days post grafting)
  - visit 10 (1 year ± 30 days post grafting)
  - visit 11 (2 years ± 30 days post grafting)
  - visit 12 (3 years ± 30 days post grafting)
- Growth (% change in surface area, $cm^2$, between 1 year (visit 10) and 3 years (visit 12) post grafting)
- Assessment of general scar quality at the study areas using the POSAS, a reliable and validated scar assessment tool, at:
  - visit 8 (90 ± 5 days post grafting)
  - visit 9 (6 months ± 10 days post grafting)
  - visit 11 (2 years ± 30 days post grafting)
  - visit 12 (3 years ± 30 days post grafting)
- QOL assessments (EQ-5D and BSHS-B for patients ≥ 18 years; EQ-5DY and PedsQL for patients <18 years) at:
  - visit 8 (90 ± 5 days post grafting)
  - visit 9 (6 months ± 10 days post grafting)
  - visit 10 (1 year ± 30 days post grafting)
  - visit 11 (2 years ± 30 days post grafting)
  - visit 12 (3 years ± 30 days post grafting)
- Healthcare resource utilization (direct and indirect healthcare costs) at:
  - visit 9 (6 months ± 10 days post grafting)
  - visit 10 (1 year ± 30 days post grafting)
  - visit 11 (2 years ± 30 days post grafting)

## 6.5 Duration of Patient Participation

Clinical study (12 months):

- First-Patient-In: Q1 2018
- Last-Patient-Out: Q4 2023

Long-term follow-up (2 years):

- First-Patient-In: Q1 2019
  Last-Patient-Out: Q4 2025

The study is subdivided into two parts as summarised in Table 4. The overall study duration (excluding long-term follow-up) for every patient is until study visit 10 (1 year ± 30 days post grafting) and includes a screening and skin tissue engineering phase before grafting. During the whole study, a total of 12 study visits are performed.
The end of the clinical part of the study is reached when the last patient completes visit 10 (1 year follow-up visit). Visits 11 and 12 belong to long-term follow-up (over a period of 3 years post grafting). The end of the study coincides with the end of the long-term follow-up, which is reached when the last patient completes visit 12.

**Table 4:** Overview on the two study parts of TBRU-dS-BA-PIIb

| Study part | Timeline | Visits and timelines |
|---|---|---|
| **1. Clinical part** | **~ 13 months** | Visit 1 (screening) |
| | | Visit 2 (biopsy) |
| | | Production of the tissue engineered autologous skin substitute EHSG-KF |
| | | Visit 3 (day 0, grafting) |
| | | Visit 4 (6-10 days post grafting) |
| | | Visit 5 (21 ± 2 days post grafting) |
| | | Visit 6 (28 ± 3 days post grafting) |
| | | Visit 7 (60 ± 3 days post grafting) |
| | | Visit 8 (90 ± 5 days post grafting) |
| | | Visit 9 (6 months ± 10 days post grafting) |
| | | Visit 10 (1 year ± 30 days post grafting) |
| **2. Long-term follow-up** | **2 years, starting after the clinical part (after visit 10)** | Visit 11 (2 years ± 30 days post grafting) |
| | | Visit 12 (3 years ± 30 days post grafting) |

CUTISS
personalized skin

# 7. Study Assessment

## 7.1 Study Flow Chart / Table of Study Procedures and Assessments

**Table 5:** Overview on the study schedule for TBRU-dS-BA-PIIb

**Study Schedule TBRU-dS-BA-PIIb**

| Study Phase | Pre-treatment phase | | | Treatment phase | | | | Post-treatment phase | | | | Long-term follow-up | |
|---|---|---|---|---|---|---|---|---|---|---|---|---|---|
| Visit | 1 | 2 | | 3 | 4 | 5 | 6 | 7 | 8 | 9 | 10 | 11 | 12 |
| Description | Screening | Biopsy | Manufacturing | Grafting | 1. Graft check | 2. Graft check | 3. Graft check | Follow-up | Follow-up | Follow-up | Follow-up | Long-term follow-up | Long-term follow-up |
| Day | | | | 0 | 6-10 | 21 ± 2 | 28 ± 3 | 60 ± 3 | 90 ± 5 | 6M ±10 | 1Y ±30 | 2Y ±30 | 3Y ±30 |
| Informed consent | X | | Manufacturing | | | | | | | | | | |
| Inclusion / Exclusion criteria | X | X | | X [9] | | | | | | | | | |
| Demographic Data | X | | | | | | | | | | | | |
| Medical History | X | | | | | | | | | | | | |
| Screening Laboratory [4] | X | | | | | | | | | | | | |
| Routine Laboratory [5] | X | | | X [9] | X | | | | | | | | |
| Pregnancy Test [6] | X | | | | | | | | | | | | |
| Concomitant Therapy | X | X | | X [9] | X | X | X | X | X | X | X | X | X |
| Adverse Events | | X [2] | | X [2] | X [2] | X [2] | X [2] | X [2] | X [2] | X [2] | X [2] | X [2] | X [2] |
| Photographic Documentation | | | | X | X | X | X | X | X | X | X | X | X |
| Physical Examination | X | | | X [9] | | | | | | | X | | |
| Vital Signs | X | X | | X | X | X | X | X | X | X | X | X | X |
| Skin Biopsy Nr. 1 | | X | | | | | | | | | | | |
| Facultative Biopsies Nr. 2-5 | | | | X | | | | | | | | | |
| Optional Biopsies Nr. 6-9 | | | | | | | | | X | | X | | |
| Grafting | | | | X | | | | | | | | | |
| Anaesthesia/ analgosedation | | X | | X | X [7] | X [7] | X [7] | | | | | | |
| Dressing Change | | | | | X | X | X | | | | | | |
| Signs of Infection | | | | X | X [2] | X [2] | X [3] | | | | | | |
| Wound Swabs | | | | X | X | X | X | | | | | | |
| Wound Closure | | | | | | | X [3] | X [3] | X [3] | | | | |
| Graft Take | | | | | X [3] | | | | | | | | |
| Epithelialization Percentage | | | | | | X [3] | X [2] | X [3] | X [2] | X [3] | | | |
| Ratio covered vs. donor area | | | | | | | X [1] | | | | | | |
| Surface Area Measurement | | | | | | | | | | | X [3] | | X [3] |
| Cutometer | | | | | | | | | X [3] | X [3] | X [2] | X [3] | X [3] |
| DSM ColorMeter | | | | | | | | | X [3] | X [3] | X [3] | X [3] | X [3] |
| Questionnaire POSAS | | | | | | | | | X [3] | X [3] | X [2] | X [3] | X [3] |
| Questionnaires QOL [8] | | | | | | | | | X [3] | X [3] | X [3] | X [3] | X [3] |
| Healthcare Resource Utilization | | | | | | | | | | X [3] | X [3] | X [3] | |

[1] Primary Endpoint
[2] Secondary Endpoint
[3] Exploratory Endpoint
[4] HIV, HBV, HCV, Syphilis
[5] Blood count, CrP
[6] In female patients post menarche or >14years
[7] Patient dependent
[8] EQ-5D and BSHS-B for adults, EQ-5DY and PedsQL for adolescents
[9] can be assessed max. 1 day before grafting

## 7.2 Procedures at each Visit

### 7.2.1 Overview of Study Visits

**Table 6:** Scheduled study visits of TBRU-dS-BA-PIIb

| **Study Visits TBRU-dS-BA-PIIb** | | | |
|---|---|---|---|
| Study Period | Visit | Purpose | Time range |
| Pre-treatment phase | 1 | Screening | N/A |
| | 2 | Biopsy Harvesting (Biopsy Nr.1) | N/A |
| | - | IMP Manufacturing | From date of biopsy to grafting |
| Treatment phase | 3 | Grafting | Day 0 (max. -1 day before grafting for some assessments) |
| | 4 | First graft check | 6-10 days post grafting |
| | 5 | Second graft check | 21 ± 2 days post grafting |
| | 6 | Third graft check | 28 ± 3 days post grafting |
| Post-treatment phase | 7 | Follow-up | 60 ± 3 days post grafting |
| | 8 | Follow-up | 90 ± 5 days post grafting |
| | 9 | Follow-up | 6 months ± 10 days post grafting |
| | 10 | Follow-up | 1 year ± 30 days post grafting |
| Long-term follow-up | 11 | Long-term follow-up | 2 years ± 30 days post grafting |
| | 12 | Long-term follow-up | 3 years ± 30 days post grafting |

### 7.2.2 Visit 1 - Pre-treatment phase (Screening)

Screening of patients is carried out based on the following:

- Informed Consent signed by patient/parents/legally authorized representative and Investigator
- Checking of inclusion and exclusion criteria
- Screening laboratory (HIV, HBV, HCV, and Syphilis) and pregnancy test (only in female patients post menarche or >14 years old and premenopausal patients (whereas premenopausal is defined as: <2 years after last menstruation and not surgically sterile))

Additional assessments:

- Demographic data (age, gender, ethnicity*)
- Medical history (include previous and current diseases, co-existing diseases, history of skin lesions, previous procedures/interventions regarding skin lesions, information regarding all current medications from visit 1 on)
- Physical examination
- Vital signs (heart rate, temperature, blood pressure)
- Routine laboratory (blood count and C-reactive Protein (CrP))
- Assessment/reporting of concomitant therapy

*Ethnicity will be assessed given the well-known relationship between various ethnicity-related skin types and risk of hypertrophic[42,43] and keloid[44] scar formation.

### 7.2.3 Visit 2 - Pre-treatment phase (Biopsy)

The following assessments have to be done prior to the biopsy harvest:

- Checking inclusion and exclusion criteria (still expected that ≥90 $cm^2$ of wound will remain open at 4 weeks post burn despite standard of care treatment; concomitant medical conditions; check results of screening laboratory tests and pregnancy test (if indicated))
- Vital signs (heart rate, temperature, blood pressure)
- Assessment/reporting of concomitant therapy
- Assessment/reporting of adverse events

In case the patient is eligible as required, a biopsy is performed.

- Skin biopsy (Biopsy Nr.1): Standard procedure of harvesting a split-thickness skin biopsy (or full-thickness skin biopsy in special cases after written confirmation of the manufacturer) of 4 $cm^2$ (as precise as possible)

In the case of a performed biopsy, but unsuccessful manufacturing process, a second biopsy can be harvested if the patient and/or parents/legally authorized representative consent to it.

In exceptional cases, at the discretion of the PI and after consultation with the Coordinating Investigator, the biopsy harvesting can be performed before the screening laboratory test results are available. This is based on the following rationale:

- HIV, HCV, HBV, and Syphilis screening laboratory is not performed for the patient's safety, but due to GMP regulation.
- HIV, HCV, HBV, and Syphilis test results must be available before the biopsy can be processed at the manufacturing site.
- In acute patients, time is very critical for the treatment process. Obtaining the screening results and shipping of the biopsy to the manufacturing site both usually take at least 24 hours each. Therefore, in severe burn patients, it can be in the interest of the patient to proceed with the biopsy harvesting without waiting for the screening laboratory, as long as the screening laboratory results will be available when the biopsy arrives at the manufacturing site.

Such procedure must be clearly documented in the Investigator Site File (ISF).

### 7.2.4 Visit 3 - Treatment phase (Grafting, day 0)

The treatment phase of the study is subsequent to the successful manufacturing process of EHSG-KF and comprises the assessment of baseline parameters and the grafting of EHSG-KF or STSG alone onto the wound bed during a surgical intervention.

Following assessments can be done maximally 1 day before grafting or on the day of grafting:

- Checking inclusion and exclusion criteria
- Assessment/reporting of concomitant therapy
- Physical examination
- Routine laboratory (blood count and CrP)

Following assessments have to be done on the day of grafting (day 0):

- Vital signs (heart rate, temperature, blood pressure)
- Wound swabs and clinical evaluation for signs of infection at the study areas
- Photographic documentation of study areas (before and after wound bed preparation, and after application of the control STSG and EHSG-KF)
- Assessment/reporting of adverse events
- Determination and randomisation of study areas (experimental and control areas)

- Facultative biopsies from each wound bed (experimental area and control area) before and after wound bed preparation (e.g. necrectomy, excision) prior to grafting for histological examination of the wound ground.
  - One representative punch biopsy in both wound areas (experimental and control areas) before preparing the wound bed (Biopsies Nr. 2 and 3)
  - Another punch biopsy (experimental and control areas) will be taken after preparing the wound bed (Biopsies Nr. 4 and 5)
- Grafting in the operation theatre
- Plan postoperative visit 4 (6-10 days post grafting)

### 7.2.5 Visit 4 - Treatment phase (First Graft Check, day 6-10)

- Assessment/reporting of concomitant therapy
- Assessment/reporting of adverse events
- Photographic documentation of study areas
- Vital signs (heart rate, temperature, blood pressure)
- Routine laboratory (blood count and CrP)
- Dressing change
- Assessment/reporting of local infection rate, including wound swabs and clinical evaluation for signs of infection
- Assessment of the graft take (percentage of the whole grafted area)
- Plan postoperative visit 5 (21 ± 2 days post grafting)

### 7.2.6 Visit 5 - Treatment phase (Second Graft Check, day 21 ± 2)

- Assessment/reporting of concomitant therapy
- Assessment/reporting of adverse events
- Photographic documentation of study areas
- Vital signs (heart rate, temperature, blood pressure)
- Dressing change
- Assessment/reporting of local infection rate, including wound swabs and clinical evaluation for signs of infection
- Assessment of epithelialisation of study areas
- Plan postoperative visit 6 (28 ± 3 days after grafting)

### 7.2.7 Visit 6 - Treatment phase (Third Graft Check, day 28 ± 3)

- Assessment/reporting of concomitant therapy
- Assessment/reporting of adverse events
- Photographic documentation of study areas
- Vital signs (heart rate, temperature, blood pressure)
- Dressing change
- Assessment/reporting of local infection rate, including wound swabs and clinical evaluation for signs of infection
- Planimetric analysis of the covered (healed) surface area of the experimental area and the control area. Assessment of the ratio of covered surface area to biopsy site or donor site surface area
- Assessment of epithelialisation of study areas and wound closure
- Plan postoperative visit 7 (60 ± 3 days after grafting)

#### 7.2.8 Visit 7 - Post-treatment phase (Follow-up, day 60 ± 3)

- Assessment/reporting of concomitant therapy
- Assessment/reporting of adverse events
- Photographic documentation of study areas
- Vital signs (heart rate, temperature, blood pressure)
- Assessment of epithelialisation of study areas and wound closure
- Plan postoperative visit 8 (90 ± 5 days after grafting)

#### 7.2.9 Visit 8 - Post-treatment phase (Follow-up, day 90 ± 5)

- Assessment/reporting of concomitant therapy
- Assessment/reporting of adverse events
- Photographic documentation of study areas
- Vital signs (heart rate, temperature, blood pressure)
- Assessment of scar quality of EHSG-KF in comparison to STSG alone using
    - Cutometer®
    - POSAS-questionnaire
    - DSM ColorMeter®-Measurement
- Optional biopsies: one from the experimental area and one from the control area (biopsies Nr. 6 and 7), for routine histology and immuno-histochemistry
- Assessment of epithelialisation of study area and wound closure
- QOL questionnaires
    - EQ-5D and BSHS-B for patients ≥18 years
    - EQ-5DY and PedsQL for patients <18 years
- Plan postoperative visit 9 (6 months ± 10 days after grafting)

#### 7.2.10 Visit 9 - Post-treatment phase (Safety-Follow-up, 6 months ± 10 days)

- Assessment/reporting of concomitant therapy
- Assessment/reporting of adverse events
- Photographic documentation of study areas
- Vital signs (heart rate, temperature, blood pressure)
- Assessment of epithelialisation of study area
- Assessment of scar quality using
    - Cutometer®
    - POSAS-questionnaire
    - DSM ColorMeter®-Measurement
- QOL questionnaires
    - EQ-5D and BSHS-B for patients ≥18 years
    - EQ-5DY and PedsQL for patients <18 years
- Health Costs after a Skin Transplantation
- Plan postoperative visit 10 (1 year ± 30 days after grafting)

#### 7.2.11 Visit 10 - Post-treatment phase (Safety-Follow-up, 1 year ± 30 days)

- Assessment/reporting of concomitant therapy
- Assessment/reporting of adverse events
- Vital signs (heart rate, temperature, blood pressure)

- Photographic documentation of study areas
- Assessment of scar quality using
    - Cutometer®
    - POSAS-questionnaire
    - DSM ColorMeter®-Measurement
- Surface area measurements ($cm^2$) for study areas via digital image analysis (to obtain a baseline value to calculate % change with growth)
- Physical examination
- QOL questionnaires
    - EQ-5D and BSHS-B for patients ≥18 years
    - EQ-5DY and PedsQL for patients <18 years
- Health Costs after a Skin Transplantation
- Optional biopsies: one from the experimental area and one from the control area (biopsies Nr. 8 and 9), for routine histology and immuno-histochemistry
- Plan postoperative visit 11 (2 years ± 30 days after grafting)

### 7.2.12 Visit 11 - Post-treatment phase (Safety-Follow-up, 2 years ± 30 days )

- Assessment/reporting of concomitant therapy
- Assessment/reporting of adverse events
- Photographic documentation of study areas
- Vital signs (heart rate, temperature, blood pressure)
- Assessment of scar quality using
    - Cutometer®
    - POSAS-questionnaire
    - DSM ColorMeter®-Measurement
- QOL questionnaires
    - EQ-5Dand BSHS-B for patients ≥18 years
    - EQ-5DY and PedsQL for patients <18 years
- Health Costs after a Skin Transplantation
- Plan postoperative visit 12 (3 years ± 30 days after grafting)

### 7.2.13 Visit 12 - Post-treatment phase (Safety-Follow-up, 3 years ± 30 days)

- Assessment/reporting of concomitant therapy
- Assessment/reporting of adverse events
- Photographic documentation of study areas
- Vital signs (heart rate, temperature, blood pressure)
- Assessment of scar quality using
    - Cutometer®
    - POSAS-questionnaire
    - DSM ColorMeter®-Measurement
- QOL questionnaires
    - EQ-5D and BSHS-B for patients ≥18 years
    - EQ-5DY and PedsQL for patients <18 years
- Surface area measurements (cm2) for study areas via digital image analysis (to obtain post-growth value to calculate % change with growth)

## 7.3 General Description of Study Procedures

### 7.3.1 Recruitment Procedure

Recruitment and screening of patients will be done by the Investigators during hospitalisation for an acute injury.
The patient or the parents/legally authorized representative will be invited to sign an informed consent and then will proceed with a screening visit to confirm the inclusions/exclusion criteria. To check the exclusion criteria of underlying medical conditions patients' histories are obtained and medical records are searched. No payments to study patients are given in compensation for time and effort of patients and/or parents/legally authorized representative.

### 7.3.2 Skin Biopsy for IMP Manufacturing

A split-thickness healthy skin biopsy measuring 4 $cm^2$ (as precise as possible) is harvested before the scheduled grafting date. This biopsy is used for the manufacturing of EHSG-KF. Prior to the biopsy procedure, patient eligibility for the study must be confirmed and, whenever possible, consent will be obtained from the patient and/or parents/legally authorized representative. In emergency situations, deferred consent may be acceptable (see chapter 12.8).
Ideally, the biopsy harvesting will be done during a regular scheduled dressing change, or routine excision and grafting procedure, as early as possible during the treatment course. The biopsy is taken under routine sterile conditions by the Investigator. The decision regarding the analgosedation or anaesthesia will be patient-dependent and will be performed according to routine standards at the study centre.

A split-thickness biopsy is the preferred option and should be used if any possible. In special cases, after previous written confirmation of the manufacturer, a full-thickness biopsy can be harvested.

For a split-thickness skin biopsy, the skin is tangentially excised with a thickness of 0.2 - 0.3 mm to ensure only a superficial dermal defect is created and can therefore heal spontaneously within approximately 7-10 days. The partial thickness excision may be carried out with an electronic or air dermatome, or manually using a Goulian blade, according to the preference of the surgeon. Alternatively, the partial thickness biopsy may be cut from a STSG harvested for coverage of another site, or from a CEA biopsy, as long as it is a minimum thickness of 0.2 mm. As noted above for the full-thickness biopsy, the biopsy surface area ($cm^2$) will be determined and recorded and this value will be used for the calculation of the primary endpoint. As noted above, an effort will be made to ensure that the surface area of the biopsy is as close to 4 $cm^2$ as possible. The biopsy site is then covered with an appropriate dressing, at the discretion of the surgeon.

For a full-thickness skin biopsy, the skin is sharply incised as an ellipse, for ease of closure, and is elevated in the subcutaneous plane. The site is selected according to available areas of healthy skin, and an effort will be made to conceal the resulting scar as much as possible (e.g. along the inguinal crease, or in the postauricular scalp), at the surgeon's discretion. The biopsy surface area ($cm^2$) will be determined and recorded and later used for the calculation of the primary endpoint (ratio of covered surface area to biopsy surface area, as per chapter 8.1). An effort will be made to ensure that the surface area of the biopsy is as close to 4 $cm^2$ as possible. Undermining of the skin edges may be carried out to reduce tension on the closure as needed. Closure is then carried out using either permanent or absorbable sutures, followed by a dressing as needed, at the discretion of the surgeon.

After the skin biopsy has been taken, the biopsy is immediately placed in biopsy medium, and transferred to the manufacturing facility according to the Sponsor-approved SOPs and WIs, which were developed in accordance with Quality-Management ICH Guidelines.

### 7.3.3 Laboratory Analyses

- Screening (visit 1): Screening laboratory tests and routine laboratory tests
- Grafting (visit 3): Routine laboratory tests
- First graft check (visit 4): Routine laboratory tests
- Safety examination after premature withdrawal (if applicable): Routine laboratory tests

Screening Laboratory Analyses:
To exclude an underlying infectious disease, the following analyses for infections will be assessed:

- HIV, HBsAg, HCV, Syphilis (4x7.5ml blood)
- Anti-HBc (10ml serum)
- In addition, a pregnancy test will be performed in female patients older than 14 years, or post menarche, or premenopausal (3ml blood).

Routine Laboratory Analyses:
To investigate the local infection rate in the blood, the following haematology tests are performed:

- Blood count (1.2ml blood)
- CrP (C-reactive Protein) (1.2ml blood)

### 7.3.4 Clinical Evaluation of Signs of Infection

For wound assessment clinical signs of infection are observed. This is done at visit 3 (prior to graft application), and post-operatively at visits 4, 5 and 6.
Clinical infection may be suspected when the following signs and symptoms are observed: swelling, heat, redness, pain, loss of function, purulent discharge or increased exudate, wound deterioration and/or fever.
Clinical assessment is evaluated using subjective (clinical evaluation by an experienced surgeon) and objective (microbiological wound swabs) measures. However, the clinical evaluation by an experienced surgeon is the most important element of this assessment. Microbial colonization is almost universal in the case of severe burns, and thus microbial swabs alone cannot be used to diagnose infection[45]. Microbial swabs are carried out to supplement the clinical findings and, when infection is present, to help guide antibiotic therapy. For classification, Investigators will distinguish between contamination, colonisation and infection, as defined in Table 7.

**Table 7:** Overview Clinical Evaluation of Signs of Infection

| Visit | Findings | Evaluation |
|---|---|---|
| 3 | There are no microbes | N/A |
| | There are microbes (consistent with normal skin flora) but no signs of inflammation | Colonisation |
| | There are microbes (not consistent with normal skin flora) but no signs of inflammation | Contamination |
| | There are microbes and signs of inflammation | Infection |
| 4, 5, 6 | There are no microbes | N/A |
| | There are microbes that were already detected on the wound swabs at the previous visit and there are no signs of inflammation | Colonisation |
| | There are microbes that were not detected on the wound swabs at the previous visit and there are no signs of inflammation | Contamination |
| | There are microbes and signs of inflammation | Infection |

### 7.3.5 Grafting

For each patient, one to two grafts (graft size: 45 ± 4 $cm^2$; graft thickness: 0.5 – 2 mm) are manufactured by tissue engineering. One produced graft has a size of 7 x 8 $cm^2$ of which a strip of 1 x 7 $cm^2$ is removed for quality analyses, which leads to a final graft size of 45 ± 4 $cm^2$. The grafts are manufactured in a GMP facility and are provided on the scheduled day of skin grafting or maximally two days before in a transport medium filled tissue culture flask. Identity of the product and patient is guaranteed by the GMP compliant labelling and traceability process according to EU Directive 1394/2007 (Article 11-12 and Appendix III) and the GMP Guide Annex 13 (Chapter “Labelling”, Article 26.-33).

After successful tissue-engineering of the autologous dermo-epidermal skin-graft, EHSG-KF is grafted by a routine skin grafting procedure. During the same operation the control area is covered with a STSG meshed with a 3:1 expansion ratio (see chapter 3.6). The surgery takes place in the operating theatre of the respective study centre or accordingly qualified operating theatre and is performed by the PI and/or the Investigator under routine sterile conditions. General anaesthesia or analgosedation is performed, depending on the patient, in accordance with the standards for skin grafting procedures at the study sites. Dermal templates are not allowed in the control nor the experimental area for methodological reasons.

Wound bed preparation, grafting, and postoperative wound management will be carried out in accordance with plastic surgical principles and standards that are widely understood and implemented at the study sites. More specifically, routine standardised preoperative procedures, including disinfection, are performed. The wound dressing is removed, along with the allograft that was providing temporary coverage (if applicable), and the skin defect is revealed. Meticulous wound bed preparation is then carried out, with surgical removal of all devitalized, grossly contaminated tissues, followed by haemostasis and further disinfection to create an optimal wound bed to support the graft.

Allocation of the treatment to the two study areas, the experimental and control areas, is performed as described in chapter 3.7. Before and after wound bed preparation, and prior to grafting of the EHSG-KF or meshed STSG, facultative biopsies can be taken from each study area for histological examination of the wound ground.

The surface area ($cm^2$) of donor skin harvested for coverage of the control area must be determined intra-operatively and recorded, as this value will be used for the calculation of the primary endpoint (ratio of covered surface area to donor site surface area, as per chapter 8.1). Application of the study product and the meshed STSG will then be carried out in accordance with the standard method for split-thickness skin graft application, taking care to ensure the grafts are correctly oriented with keratinocytes facing upwards. In an effort to reduce the risk of haematoma or seroma under the graft, the wound bed might be sealed with fibrin glue, at the discretion of the surgeon.
The grafts will be fixed to the wound bed using a method at the discretion of the surgeon, including sutures, staples, or tissue glue (e.g. Histoacryl®, TissueSeal, Ann Arbor, Michigan).

A dressing that provides protection against sheering forces, keeps the study areas clean, is free of cytotoxic agents, and is not required to be changed prior to visit 4 (6-10 days) will be selected at the discretion of the surgeon (e.g. Mepilex Ag). The Coordinating Investigator can be contacted to discuss suitable wound dressings.

Photographic documentation will be carried out intraoperatively for both the control and experimental areas before and after wound bed preparation, and following application/fixation of the STSG and EHSG-KF.
In all patients, the surgical procedure ends after applying standardised dressings and/or splinting.

Delay of Treatment:
Delay of grafting can occur due to certain circumstances (e.g.):

- In the case of an inoperable patient due to standard criteria (e.g. high fever, high dose vasopressor/inotropic support, poor general condition), a severe infectious disease, cold or flu prior to skin grafting, scheduled surgery must be postponed until the patient has recovered
- EHSG-KF does not meet product specifications
- Any reason beyond the Investigator's or patient's influence leading to a delay of scheduled surgery

CAUTION: If surgery has to be delayed for >72 hours (from the time the product is packed in the GMP facility), graft stability is lost and it is no longer eligible for grafting. Tissue-engineering will again take approximately up to 38 days, varying individually due to biological activity of autologous cells and whether new IMP will be produced starting from a new skin biopsy or from previously harvested and stored cells. In acute cases, if surgery has to be postponed for >72 hours and therefore graft stability is lost, and if time does not allow a new graft to be manufactured from the cryopreserved cellular stock or from a second biopsy, the wound has to be covered applying the standard procedure of care and the patient has to be withdrawn from the study.

With special regard to any patients whose biopsy in the course of cell cultivation yields insufficient cell numbers or proliferation rates, which can be determined at the latest 10 days after the biopsy, the Investigator has to be immediately informed by the manufacturer. The patient and/or parent/legally authorized representative have to be informed by the Investigator immediately and an alternative therapeutic approach has to be offered appropriate to the patient's condition. If the patient and/or legally authorized representative freely agree, a second biopsy might be harvested for a new production procedure which can also be offered to the patient as a therapeutic approach. Results of the cell harvest and/or cellular proliferation assays, have to be recorded in the patient's tracking log by the manufacturer and the study

site. All steps performed thereafter have to be documented in the patient's records by the study site.

Management of Poor Graft Take Rate and Slow Epithelialization:
If assessment of graft take at visit 4 (day 6-10 after grafting) shows unsatisfactory results (graft take < 50%), or if assessment of time to complete epithelialisation shows unsatisfactory results (time to complete epithelialisation >8 weeks), the choice of further management is at the Investigators' discretion. According to the management of poor graft take in routine skin grafting, this could mean either conservative management (wound care, dressing changes) until the wound is closed secondarily and healed, or surgical closure by applying the standard of care for this individual situation and patient.

### 7.3.6 Facultative Biopsies at Visit 3 (during Grafting)

Facultative biopsies can be taken from each wound bed (experimental area and control area) before and after wound bed preparation (e.g. necrectomy, excision) prior to grafting for histological examination of the wound ground. The surgeon evaluates whether these biopsies are necessary.

- If applicable, this punch biopsy (2 – 3 mm diameter) in both study areas (experimental and control area) will be taken before preparing the wound bed for verification of the level of burn/defect depth. (Biopsies Nr. 2 and 3)
- If so, after preparing the wound bed, before grafting, another punch biopsy (2 – 3 mm diameter) will be taken in both areas to test wound bed quality for routine histology, immuno-histochemistry. This is performed for this study specifically. (Biopsies Nr. 4 and 5)

### 7.3.7 Optional Biopsies at Visit 8 and 10 (~90 days and ~1 year after Grafting)

Three months post-operatively and one year post-operatively, one representative optional punch biopsy (2 - 3 mm diameter) of each study area will be taken for routine histology and immuno-histochemistry. The results can be used for scientific dissemination, teaching purposes, and could become a part of the registration dossier of EHSG-KF for market authorisation.

If the patient/parents/legally authorized representative refuse to take the biopsies at Visit 8 and/or visit 10, this does not lead to withdrawal of the patient from the study.

### 7.3.8 Photography

At visits 3 to 12 digital photos of the study areas are taken. The standardised photographs will be taken according to the WI and provided to the Sponsor.
These photos will allow the surface area ($cm^2$) of epithelialization for the experimental and control areas to be determined with digital image analysis in order to determine the primary endpoint ratios. Quantification of the wounds will be assessed using either a site-specific computer-based analysis system or assessed by the Investigator. Parts of the experimental area that have epithelialized, or are covered with the healed study product, will be manually marked, measured, and considered as "covered surface area". This value will then be used to determine the primary endpoint as noted in chapter 8.1.
Additionally, the photos are used to document the course of the study of each patient, can be used for scientific dissemination, teaching purposes, and could become a part of the registration dossier of EHSG-KF for market authorisation.
The photos will be saved on a secure server, according to the WI of the Sponsor.

### 7.3.9 Study Visits Outside Time Window

If the defined time window for a study visit cannot be met, the study visit should be performed as close to the allowed time window as possible. A visit after the given time window should be documented under the last planned visit and marked with a comment (e.g. deviation).
The same procedure applies to visits made before the scheduled time window; such a visit shall be documented under the next planned visit and marked with a comment (e.g. deviation). The comment should include a statement for the reason for not meeting the time window.

### 7.3.10 Unscheduled Visits

If additional visits are required, at the discretion of the Investigator, that are not scheduled according to the study protocol (e.g. due to an additional wound dressing change), this unscheduled visit shall be documented according to the hospital-internal guidelines (e.g. in the medical file of the patient) as well as in the eCRF as unplanned visit. If the situation permits, it is recommended that photographic documentation of study areas should be performed also at an unscheduled visit.

# 8. Assessments of Endpoints

## 8.1 Primary Endpoint

### 8.1.1 Skin Amplification

The primary endpoint, ratio of covered surface area to biopsy site/donor site surface area four weeks post grafting, was selected to test the hypothesis that EHSG-KF possesses greater skin amplification capabilities than split-thickness skin grafts. The covered surface area of the experimental and control areas refers to the total areas (in $cm^2$) that are healed, or epithelialized, and does not include areas characterized by open wounds.
The most important feature of EHSG-KF (being an engineered skin graft) is the fact that its use significantly reduces the harvest of large quantities of donor skin (standard of care) in burn patients for closure of burn injuries involving greater than 20% TBSA. The reduction in donor skin requirements implies reductions in donor site morbidity, numbers of skin-grafting operations, and intensive care days, but those data are not collected in this study since every patient received only 1 or 2 EHSG-KF. The chosen primary endpoint reflects this crucial feature by comparing the area that has “taken” on the wounds after 28 days in ratio to the size of the donor site harvested to graft.

Patients with large deep burns, particularly those involving greater than 40% total body surface area, have limited healthy skin graft donor sites. Clinicians face a dilemma in achieving definitive skin coverage in this patient population[11]. As noted in chapter 1.1, current options, including widely meshed STSGs and CEAs, fall short of providing an optimal solution. An alternative to STSGs that can be expanded from a small, stamp-sized skin biopsy would help address this dilemma and thus carries the potential to reduce morbidity and mortality in severe burn patients. This capacity for expansion represents the primary medical benefit of the study product.

Studies by Boyce et al. have similarly focused on this endpoint, demonstrating that the requirement for harvesting of donor skin for cultured skin substitutes (CSS) was less than for conventional skin autografts in patients with massive burns[25,27,28]. Boyce et al. showed that CSS can be expanded by approximately 67 times, 66 times, and 108 times the area of donor skin, in the 2002[27], 2006[28], and 2016[25] studies, respectively, compared with an estimated value of 4 for 4:1 meshed autograft. In the latter study, the CSS was also shown to substantially reduce mortality when compared to a similar population from the National Burn Repository[25].
The ratio of surface area covered at the experimental area to surface area biopsied, and the ratio of surface area covered at the control area to surface area harvested (i.e. control STSG donor site surface area) will be calculated at Visit 6 (28 ± 3 days post-grafting) and the two ratios will be compared. The covered surfaces areas ($cm^2$) for both the experimental and control sites will be determined using planimetric analysis at visit 6. Planimetric analysis will be used since the healed areas often have irregular shapes, with dimensions that are not conducive to measurement with a ruler.

In the absence of complications, such as hematomas and associated graft loss, four weeks should allow adequate time for healing of the STSG and EHSG-KF in order to reflect the long-term extent of coverage achieved by the two methods.

## 8.2 Secondary and Exploratory Endpoint

### 8.2.1 Epithelialization %, Time to Complete Epithelialization, and Incidence of Wound Closure

Assessment of epithelialisation of the study area is carried out in a standardised manner through the subjective assessment of the Investigator, in accordance with the standard of care[46], and expressed as a percentage of the whole grafted area. The '% epithelialisation' is assessed post grafting at visits 5, 6, 7, 8, and 9, and from these values the exploratory endpoint 'time to complete (100%) epithelialisation' will be extrapolated. More frequent study assessments to determine a more precise 'time to epithelialization' are not possible as this would place inappropriately excessive demands on both patients and Investigators.

The % epithelialization at visit 8 (90 ± 5 days post grafting) is the first secondary endpoint, the % epithelialization at visit 6 (28 ± 3 days post grafting) is another secondary endpoint, while the remainder of the % epithelialization values are exploratory endpoints. Visit 6 was selected for the secondary endpoint '% epithelialization' since this information will complement the primary endpoint ratio (see 8.1), which is also determined at visit 6, by providing information regarding healing of the grafts at the time of this assessment.
The exploratory endpoint 'incidence of wound closure', defined as the incidence of 100% epithelialization of the study areas, will be calculated at visits 6, 7, and 8.

The covered surfaces areas ($cm^2$) for both the experimental and control sites will be determined using planimetric analysis at visit 6. Planimetric analysis will be used since the healed areas often have irregular shapes, with dimensions that are not conducive to measurement with a ruler.

### 8.2.2 Graft Take

Graft take of the study areas are assessed during the first dressing change at visit 4 (4-11d after grafting) through the subjective assessment of the Investigator, in accordance with the standard of care[46], and expressed as a percentage of the whole grafted area.

### 8.2.3 Scar Quality

Assessment of the scar quality is carried out using three different parameters, POSAS (see chapter 8.2.4), Cutometer® (see chapter 8.2.5), and DSM ColorMeter® (see chapter 8.2.6).

### 8.2.4 POSAS

The POSAS questionnaire is a reliable and validated questionnaire. The POSAS consists of two numeric scales: The patient Scar Assessment Scale (patient scale), completed by the patient (variables: pain, itching, colour, stiffness, thickness and irregularity) and the observer Scar Assessment Scale (observer scale), completed by observer (variables: vascularisation, pigmentation, thickness, relief and pliability)[47–49].
The POSAS questionnaire is assessed at 12 months (visit 10) for the secondary endpoint, and 3, 6, 24, and 36 months post-grafting (visits 8, 9, 11 and 12) for the exploratory endpoints.

### 8.2.5 Cutometer®

Assessment of elasticity of the study areas will be carried out using the Cutometer® at 12 months (visit 10) for the secondary endpoint, and 3, 6, 24, and 36 months post-grafting (visits 8, 9, 11 and 12) for the exploratory endpoints.
Elasticity measurements for the study areas must be performed in accordance with the Information and Operating Instruction for the Cutometer® dual 580. Cutometer readings are taken from both study areas (i.e. experimental and control areas). In addition, readings are

taken from a matching normal (uninjured) skin area that is as anatomically comparable as possible to the study areas in the context of the severe burn. In cases where the anatomic site of the experimental and control areas is the same, a single matching normal skin area may be used. However, in cases where the anatomic sites of the experimental and control areas differ, two normal skin areas must be selected so that each of the study areas has its own matching normal skin area.
Skin/Scar elasticity parameters are used to calculate the ratio of each study area versus normal skin according to the following formula's (Table 8):

**Table 8:** Overview on Cutometer Values

| Description | Value | Formula |
|---|---|---|
| Pliability | Ua | R0-R1 and approximately the same R3-R4 |
| Retraction | Ur | R0*R7 |
| Elasticity | Ue | (R0*R7)/ R5 |
| Viscoelasticity | Uv | (R0*R7*R6)/ R5 |
| Maximum Extension | Uf | R0 |

Where R0 to R7 represent the output values from the Cutometer readings.

For each parameter the ratio of the study area versus normal skin is calculated according to:
Ua study area 1/ Ua normal skin.

### 8.2.6 Colour

Assessment of colour (erythema and pigmentation) of the study areas will be carried out using the DSM ColorMeter® at 3, 6, 12, 24, and 36 months post-grafting (visits 8, 9, 10, 11 and 12) for the exploratory endpoints. The DSM ColorMeter® measurements will be carried out in accordance with the Instructions for Use. Measurements will be performed on both study areas and comparable normal skin. As described for the Cutometer (see chapter 8.2.5), in cases where the anatomic site of the experimental and control areas is the same, a single matching normal skin area may be used. However, in cases where the anatomic sites of the experimental and control areas differs, two normal skin areas must be selected so that each of the study areas has its own matching normal skin area. The outcomes will be noted in the eCRF. Calculations will comprise the absolute mean difference between each respective study area and the normal skin, thus giving a measure for the deviation of the study area colour and pigmentation from normal skin colour and pigmentation.

After calibration of the instrument, measurements are performed on five random locations on each study area to prevent selection bias. The methodology is thus as follows:

- Measure experimental area (without applying extra pressure)
  - 5 points (provided that there is enough space)
- Measure control area (without applying extra pressure)
  - 5 points (provided that there is enough space)

Five measurement points are selected on healthy skin (as noted above), either on the contralateral side or, when this is not possible, on an adjacent location.

- Measure control spot (without applying extra pressure)
  - For limbs contralateral side
  - If not possible/measure other location adjacent

### 8.2.7 Clinical Signs of Infection

The local infection rate is evaluated using clinical evaluation by an experienced surgeon. Supplementary wound swabs will be carried out as noted in chapter 7.3.4. Since all severe local infections lead to poor graft take, there is a direct correlation between graft take and infection rate.
The local infection rate is assessed during the first and second dressing changes (visit 4, 6-10 days post grafting and visit 5, 21 ± 2 days post grafting) as secondary endpoints, and the third dressing change (visit 6, 28 ± 3 days post grafting, respectively) as an exploratory endpoint.

### 8.2.8 Number and Type of Adverse Events

Adverse events will be evaluated and counted as secondary endpoints.
Adverse events occurring from visit 2 (Biopsy) until visit 12 (3 years post-grafting) will be included.

### 8.2.9 Quality of Life Assessment

For patients ≥18years, both a general QOL measurement tool, the EQ-5D[50], and a burn-specific QOL measurement tool, the Brief Version of the Burn Specific Health Scale (BSHS-brief)[51], will be implemented in the current study in order to characterize QOL in our patient population and potentially allow for comparisons with the literature. Both of these QOL measurement tools have been validated in patients with burn injuries[50,51], as well as in the languages required for this multicentre study[52].
The EQ-5D is a standardized generic instrument for assessing health-related QOL. It consists of two components: a descriptive system and a visual analogue scale. The descriptive system encompasses five questions covering five dimensions: mobility, self-care, usual activities, pain and anxiety/depression, and for each the patient assigns a rating of 1 ('none'), 2 ('slight'), 3 ('moderate'). 4 ('severe'), or 5 ('extreme'). A unique health state is defined by combining the responses on each of the five dimensions, where 11111 is the best health state, and 55555 is the worst health state. The VAS is a vertical 20 cm line graded from 0 ('worst possible health state') to 100 ('best possible health state'), on which the respondent marks their own current state of health[50]. The questionnaire will be administered by a member of the research team, during the acute phase at 3, 6, 12, 24, and 36 months post grafting during the corresponding study visits, as the patient's condition allows.

The BSHS-B is a validated, shortened version of the original BSHS, an outcome scale designed specifically for burn patients to assess patient-reported quality of life measures. It consists of a 40-item questionnaire, divided into nine well-defined domains, which describe function with respect to Heat, Sensitivity, Affect, Hand Function, Treatment Regimens, Work, Sexuality, Interpersonal Relationships, Simple Abilities, and Body Image. Responses to each item are made by the patient on a 5-point scale (e.g. 0=extremely, 4=not/none at all)[51]. It will be administered by a member of the research team at the same time points as the EQ-5D (at visit 8-12) during the corresponding study visits, as the patient's condition allows.
Ideally, we would like to determine the QOL impact of the study product. However, the impact of the severe burn injury on a patient's QOL is tremendous[53], and, to the best of our knowledge, there is no QOL measurement tool that could elucidate the specific QOL impact of the relatively small area covered with EHSG-KF in the setting of a severe burn. Furthermore, we would like to determine the difference in the QOL impact of the study product in comparison to the STSG control. However, the intra-patient control design prohibits differentiation between the impact of the investigational product and the control intervention. Nonetheless, we believe that it is important to pilot these instruments as early as possible in order to gain experience with their execution and interpretation and to allow for refinement in their application in any future studies. We also believe that it is important to establish a baseline for these data in our clinical population to allow for validation or comparison in any future studies[54].

We acknowledge the importance of patient-reported outcome measures and the POSAS has also been selected for this reason (see 8.2.4 for further details).

For patients <18years, both a general QOL measurement tool, the EQ-5DY[51], and a paediatric quality of life assessment tool (PedsQL) will be implemented in the current study in order to characterize QOL in the adolescent patient population and potentially allow for comparisons with the literature. The EQ-5DY measurement tool has been validated in patients with burn injuries[50,51], as well as in the languages required for this multicentre study.
The EQ-5D-Y descriptive system comprises the following five dimensions: mobility, looking after myself, doing usual activities, having pain or discomfort and feeling worried, sad or unhappy. Each dimension has 3 levels: no problems, some problems and a lot of problems. The younger patient is asked to indicate his/her health state by ticking the box next to the most appropriate statement in each of the five dimensions. This decision results in a 1-digit number that expresses the level selected for that dimension. The digits for the five dimensions can be combined into a 5-digit number that describes the younger patient's health state. The EQ VAS records the younger patient's self-rated health on a vertical visual analogue scale where the endpoints are labelled "The best health you can imagine" and "The worst health you can imagine". The VAS can be used as a quantitative measure of health outcome that reflects the younger patient's own judgement.
The PedsQL Measurement Model is a modular approach to measuring health-related quality of life (HRQOL) in healthy children and adolescents and those with acute and chronic health conditions. The 23-item PedsQL Generic Core Scales were designed to measure the core dimensions of health as delineated by the World Health Organization, as well as role (school) functioning.

All questionnaires will be administered by a member of the research team at 3, 6, 12, 24, and 36 months post grafting) during the corresponding study visits, as the patient's condition allows.
Ideally, we would like to determine the QOL impact of the study product. However, the impact of the severe burn injury on a patient's QOL is tremendous[53], and, to the best of our knowledge, there is no QOL measurement tool that could elucidate the specific QOL impact of the relatively small area covered with EHSG-KF in the setting of a severe burn. Furthermore, we would like to determine the difference in the QOL impact of the study product in comparison to the STSG control. However, the intra-patient control design prohibits differentiation between the impact of the investigational product and the control intervention. Nonetheless, we believe that it is important to pilot these instruments as early as possible in order to gain experience with their execution and interpretation and to allow for refinement in their application in any future studies. We also believe that it is important to establish a baseline for these data in our clinical population to allow for validation or comparison in any future studies.

### 8.2.10 Healthcare Resource Utilization

Direct and indirect costs, which are likely to be significant drivers of total cost following a burn injury, will be collected. In-patient costs will be collected and recorded in monetary terms. Labour and social costs as well as out-patient costs will be collected and recorded as natural units, e.g. hours of physical rehabilitation, type of concomitant medication etc. Resources used in the management of any adverse events will be included. Direct and indirect costs will be combined for all patients so that they can be analysed in a descriptive way, and allow the future calculation of, for example, average direct (non-protocol-driven) costs per patient.

As the study design is based on an intra-patient control, the comparative analysis of costs associated with the two study areas (experimental and control) is not possible. In addition, the multinational nature of the study means that it may not be methodologically appropriate or feasible to compare costs between centres in different countries. Even with these caveats,

however, the data collected will assist in better characterizing the health economics of this patient population, and potentially allow for comparisons with the literature.
Patients will not receive any questionnaire related to the healthcare resource utilization analyses as the questions are not validated. However, the study team may ask them about it in a neutral way.

### 8.2.11 Additional Safety Measures

The following laboratory parameters will be assessed for safety purposes:

- Routine laboratory blood count
- C-reactive Protein

The following vital signs will be assessed for safety purposes:

- Heart rate
- Body temperature
- Blood pressure

### 8.2.12 Growth measurement

At visit 10, surface area measurements (in $cm^2$) for study areas are performed to obtain the baseline value for the growth measurement. At visit 12, surface area measurements (in $cm^2$) are performed to obtain post-growth values. With the baseline values and post-growth values, the percentage of change in size are calculated to obtain the % change of growth.

### 8.2.13 Assessments in Patients Who Prematurely Stop the Study

Patients who discontinue the study prematurely will take part in a safety examination including a physical examination, vital signs, and routine laboratory tests, if possible. Patients withdrawing after visit 10 due to 100% graft loss (before visit 10) do not need to perform the safety examination.

# 9. Safety

All adverse events (AEs) and all serious adverse events (SAEs) occurring from visit 2 (Biopsy) until visit 12 (3 years post-grafting) will be collected. They will be fully investigated and documented in source documents and eCRF.

## 9.1 Definitions of (Serious) Adverse Events and Other Safety Related Events

### 9.1.1 Adverse Events

AEs are defined as any untoward medical occurrence in a patient administered a pharmaceutical product and which does not necessarily have a causal relationship with the study procedures. An AE can therefore be any unfavourable and unintended sign (including an abnormal laboratory finding), symptom, or disease temporally associated with the investigational intervention, whether or not related to the IMP.
An AE may also consist of a new disease, an exacerbation of a pre-existing illness or condition, a recurrence of an intermittent illness or condition, a set of related signs or symptoms, or a single sign or symptom.

An abnormal test finding will be classified as an AE if one or more of the following criteria are met (according to the evaluation of the Investigator):

- The test finding is accompanied by clinical symptoms.
- The test finding necessitates additional diagnostic evaluation(s) or medical/surgical intervention, including significant additional concomitant drug treatment or other therapy. (Note: simply repeating a test finding, in the absence of any of the other listed criteria, does not constitute an AE.)
- The test finding leads to discontinuation of the patient's participation in the clinical study.

### 9.1.2 Serious Adverse Events

A SAE is any untoward medical occurrence that results in one or more of the following:

- results in death,
- is life-threatening,
  NOTE: The term "life-threatening" refers to an event in which the patient is at risk of death at the time of the event; it does not refer to an event which hypothetically might have caused death if it were more severe.
- requires in-patient hospitalization or prolongation of current hospitalization,
  NOTE: Complications that occur during hospitalization are AEs. If a complication prolongs hospitalization or fulfils any other serious criteria, the event is a SAE.
- results in persistent or significant disability/incapacity, or
  NOTE: The term disability is defined as a substantial disruption of a person's ability to conduct normal life functions. This definition is not intended to include experiences of relatively minor medical significance, such as uncomplicated headache, nausea, vomiting, diarrhoea, influenza, or accidental trauma (i.e., sprained ankle) that may interfere or prevent everyday life functions but do not constitute a substantial disruption.
- is a congenital anomaly/birth defect, or
- any other important medical events that may not be immediately life-threatening or result in death, or require hospitalization, but may jeopardize the patient or may require

intervention to prevent one of the other outcomes listed above should also usually (i.e. based on medical and scientific judgment) be considered serious. Examples of such events are intensive treatment for allergic bronchospasm, certain laboratory abnormalities (e.g. blood dyscrasias), convulsions that do not result in hospitalisation, or development of drug dependency or drug abuse.

### 9.1.3 Adverse Reaction and Suspected Adverse Reaction

All noxious and unintended responses to a medicinal product should be considered adverse reactions (AR). Suspected ARs are any AEs for which there is a reasonable possibility that the IMP caused the AE.
AEs associated with the use of the IMP outside what is described in the protocol, including misuse and abuse of the product, are considered as ARs.

### 9.1.4 Unexpected Adverse Drug Reaction

An “unexpected” AR is an AR, the nature or severity of which is not consistent with the applicable product information (IB EHSG-KF).

### 9.1.5 Suspected Unexpected Serious Adverse Reaction (SUSAR)

The Sponsor and Investigator evaluate any SAE that has been reported regarding seriousness, causality and expectedness. If the event is at least possibly related to the IMP and it is both serious and unexpected (the nature or severity of which is not consistent with the IB EHSG-KF), it is classified as a SUSAR.

### 9.1.6 Safety Signals

All suspected new risks and relevant new aspects of known ARs that require safety-related measures.

## 9.2 Recording of Adverse Events and Other Safety Related Events

Investigators and ultimately the PI have the primary responsibility for AE identification, documentation, and assessment i.e., with regard to seriousness, severity grading, and relatedness to the investigational intervention. This responsibility is applicable during the entire study period, regardless of the relation to the IMP.
Study patients will be instructed to report all AEs and will be routinely questioned about AEs at study visit 2 to visit 12 if possible. The well-being of the patients will be ascertained by neutral questioning (“How are you?”). All AEs will be followed until the event (or its sequelae) resolves or stabilizes at a level acceptable to the Investigators.

All observed or volunteered AEs will be fully documented in the patient file and subsequently recorded in the eCRF. For each AE, the information to be recorded in the source document and in the eCRF includes the nature of the AE, date and time of onset, seriousness, severity, causality (relationship to study product), duration, action taken and outcome of the event.
For all AEs, sufficient information will be pursued and/or obtained so as to permit an adequate determination of the outcome of the event (i.e., whether the event should be classified as a SAE) and an assessment of the causal relationship between the AE and the investigational study treatment. Even if the AE/SAE is assessed by the Investigator as not reasonably related to study product, its occurrence must be recorded in the source documents and in the eCRF. If an AE becomes a SAE, the corresponding eCRF entries are created.

Lack of efficacy per se will not be reported as an AE. The signs and symptoms or clinical sequelae resulting from lack of efficacy will be reported as an AE or SAE if they fulfil the AE or SAE definitions (including clarifications).

All AEs are reported in standardized terminology according to MedDRA adverse event coding dictionary version 18.1. Whenever available, the underlying disease or condition for which a therapeutic or diagnostic procedure is required should be reported as the AE term.

Surgeries or other invasive procedures that had already been planned prior to the enrolment into the study do not have to be documented as SAEs. These planned procedures will be recorded in the eCRF by the Investigator at the baseline visit or as concomitant therapy (whatever applies). It is not important if the condition was known before enrolment, only if the procedure was planned before.

### 9.2.1 Recording of Intermittent Adverse Events

AEs which occur intermittently during treatment phase and are well known accompanying symptoms also from standard treatment for (large scale) skin defects like:

- Pruritus,
- Pain,
- Granulation tissue,
- Increased temperature/ fever (acute cases),
- Increased heart rate/ tachycardia (acute cases), or
- Nausea (acute cases)

are documented once as “intermittent” with start point (first manifestation) and endpoint (last occurrence of the symptom) in the eCRF. Assessment of causality is done also for all intermittent AEs.

## 9.3 Assessment of Adverse Events and Other Safety Related Events

The Investigator promptly reviews AEs and abnormal test findings to determine if:

- the abnormal test finding should be classified as an AE (see chapter 9.1.1),
- the AE meets the criteria for a SAE (see chapter 9.1.2), and
- there is a reasonable possibility that the AE was caused by the IMP or study procedures (see chapter 9.3.1).

The severity of an AE will be assessed by the Investigator according to chapter 9.3.2.

### 9.3.1 Relationship Categorization

The Investigator is required to provide an assessment of relationship of all AEs and SAEs to the study procedure or the IMP. The Investigator should decide whether, in his or her medical judgment there is a reasonable possibility that the event may have been caused by the study procedures or the IMP itself, which includes the following events:

- AEs during the procedure for biopsy harvesting (including events linked to pharmaceutical products that are given to a patient with regard to an upcoming harvesting).
- Suspected microbial transmission from the IMP to the patient.

- AEs with a suspected link to a quality defect of the IMP or ingredient of the IMP (e.g. bovine collagen, media, antibiotics etc.).
- AEs with a suspected link to the application of the IMP (e.g. during grafting).

The assessment of causality to the IMP by the Investigator is done according to the following definitions (Table 9):

**Table 9:** Definition of relatedness

| Relatedness | Definition |
|---|---|
| Unrelated | • The event started in no temporal relationship to medicinal product applied and<br>• The event can be definitely explained by underlying diseases or other situations.<br>• An alternate aetiology has been established.<br>• The event does not follow the known pattern of response to study product.<br>• The event does not reappear or worsen with re-challenge. |
| Unlikely related / remote | • No temporal association to study product.<br>• Event could readily be produced by clinical state, environmental or other interventions.<br>• The event does not follow the known pattern of response to study product.<br>• The event does not reappear or worsen with re-challenge. |
| Possibly related | • Reasonable temporal relationship to study product.<br>• The event is not readily produced by clinical state, environmental, or other interventions.<br>• The event follows a known pattern of response to the study product or as yet unknown pattern of response. |
| Probably related | • There is a reasonable temporal association with the study product.<br>• The event is not readily produced by clinical state, environmental, or other interventions.<br>• The event follows a known pattern of response to the study product.<br>• The event decreases with de-challenge. |
| Definitely related | • There is a reasonable temporal association with the study product.<br>• The event is not readily produced by clinical state, environmental, or other interventions.<br>• The event follows a known pattern of response to the study product.<br>• The event decreases with de-challenge and increase with re-challenge |

If the causal relationship between an AE or SAE and EHSG-KF is determined to be at least possibly related (possibly, probably or definitely related), the event will be considered to be related to EHSG-KF for the purposes of expedited regulatory reporting.

### 9.3.2 Severity Grading

Severity grading of AEs and SAEs will be assessed using the Common Terminology Criteria for Adverse Events (NCI CTCAE, version 4.03). (Cf. http://evs.nci.nih.gov/ftp1/CTCAE/About.html).
In the event that an AE or SAE is not covered by the NCI-CTCAE grading system, the following definitions will be used (Table 10):

**Table 10:** Definition of Grades

| Grade | | Definition |
|---|---|---|
| Grade 1 | Mild | hardly noticeable, negligible impairment of well-being, awareness of sign, symptom, or event, but easily tolerated; does not interfere with usual daily activities or tasks |
| Grade 2 | Moderate | marked discomfort, but tolerable without immediate relief, discomfort enough to cause interference with usual activity and may warrant therapeutic intervention |
| Grade 3 | Severe | overwhelming discomfort, calling for immediate relief, incapacitating with inability to perform usual activities and daily tasks; or significantly affects clinical status, requires therapeutic intervention |
| Grade 4 | Life-threatening | immediate risk of death (Note: will require reporting as a SAE) |
| Grade 5 | Death | Note: will require reporting as a SAE |

An AE that is assessed as severe should not be confused with a SAE. Severity is a category used for rating the intensity of an event; both AEs and SAEs can be assessed as severe. An event is described as 'serious' when it meets one of the pre-defined seriousness criteria described in chapter 9.1.2.

### 9.3.3 Expectedness

Expectedness of SAEs will be determined by the Sponsor or its representative using the current version of the IB for the IMP.

## 9.4 Reporting of Serious Adverse Events and Other Safety Related Events

### 9.4.1 Reporting of SAEs

The Investigator is responsible for reporting any SAEs to the Sponsor immediately within 24 hours of the Investigator's first knowledge of the event, even if the event does not appear to be related to the IMP or the study procedures. Submission is done by entering and saving the SAE in the eCRF, which sends an automatic email to the Sponsor.
Initial SAE report: the initial SAE report must be as complete as possible, including details of the current illness and SAE, and an assessment of the causal relationship between the event and the IMP or study procedures.
The following information is a minimum set of information required for all initial SAE reports:

- Investigator name
- Patient identifiers
- AE term(s)
- Suspected IMP
- IMP relationship
- Reason why the event is serious

Follow-up SAE report: Information not available at the time of the initial report (e.g., an end date for the SAE, laboratory values received after the report, or hospital discharge summary) must be documented as a follow-up in the eCRF. Additional follow-up information, if required, or available, should be recorded in the eCRF and must be reported by the site every 14 days until resolution or stabilization of the SAE.

At any time after completion of the AE reporting period, if an Investigator becomes aware of a SAE that is suspected by the Investigator to be related to the IMP or study procedures, the event must be reported to the Sponsor or its representative.

In case reporting via eCRF is not working, a filled out SAE report form can be sent via:

- email to TBRU.safety@juliusclinical.com,
- fax to +31 30 65 69 996 (back up procedure for email submission), or
- post to Julius Clinical, Safety Group, Broederplein 41-43, 3703 CD Zeist, The Netherlands.

In case of SAE submission by fax or e-mail, the originals of the SAE report forms (both initial and follow-up report) will be collected by the monitor. The sites keep a copy for their own records.

### 9.4.2 Reporting of Safety Signals

All safety signals (definition see chapter 9.1.6) must be reported to the Sponsor within 24 hours of the Investigator's first knowledge. Submission is done as for SAE reports (see chapter 9.4.1).

### 9.4.3 IRB / IECs Safety Reporting by the PI

The local Institutional Review Board (IRB) and Independent Ethics Committee (IEC) must be informed by the PI or representative of the Sponsor about SAEs, SUSARs, safety signals, the Development Safety Update Report (DSUR) etc. as foreseen by local laws and applicable regulations.

The Sponsor or its representative will notify the Investigator of potential serious risks from clinical trials or any other sources, including the following:

- Suspected, unexpected SAEs.
- Suspected ARs that are both serious and unexpected (SUSAR).
- Any findings from other studies that suggest a significant risk in humans exposed to the IMP.
- Any finding from animal or in vitro testing that suggest a significant risk to humans exposed to the IMP, such as mutagenicity, teratogenicity, or carcinogenicity; or report of significant organ toxicity at or near the expected human exposure.

The DSUR contains information from all clinical trial sites. The Sponsor prepares it, and then submits it to the participating Investigators or Sponsor's representative which submit it to the IRB /IECs.
The Investigator must keep copies of all AE information, including correspondence with the Sponsor or Local Ethics Committees on file.

### 9.4.4 Regulatory Authorities Safety Reporting by the Sponsor

Reporting of SAEs, SUSARs, safety signals, the DSUR etc. to the Regulatory Authorities (RA) will be carried out by the Sponsor or its representative in accordance with the local laws and regulations.
The Sponsor's SOPs provide more detail on safety reporting to RAs.

## 9.5 Reporting and Handling of Pregnancies

Pregnancy per se does not classify as an AE. However, AEs related to a pregnancy have to be reported like any other AEs. Pregnancy should be confirmed by a reliable laboratory test.
In the event that a study patient becomes pregnant before visit 3 (grafting of the study product), she will be withdrawn from the study. Patients who become pregnant after visit 3, and thus

have already undergone grafting with the study product, will remain in the study for safety purposes.
All pregnancies occurring during the treatment phase of the study have to be reported to the Sponsor within the same timelines as for SAEs (see chapter 9.4.1)on the Initial Pregnancy Report Form.
The Investigator will contact the study patient at regular intervals by phone during pregnancy and after the estimated date of delivery to enquire about course and outcome of the pregnancy.

## 9.6 Follow-up of (Serious) Adverse Events

If a non-serious AE is ongoing at the date of the patient’s last visit, it will be followed up to resolution at the discretion of the Investigator.
If a SAE is ongoing at the date of the patient’s last visit, it has to be followed up until resolution or its stabilisation. The follow-up will be recorded in the eCRF.

Follow-up investigations may also be necessary according to the Investigator’s medical judgment even if the study patient has no ongoing AE at the end of the study.
All information has to be documented in the source documents.

In case of a study patient lost to follow-up, efforts should be made and documented to contact the patient to encourage him/her to continue study participation as scheduled. In case of mild (Grade 1) AEs (according to the Investigator’s discretion) a telephone call to the patient may be acceptable.

## 9.7 Anticipated Adverse Events

All complications that may be encountered during this study are AEs although they are, in all anticipated likelihood, not a direct consequence of the study product.
Rather, these complications or symptoms are well-known classical findings associated with any type of grafting of skin defects, namely infection, bleeding, unintended mechanical trauma (sheering forces acting upon fresh grafts) that may all lead to partial or complete graft loss. The management of these AEs encompasses additional dressing changes with wound cleansing/wound debridement (including vacuum-system-application), systemic and/or topical antibiotic therapy, and eventually re-grafting operations according to type and severity of the respective complication. In general, these complications are manageable in a straightforward way.

It is more difficult to envision AEs that are truly and solely arising from the use of EHSG-KF given the fact that all viable components are autologous and thus non-immunogenic, and all non-viable components (basically representing extracellular matrix components) have already been used in other clinical contexts without showing any significant AEs[33].

# 10. Statistical Analyses

Hypothesis:
The experimental treatment results in a better ratio of closed (healed) wound surface area to biopsy site surface area than the ratio of closed (healed) wound surface area to donor site surface area with the standard treatment. Apart from evaluating efficacy and safety of the intervention, this study is also intended to generate hypotheses for upcoming phase III studies.

## 10.1 Analysis Plan

### 10.1.1 Final analysis

The final analysis will be conducted once all patients have completed the month 12 end of study visit (V10).

### 10.1.2 Long-term follow-up analysis

The long-term follow-up analysis will be conducted once all patients have completed the 2 follow-up visits, 2 and 3 years after denovoSkin™ application, V11 and V12 respectively.

### 10.1.3 Interim analysis

In the interest of the development of the product, the Sponsor might extract data by means of an interim analysis from the first 12 patients who received denovoSkin™ to perform a submission to a regulatory agency for a request of designation such as for example the priority Medicines scheme (PRIME) in Europe and the Regenerative Medicine Advanced Therapy (RMAT) in the US. This will not interfere with the conduct of the study for the following up to 3 patients. The interim analysis will be done on a database that has been cleaned up to that the month 3 visit (V6) and will be performed on part of the efficacy and safety endpoints. The interim analysis will include the statistical analysis on the primary efficacy endpoint (final results will not change) and only descriptive statistics on the other endpoints, described in an interim-specific SAP (statistical analysis plan). A correction for multiple comparisons is considered not needed.

## 10.2 Determination of Sample Size

The primary endpoint (i.e. the ratio of covered surface area to biopsy site / donor site surface area at 4 weeks post grafting) will be analysed after a logarithmic transformation and the treatment effect estimate will be expressed as a percentage of additional coverage obtained with EHSG-KF comparatively to STSG.

Published data of cultured skin substitutes in a series of 10 patients (Boyce 1995, Table 11) show on the logarithm of the ratio of covered surface area to biopsy site surface area (also named expansion ratio) at 1 month post grafting a standard deviation of 0.97 with a mean of 1.98 (corresponding to a geometric mean of 7.24 on the original scale). Data from the EHSG-KF Phase I clinical trial (excluding one patient with 100% graft loss attributable to patient noncompliance and manipulation of the dressing post-operatively) show on the logarithm of the ratio of covered surface area to biopsy site surface area at 3 weeks post grafting a standard deviation of 0.99 with a mean of 1.80 (corresponding to a geometric mean of 6.05 on the original scale). A standard deviation of 1.00 will be assumed for the difference (on the logarithmic scale) between the two treatments, corresponding, for instance, to a common standard deviation of 1.00 on each treatment site with a correlation between sites of 0.50.

The ratio of covered surface area to biopsy site / donor site surface area at 4 weeks post grafting is expected to be at least 145% higher on EHSG-KF than on STSG (corresponding, for instance, to ratios of 7.35 and 3.00, respectively).

- Assuming a standard deviation of 1.00 for the paired difference between the 2 log(ratios), a sample size of 12 evaluable patients would be required in order to achieve a power of 80% to detect a treatment effect corresponding to an increase of 145% with EHG-KF (i.e. to a difference of 0.90 on a logarithmic scale) when using a one-sample t-test at two-sided significance level of 5%.
  A claim of study success will be made when the two-sided test passes the significance level of 0.05.
- All secondary endpoints will be tested in a strictly exploratory fashion only.

**Table 11:** Criteria for sample size determination

| Criteria | Value |
|---|---|
| Standard deviation of the paired difference between log(ratio) EHSG-KF and log(ratio) STSG | 1.00 |
| Difference in log-ratio to detect (effect size) | 0.90 |
| Statistical power | 0.80 |
| *P*-value (two-sided) | 0.05 |

## 10.3 Planned Analyses

### 10.3.1 Analysis Populations

Since randomization takes place during the operation procedure (or shortly before, as described in chapter 3.5), it is assumed that there is no difference between "randomized" and "treated" patients. Therefore, the following analysis populations are defined.

mITT (modified ITT) population: consists of all randomized/treated patients having at least 1 post-surgical measurement. The mITT population will be used for the analysis of efficacy variables, and statistical analysis will be done “as randomized”.

PP (per protocol) population: consists of all patients adhering to the protocol. The PP population is subset of the mITT population. Subjects with major protocol deviations that might impact the primary endpoint will be excluded from this population. These deviations will be

determined before database lock. The PP population will be used for the analysis of efficacy variables, and statistical analysis will be done “as randomized”.

SAF (safety) population: includes all patients who have received a surgical treatment, irrespective of satisfying other criteria. This population will be used for the analysis of safety and tolerability, and statistical analysis will be done “as treated”.

The primary analysis will be performed using the mITT as primary analysis population, and PP as supportive. Secondary and exploratory efficacy analyses will only use the mITT. Safety analyses will be presented using the SAF.

Implications of administration of OOS products (see chapter 3.2.8) on analyses are expected to be minimal considering that only OOS products with a positive risk/benefit ratio on patient safety will be administered. Data of patients with OOS products administered are included in the analyses.

.

### 10.3.2 Primary Analyses

The primary endpoint (i.e., the ratio of covered area to biopsy site / donor site surface area at 4 weeks post grafting) will be tested with a one-sample t-test applied on the difference between log(ratio) EHSG-KF and log(ratio) STSG. The treatment effect estimate and its 95% confidence limits obtained on the logarithmic scale will be expressed as percentage of increase of EHSG-KF relatively to STSG by applying the transformation [x-> (x-1)*100]. A claim of study success will be made when the two-sided test of the primary endpoint passes the significance level of 0.05.
As a robustness analysis, the difference between log(ratio) EHSG-KF and log(ratio) STSG will also be tested with one-sample Wilcoxon test (signed-rank test). The corresponding treatment effect estimate and its 95% confidence limits will be derived from the median of this difference by applying the transformation [x-> (x-1)*100].

### 10.3.3 Secondary Analyses

In addition to the primary endpoint, all secondary endpoints listed in chapter 6.3 will be evaluated and tested for differences between interventional and standard treatment. The obtained p-values will be interpreted in a strictly exploratory fashion only, and no correction for multiple comparisons will therefore be applied. Statistical analyses for (secondary and exploratory) endpoints measured at multiple timepoints will focus on repeated measure models, to address the dependencies between visits. This will be a Generalized Estimating Equation model in case of binary data, and a mixed-effects model in case of continuous data. If data are not fulfilling underlying model assumptions, a transformation may be necessary before applying the statistical model. Details will be described in the SAP.

### 10.3.4 First Secondary Analyses – Epithelialization at Visit 8

The percentage of epithelialization of a site is the percentage of the grafted area which is closed/healed with dry epithelium, as noted in chapter 8.2.

### 10.3.5 Secondary Analyses - Clinical and Microbiological Signs of Infection at Visits 4 and 5:

At each of the 2 visits, infections which can be linked to one of the two sites will determine for each patient whether an infection occurred on either site.
Infections which cannot be linked to a site will be listed.

### 10.3.6 Secondary Analyses – Scar Quality (Elasticity of the Study Areas at Visit 10 (Cutometer®))

The Cutometer® parameters (ratio of study site versus matching normal skin for both study areas) used to assess elasticity will be analysed comparing the difference between the 2 study areas. The estimate for the treatment difference will be provided with its 95% confidence interval.

### 10.3.7 Secondary Analyses – Scar Quality (General Quality at Visit 10 (POSAS))

The 6 patient variables, the patient overall opinion, the 6 observer parameters, their sum and the observer overall opinion will be analysed. 8.2.4.

### 10.3.8 Secondary Analyses – AE Reporting (during full study duration)

All adverse events will be listed by patient, phase (pre-treatment, treatment, post-treatment) and date of onset.
Adverse events with an onset date during the treatment phase will be summarized in a frequency table. Adverse events with an onset date during the pre-treatment phase or the post-treatment phase will be summarized the same way.
If appropriate, additional tables will be prepared for selected adverse events (e.g.: adverse event leading to treatment discontinuation, serious adverse events).

### 10.3.9 Secondary Analyses – Epithelialization at Visit 6

The percentage of epithelialization of a site is the percentage of the grafted area which is closed/healed with dry epithelium, as noted in chapter 8.2.

### 10.3.10 Exploratory Analyses

In addition to the primary endpoint and to the secondary endpoints, all exploratory endpoints listed in chapter 6.4 will be evaluated and tested for differences between interventional and standard treatment. The obtained p-values will be interpreted in a strictly exploratory fashion only, and no correction for multiple comparisons will therefore be applied. Statistical analyses for (secondary and exploratory) endpoints measured at multiple timepoints will focus on repeated measure models, to address the dependencies between visits. This will be a Generalized Estimating Equation model in case of binary data, and a mixed-effects model in case of continuous data. If data are not fulfilling underlying model assumptions, a transformation may be necessary before applying the statistical model. Details will be described in the SAP.

QOL assessments (EQ-5D and BSHS-B) and direct resource utilization cannot be used to compare interventional and standard treatment since these data are relevant at patient level and not at the site level. As a consequence, their analysis will be purely descriptive (without any p-value added).

### 10.3.11 Exploratory Analyses - Graft Take at Visit 4

The graft take of a site is the percentage of the grafted area which is closed/healed with dry epithelium.

### 10.3.12 Exploratory Analyses - Epithelialization at Visits 5, 7, 9

This variable will be analysed at each visit 10.3.4. In addition, a time-to-event analysis (to analyse the time to to the first observation of a complete epithelialization) will be done, by presenting a Kaplan-Meier plot and performing a paired/clustered log-rank test to assess a difference between the two sites.

#### 10.3.13 Exploratory Analyses – Clinical Signs of Infection at Visit 6

Signs of infection at visit 6 will be analysed as described in chapter 10.3.5.

#### 10.3.14 Exploratory Analyses – Incidence of Wound Closure at Visits 6, 7, and 8

At each of the three visits, the wound will be classified as closed or as not closed for each of the 2 sites.

#### 10.3.15 Exploratory Analyses – Scar Quality, Elasticity of the Study Areas at Visits 8, 9, 11 and 12 (Cutometer®)

The Cutometer® parameter used to assess elasticity will be analysed at each visit10.3.6.

#### 10.3.16 Exploratory Analyses – General Scar Quality at Visits 8, 9, 11 and 12 (POSAS)

The 6 patient variables, the patient overall opinion, the 6 observer parameters, their sum and the observer overall opinion will be analysed at each visit10.3.7.

#### 10.3.17 Exploratory Analyses – Scar Erythema and Pigmentation at Visits 8, 9, 10 11 and 12 (DSM Colormeter®)

Both study areas and comparable normal skin sites will be measured following the agreed WI. Differences between the values for each study site and matching normal skin site will be calculated.

#### 10.3.18 Exploratory Analyses – Quality of Life Assessment at Visits 8, 9, 10, 11, 12

For patients ≥18years, EQ-5D and BSHS-B data will be collated and analysed per patient but no comparative analysis will be undertaken.
The score of EQ-5D will be calculated according to the EQ-5D user guide (Paul Dolan Medical Care V35, Num 11, pp 1095-1108, 1197). The values at 3, 6, 12, 24, and 36 months and the changes from 3, 6, 12, 24 and 36 months of the EQ-5D score and of the EQ-5D VAS will be summarized by the usual descriptive statistics (mean, standard deviation, median, quartiles). The same approach may also be applied to each of the 5 dimensions (mobility, self-care, usual activities, pain-discomfort, and anxiety/depression).
The same approach will be applied to the BSHS-B.

For patients <18years, EQ-5DY and PedsQL data will be collated and analysed per patient but no comparative analysis will be undertaken.
The score of EQ-5DY will be calculated according to the EQ-5DY user guide (Paul Dolan Medical Care V35, Num 11, pp 1095-1108, 1197). The values at 3, 6, 12, 24, and 36 months and the changes from 3, 6, 12, 24 and 36 months of the EQ-5DY score and of the EQ-5DY VAS will be summarized by the usual descriptive statistics (mean, standard deviation, median, quartiles). The same approach may also be applied to each of the 5 dimensions (mobility, self-care, usual activities, pain-discomfort, and anxiety/depression).
The same approach will be applied to the PedsQL score according to the Scoring Guidelines of PedsQL.

#### 10.3.19 Exploratory Analyses – Healthcare Resource Utilization

Direct and indirect costs, which are likely to be significant drivers of total cost following a burn injury, will be collected. In-patient costs will be collected and recorded in monetary terms. Labour and social costs as well as out-patient costs will be collected and recorded as natural units, e.g. hours of physical rehabilitation, type of concomitant medication etc. Resources used in the management of any adverse events will be included. Direct and indirect costs will be

combined for all patients so that they can be analysed in a descriptive way, and allow the future calculation of, for example, average direct (non-protocol-driven) costs per patient.
As the study design is based on an intra-patient control, the comparative analysis of costs associated with the two study areas (experimental and control) is not possible. In addition, the multinational nature of the study means that it may not be methodologically appropriate or feasible to compare costs between centres in different countries. Even with these caveats, however, the data collected will assist in better characterizing the health economics of this patient population, and potentially allow for comparisons with the literature.
Patients will not receive any questionnaire related to the healthcare resource utilization analyses as the questions are not validated. However, the study team may ask them about it in a neutral way.

# 11. Data Quality Assurance and Control

The Sponsor, in cooperation with the study CRO, will implement and maintain data quality assurance and quality control systems with written SOPs and WIs to ensure that studies are conducted and data are generated, documented (record), and reported in compliance with the protocol, GCP, and applicable regulatory requirements. Monitoring and audits will be conducted during the course of the study for quality assurance purposes.

## 11.1 Data Handling and Record Keeping / Archive

### 11.1.1 Case Report Forms

The study will strictly follow the protocol. If any changes become necessary, they must be laid down in an amendment to the protocol. All amendments of the protocol must be signed by the Sponsor and Investigators and submitted to IEC and RA.

The Investigators will use electronic case report forms (eCRF), one for each enrolled study patient, to be filled in with all relevant data pertaining to the patient during the study. All patients who either entered the study or were considered not-eligible or were eligible but not enrolled into the study additionally have to be documented on a screening log. The Investigator will document the participation of each study patient on the Enrolment Log.

For data and query management, monitoring, reporting, and coding, an internet-based secure data base secuTrial® developed in agreement with the GCP guidelines will be used for this study. It is the responsibility of the Investigator to assure that all data in the course of the study will be entered completely and correctly in the respective data base. Corrections in the eCRF may only be done by authorised persons. In case of corrections the original data entries will be archived in the system and can be made visible. For all data entries and corrections, the date, time of day, and person who is performing the entries will be generated automatically.

eCRFs must be updated so as to correctly reflect the actual patient status throughout the study. Patients must not to be identified in the eCRF by name. Appropriate coded identification (e.g. Patient Number) must be used.
It must be assured that any authorised person, who may perform data entries and changes in the eCRF, can be identified. A list with signatures and initials of all authorised persons will be filed in the ISF and the TMF, respectively.
Documented medical histories and narrative statements relative to the patient's progress during the study will be maintained. These records will also include the following: originals or copies of laboratory and other medical test results (e.g. histology, etc.) which must be kept on file with the individual patient's eCRF.
The Investigators assure to perform a complete and accurate documentation of the patient data in the eCRF. All data entered into the eCRF must also be available in the individual patient file either as print-outs or as notes taken by either the Investigator or another responsible person assigned by the Investigator.

Essential documents must be retained for at least 30 years after the regular end or a premature termination of the respective study according to the Swiss law (Ordinance on Clinical Trials in Human Research (ClinO), Article 45 paragraph 3 and “Federal Act on Medicinal Products and Medical Devices”, Article 40 paragraph 1).
Any patient files and source data must be archived for 30 years.

### 11.1.2 Specification of Source Documents

The following documents are considered source data, including but not limited to:

- SAE worksheets
- Nurse records, records of clinical coordinators, and
- Medical records from other department(s), or other hospital(s), or discharge letters and correspondence with other departments/hospitals, if patient visited any during the study period and the post study period.

Source data must be available at the site to document the existence of the study patients and substantiate the integrity of study data collected. Source data must include the original documents relating to the study, as well as the medical treatment and medical history of the patient.

The following information (at least but not limited to) should be included in the source documents:

- Demographic data (age, sex)
- Inclusion and exclusion criteria details
- Participation in study and signed and dated Informed Consent Forms (ICF)
- Visit dates
- Medical history and physical examination details
- Key efficacy and safety data (as specified in the protocol)
- AEs and concomitant medication
- Results of relevant examinations
- Laboratory printouts
- Dispensing and return of study product details
- Reason for premature discontinuation
- Patient number

### 11.1.3 Record Keeping / Archiving

All study data must be archived for a minimum of 30 years after study termination or premature termination of the clinical trial. Investigators will specify the location of storage of these data in the ISF.
To permit evaluations, audits and/or inspections from IEC, RA or the Sponsor, Investigators agree to keep records, including the identity of all participating patients (e.g. enough information to link records), all original signed informed consent documents, safety reporting forms, source documents and appropriate documentation of relevant correspondence (e.g. letters, meeting minutes, telephone call reports).
The records should be kept by Investigators according to International Conference on Harmonization (ICH) and local regulations. In case an Investigator becomes unable for any reason to continue the retention of study records for the required period, the Sponsor should be prospectively notified. The study records must be transferred to a designee acceptable to the Sponsor. Investigators must receive the Sponsor's written permission before disposing of any records.

#### 11.1.4 Retention of Records

The following are essential study records:

- signed ICFs for all patients
- patient identification numbers (IDs)
- all records of communication between the Investigator and the IEC
- all records of communication between the Investigator and the Sponsor
- a list of all Investigators, Sub-investigators, and personnel
- copies of all financial records related to the study
- copies of all printed components of the eCRFs for all patients
- all source documents
- if applicable, samples of patient's skin cells inside a Swiss GMP biobank

The Investigator will notify the Sponsor if any of these records are lost.

The Investigators will maintain all essential documents for one of the following time periods:

- At least 25 years after the last marketing application in an ICH region and until there are no pending or contemplated marketing applications in an ICH region or
- After at least 25 years have elapsed since the formal discontinuation of clinical development of the investigational product.

The Sponsor will maintain for one of the following time periods:

- Swiss law dictates that samples of patient's skin cells must be stored for at least 30 years within a GMP biobank in Switzerland.

These documents should be retained for a longer period, however, if required by applicable RA or by an agreement with the Sponsor. It is the responsibility of the Sponsor to inform the Investigator/institution as to when these documents no longer need to be retained.
Retention of biological data (e.g. samples of patient's skin cells) will be encrypted. After explicit permission at inclusion of the patients, these samples could be used for further development of skin replacement products. Standards provided in the WMA Declaration of Taipei of ethical considerations regarding health databases and biobanks will be upheld when handling and storing biological data.

### 11.2 Data Management

#### 11.2.1 Data Management System

For data collection, query management, monitoring, reporting and coding a web-based, secure eCRF system, developed in compliance with GCP, FDA (Title 21 CFR Part 11), EMA and CDISC quality standards will be used. The eCRF will be hosted in a dedicated server hired from a professional hosting service provider. The eCRF will be customized for the study needs and its design will be specified in an annotated CRF, a data dictionary, and an edit check plan document. Before the study begins, the eCRF will be tested at user level by authorized Investigators and study coordinators in order to validate its performance. Additionally, tests cases will be designed and run to validate the operation of pre-defined hard edit checks and automatic queries.

#### 11.2.2 Data Security, Access, and Backup

Only authorised Investigators and study coordinators will have access to the eCRF data. Unique and confidential usernames and passwords will be provided to authorised users via encrypted communication. The eCRF will integrate an FDA/EMA-compliant audit trail system to track user logins and logouts, as well as to detect erroneous and/or suspicious access

attempts. Automatic session disconnections after predefined periods of inactivity will be implemented for increased security. The eCRF will be hosted in a secure professional dedicated server carrying out daily data backups.

#### 11.2.3 Data Exports / Transfer for Analysis

eCRF data exports/transfers will be guided by Good Clinical Practice and will involve the automatic generation of dataset files from the eCRF administrator's interface. These data files will then be transferred via encrypted communication to the study research team for analysis. Transfer metrics such as file size, number of datasets, patients per dataset, and number of variables will be used to verify that the data were adequately extracted to the transfer dataset files.

#### 11.2.4 Electronic and Central Data Validation

The eCRF data will be checked for correctness by validity and consistency checks (predefined hard edit checks and automatic queries). Implausible or missing data can be corrected or supplemented following discussion with the Investigator or local study coordinator. All corrections will be tracked and stored by the eCRF integrated audit trail system. The study monitors in charge of reviewing the eCRFs may issue manual queries to request specific data clarifications or corrections, as defined in the Monitoring Plan. eCRF data will be verified with source documents by performing regular monitoring visits to sites.

### 11.3 Staff Training

Prior to enrolment, all clinical study personnel will be trained to ensure adherence to the protocol and assure the highest possible data quality. Training will be led by CRO and the Sponsor at a central location. Training presentations will address informed consent procedures, study operations and protocol requirements, data collection procedures, maintenance of source documentation, eCRF completion and review, routine reporting requirements, data entry and management, and policies and procedures.

### 11.4 Monitoring

Regular monitoring visits at the study sites prior to the start and during the course of the study will help to follow up the progress of the clinical study, to assure utmost accuracy of the data, and to detect possible errors at an early time point. The Sponsor organises professional independent monitoring for the study.

All original data including all patient files, progress notes, and copies of laboratory and medical test results must be available for monitoring. The monitor will review all or a part of the eCRFs and written ICFs. The accuracy of the data will be verified by reviewing the above referenced documents.

The Sponsor collaborates with Julius Clinical, The Netherlands, and Sintesi Research (for study sites in Italy) to ensure regular monitoring. According to Julius Clinical's Monitoring SOP the extent and nature of monitoring activities based on the objective and design of the study will be defined in a study specific Monitoring Plan.

### 11.5 Independent Data Safety Monitoring Board

The Independent Data Safety Monitoring Board (IDSMB) is composed of three experts with appropriate knowledge of this patient population, biostatistics, and ethics. They are all independent of the study. They will perform a safety and efficacy evaluation, in accordance with the study specific SOP, for the protection of the study patients. Activity, list of members

and organization will be detailed in an IDSMB charter that will be signed and validated before the activity will start. The study may be stopped or continued by the Sponsor according to the recommendations of the IDSMB. The member of the IDSMB are:

**Table 12:** Overview IDSMB members as per date of this protocol

| **Name** | **Affiliation / Address** | **Phone, Email** |
|---|---|---|
| Prof. Dr.<br>Christoph Berger | Infectious Diseases<br>University Children's Hospital Zurich<br>Department of Paediatrics<br>Steinwiesstrasse 75<br>8032 Zurich, Switzerland | Phone: +41 44 266 72 50<br>Email: christoph.berger@kispi.uzh.ch |
| Dr. med.<br>Abdul R. Jandali | Hand and Plastic Surgery<br>Kantonsspital Winterthur<br>Brauerstrasse 26<br>Postfach 834<br>8401 Winterthur, Switzerland | Phone: +41 52 266 24 08<br>Email: abed.jandali@ksw.ch |
| Prof. Dr. med.<br>Holger Bannasch | ÄD Klinik für Plastische-, Hand-, und Ästhetische Chirurgie Kliniken Donaueschingen<br>Sonnhaldenstr. 2<br>78166 Donaueschingen, Germany | Phone: +49 771 88-0<br>Email: holger.bannasch@sbk-vs.de |

## 11.6 Audits and Inspections

Quality assurance audits/inspections of the study may be conducted by an independent auditor upon Sponsor decision, RA or IEC, respectively. Monitoring of data quality will also be ensured by the Sponsor. The quality assurance auditor/inspector will have access to all medical records, the Investigator's study related files and correspondence, and the informed consent documentation that is relevant to the clinical study.
The Investigator will allow the persons being responsible for the audit or the inspection to have access to the source data/documents and to answer any questions arising. All involved parties will keep the patient data strictly confidential.

## 11.7 Processing of Personal Data

The Parties shall handle all Personal Data in accordance with the General Data Protection Regulation (GDPR) and with any other applicable data protection laws in relation to the processing of Personal Data. In Switzerland, the same principles will be applied as in the EU. The Sponsor's appointed Data Protection Officer and EU Data Representative is:

Hemex Germany GmbH
Pascal Winnen
Marie-Curie-Strasse 8
79539 Lörrach
Germany
Phone: +49 1512 568 48 92
E-mail: dpo@hemex-germany.de

### 11.7.1 Study Patients' Personal Data

a. Role and qualification of the Parties

Sponsor is subject to the rights and obligations as "data controller" set forth under the GDPR in relation to the processing of personal data for the purpose of conducting the Study in

accordance with the Protocol. In that respect Sponsor shall be considered as data controller of all Personal Data processed for study purposes.

The investigational sites are subject to the rights and obligations as “data processor” set forth under the GDPR in relation to the processing of personal data for the purpose of conducting the study in accordance with the Protocol (Pursuant to Article 28.3 GDPR Sponsor and each of the investigational sites conclude a Data Processing Agreement).

The investigational sites are also subject to the rights and obligations as a separate “data controller” set forth under the GDPR in relation to the processing of personal data of their patients and staff for purposes other than conducting the study. In particular, the investigational sites remain data controller of the data contained in their patients’ medical records for the purposes of providing medical care to their patients and for academic research purposes, and of the data contained in the employee’s records related to education and training required by Sponsor to assess qualification for participation in the study.

b. Cooperation

Both Sponsor and the investigational sites shall implement appropriate technical and organizational measures to meet the requirements of the GDPR.

If either Party becomes aware of a personal data breach, that Party shall promptly notify the other Party/ies without undue delay and, where feasible, within 72 hours. In such a case Parties will fully cooperate with each other to remedy the personal data breach, fulfil the notification obligations timely and cure the damages. A personal data breach refers to a personal data breach as meant in articles 33 and 34 of the GDPR.

In order to protect the identity of the study patients vis-à-vis the Sponsor, the Sponsor and the investigational sites agree that, as between them, the data protection officer of the investigational sites (as identified in the Data Processing Agreement) will act as an intermediary to manage and resolve requests from a study patient, as the case may be, to access, modify, transfer, block, or delete of her/his personal data, and that he/she will contact the data protection officer of the Sponsor (as identified in the Data Processing Agreement) in such case.

The investigational sites acknowledge that in order to maintain the integrity of Study results, the ability to amend, modify, or delete Personal Data may be limited by Sponsor, in accordance with Applicable Laws.

### 11.7.2 Study Staff

Prior to and during the course of the study, the Sponsor may request to process personal data of study staff of the investigational sites, including from the investigational sites’ Investigators, Sub-investigators, other investigational site staff, or personnel involved in the conduct of the study.

The Sponsor as data controller for the processing of such study staff’s personal data for study purposes is responsible for supplying such investigational sites’ study staff with the necessary information regarding the collection of their personal data pursuant to GDPR.
The investigational sites through the Investigator will assist the Sponsor in providing such information to the investigational sites’ study staff upon request from the Sponsor.

The supplied information should address, where applicable, the transfer of personal data to countries outside the European Economic Area, including without limitation the United States,

possibly not providing an adequate level of data protection, in which case the supplied information should also address the steps taken by the Sponsor to ensure that the personal data remains secure.

The European Commission has taken an adequacy decision for Switzerland, meaning that it has decided that Switzerland ensures an adequate level of data protection. Therefore, the transfer of personal data from investigational sites in the European Economic Area to Switzerland shall not require any specific authorisation.

The purposes for which the personal data of the investigational sites' study staff are processed by the Sponsor shall be detailed in the supplied information and may include:
a. the conduct and interpretation of the study;
b. review by governmental or regulatory agencies, the Sponsor, and its affiliates;
c. satisfying legal or regulatory requirements;
d. publication on www.clinicaltrials.gov and other websites and databases that serve a comparable purpose;
e. upon request of individual patients and doctors, provision of information regarding the study to individual patients and doctors who may be interested in participating in the clinical study at the investigational site(s);
f. storage in Sponsor's databases for use in selecting sites in future clinical studies.

The supplied information should also include the right to access, modify, rectify, or remove their personal data from such processes as well as the retention period of the data by the Sponsor.

## 11.8 Clinical Laboratory Evaluation

Clinical laboratory values will be evaluated for each laboratory parameter by patient. Abnormal laboratory values will be identified as those outside (above or below) the normal range. An Investigator must review the lab values and document the clinical significance, and whether any abnormal values are expected in the context of the severe burn patient. Reference (normal) ranges for laboratory parameters will be included in the CSR for this protocol.

## 11.9 Vital Signs

Vital sign values (BP, HR, and temperature) will be evaluated on an individual basis by patient. Abnormal vital sign values will be identified as those outside (above or below) the reference range. It is under Investigator responsibility to assess their clinical significance.

# 12. Ethical and Regulatory Aspects

The procedures set out in this study protocol, pertaining to the conduct, evaluation, and documentation of this study, are designed to ensure that the Sponsor and Investigators abide by GCP as described in the ICH Harmonized Tripartite Guideline E6 (R2): Consolidated Guideline, and for US Investigators, 21 CFR Parts 50, 54, 56, and 312. Compliance with these guidelines and regulations also constitutes compliance with the ethical principles described in the current revision of the Declaration of Helsinki. The study will also be carried out in keeping with local legal and regulatory requirements.

## 12.1 Study Registration

The study is registered (NCT03227146) in the international trial registry provided by the U. S. National Institutes of Health (http://www.clinicaltrials.gov). If required, the study will additionally be registered in local study registers of the participating countries.

## 12.2 Study Categorization

The study with EHSG-KF has been classified as a clinical trial with IMP Category C according to ClinO (Swiss law) as the Advanced-Therapy Medicinal Product is not authorised in Switzerland or any other country.

## 12.3 Independent Ethics Committee (IEC)

Before the study is conducted, the protocol, the proposed patient information and consent forms, as well as other study-specific documents, will be submitted to properly constituted Independent Ethics Committees (IECs) for approval in each country. The reporting duties and allowed time frames will be respected. Any amendment to the protocol must be approved as well.

The decision of IECs concerning the conduct of the study will be provided with a written and dated approval/favourable opinion to the Investigators before commencement of the study. The clinical study can only begin in a given country once approval from all required authorities has been received. Any additional requirements imposed by the authorities shall be implemented.
After IEC approval, any changes will require a formal amendment.

No substantial changes will be made to the protocol without prior Sponsor, IEC and RA approval, except where necessary to eliminate apparent immediate hazards to study patients. Changes that do not affect patient safety or data integrity are classified as administrative changes and generally do not require IEC approval. If ethical aspects are concerned, the IEC must be informed and if necessary, approval sought prior to implementation. Ethical approval of administrative change will be obtained if required by local/site IEC.

Premature study end, interruption, regular end, and the CSR and Addendum to the CSR shall be timely reported to each IEC in accordance with applicable national requirements. Amendments are reported according to chapter 12.10.

## 12.4 Regulatory Authority (RA)

The Sponsor will obtain approval from RAs in each participating country, in agreement with local legal requirements, before the start of the clinical trial. Reporting will be done within the allowed time frames.
Premature study end, interruption, regular end, and the CSR and Addendum to the CSR shall be timely reported to each RA in accordance with national requirements.

## 12.5 Patient Privacy and Confidentiality

During this study, patient medical findings and personal information will be collected and documented from their personal medical records at the study site(s) and stored electronically. The data relevant for the clinical study will be coded (no names or initials will be used, but a code of numbers not comprehensible to outsiders), analysed and, if necessary, passed on to third parties. Decryption will only be performed in the case of medical emergencies, and under the conditions required by law, upon inspection by RA or the Sponsor's representative to verify the proper conduct of the clinical trial.

Data generation, transmission, archiving, and analysis of personal data within this study, strictly follows the current Swiss and European legal requirements for data protection. Prerequisite is the voluntary approval of the patient given by signing the informed consent prior to the start of their participation in the clinical trial.
The investigational site(s) affirm and uphold the principle of the patient's right to privacy and that they shall comply with applicable privacy laws. The investigational site(s) will maintain a separate list with at least the initials, the patient's study numbers, names, addresses and telephone numbers and store this for the longest period allowed by his/her own institution and, in any case, until further communication from the Sponsor.

Anonymity of the patients shall be guaranteed when presenting the study related data at scientific meetings or publishing them in scientific journals. However, individual patient medical information obtained as a result of this study is considered confidential and disclosure to third parties is prohibited.

For data verification purposes, properly authorized representatives acting on behalf of the Sponsor (the study monitor, a RA (e.g. Swissmedic, Health Care Inspectorate), or an IEC) may require direct access to parts of the medical records relevant to the study, including the patients' medical history.

Personal medical information will always be treated as confidential. Such medical information may be given to the patient's personal physician or to other appropriate medical personnel responsible for the patient's welfare if the patient has given his/her written consent to do so.

## 12.6 Non-Disclosure

All study documents are provided by the Sponsor in confidence to the investigational site(s) and its appointed staff. None of this material may be disclosed to any party not directly involved in the study without written permission from the Sponsor.

## 12.7 Declaration of Interest

Financial arrangements between the Sponsor and Study Sites / Investigators are described in detail in separate Clinical Trial Agreements and shall be designed as to not bias the data collection.
Prof. Dr. Ernst Reichmann (Sponsor representative until 31. December 2020), Prof. Dr. med. Martin Meuli (PI at University Children's Hospital Zurich, Switzerland, until 30. June 2020), and Prof. Dr. med. Clemens Schiestl (Coordinating Investigator) hold equity interest in the commercial organisation CUTISS AG. CUTISS AG is a Swiss life science start-up company (incorporated in March 2017) and a spin-off company of the University of Zurich, Switzerland. The aim of CUTISS AG is to further develop the personalised skin graft EHSG-KF, fund raising and coordination of the EHSG-KF product development towards scale-up and market access. CUTISS AG has no sales and no return as per date of this document. Best case scenario, first sales are planned as for 2023.
Contact: CUTISS AG, www.cutiss.swiss, clinicaltrials@cutiss.swiss

### 12.7.1 Oversight Committee (OC)

In order to completely rule out potential bias or conflict of interest of the shareholders, an oversight committee (OC) composed of three independent, international, surgeons, burn experts, has been set up. The members of the OC will overlook the full progress and development of the studies, having full access to data and meeting regularly. The OC is free to communicate to the Sponsor's representative any suspect of potential bias or conflict of interest. The member of the OC committee are:

**Table 13:** Overview OC members as per date of this protocol

| Name | Affiliation / Address | Phone, Email |
|---|---|---|
| Prof. Dr. med. Hans-Oliver Rennekampff | Rhein-Maas Klinikum<br>Klinik für Plastische Chirurgie, Hand- und Verbrennungschirurgie<br>Mauerfeldchen 25<br>52146 Würselen, Germany | Phone: +49 2405 62 33 13<br>Email: hans-oliver.rennekampff@rheinmaasklinikum.de |
| Univ. Prof. Dr. med. Peter M. Vogt | Medizinische Hochschule Hannover<br>Schwerbrandverletztenzentrum der Klinik für Plastische-, Hand und Wiederherstellungschirurgie<br>Carl-Neuberg-Str. 1<br>30625 Hannover, Germany | T +49 511 532 88 60<br>Email: phw@mh-hannover.de |
| Prof. Dr. med. Matthias Baumgartner | Abteilung für Stoffwechselkrankheiten<br>Universitäts Kinderspital Zürich<br>Steinwiesstrasse 75<br>8032 Zurich, Switzerland | T +41 44 266 77 22<br>Email: matthias.baumgartner@kispi.uzh.ch |

Additionally, an international Dutch CRO, Julius Clinical (JC) has been engaged to perform monitoring, together with the Italian CRO Sintesi Research for study sites in Italy.

## 12.8 Patient Information and Informed Consent

It is the responsibility of the Investigator to obtain written Informed Consent from patients. All consent documentation must be in accordance with applicable regulations and GCP.

Prior to study entry, the Investigator, or a person designated by the Investigator will explain the nature, purpose, benefits and risks of participation to each patient and/or parents/legally authorized representative. The Sponsor will provide a patient information sheet and a consent

form describing this study and providing sufficient information to make an informed decision about the patient's participation in this study.
Before any study specific procedures or assessments, the patient and/or parents/legally authorized representative is requested to sign the patient Informed Consent Form (ICF). Each signature must be dated by each signatory and the informed consent and any additional patient-information form retained by the Investigator as part of the study records.
The Investigator has to be attentive for any sings of verbal or nonverbal resistance to participate in the study on the part of the minor and is responsible for initiation of an early study withdrawal whenever these arise, even if the legal representative is in favour of continuing the study.

The Investigator will keep the original consent/assent and copies of the signed ICF documentation (consent form or patient information sheet and the consent form, as applicable) must be given to the patient and/or the parents/legally authorized representative. If applicable, it will be provided in a certified translation of the patient's language. Signed consent forms must remain in each patient's study file and must be available for verification by study monitors at any time.

Each PI will provide the Sponsor with a copy of the IRB/IEC approved consent forms, and a copy of the IRB's written approval, prior to the start of the study. Additionally, if the IRB/IEC required modification of the sample patient information and consent document provided by the Sponsor, the documentation supporting this requirement must be provided to the Sponsor.

A patient's participation in the study is completely voluntary. The Investigator will emphasize that a patient has the right to withdraw consent at any time during the study without penalty or loss of benefits to which the patient is entitled. When developmentally appropriate, the patient will be informed about the study to the extent compatible with the patient's understanding and asked to give assent. During all phases of the study, the patient and parents/legally authorized representative have the final say in what happens to their data. If a patient withdraws from further participation, they have the right to refuse the future inclusion of their individually collected data. This means that we, the researchers, will have to explicitly ask for the patients' approval for further data inclusion after withdrawal. Assent should be obtained and documented in accordance with all federal, state, and local laws. All data collected up to the point of withdrawal will be used for coded data analysis.

Should a protocol amendment become necessary, the patient consent form and patient information form may need to be revised to reflect the changes to the protocol. It is the responsibility of the Sponsor to ensure that an amended consent form is reviewed and has received approval / favourable opinion from the IRB/IEC, RA and/or other agencies in accordance with local laws and regulations, and that it is signed by all patients subsequently entered in the study and those currently in the study, if affected by the amendment. Patients and their legal representatives who have completed the study should be informed of any new information that may impact their well-being.

The Investigator should, with the consent of the patient, inform the patient's primary physician about their participation in the clinical trial.

In accordance with the Declaration of Helsinki, if patients are physically or mentally incapable of giving consent, as can be the case with unconscious, intubated severe burn patients, and the parents/legally authorized representative is not available to provide consent, the biopsy will be obtained from the patient prior to obtaining informed consent, with the approval of a 'neutral' physician who is not involved in the study, so as not to delay manufacturing of the study product. In this case, signing by the neutral physician of the corresponding confirmation form

is obligatory prior to taking the biopsy. Additionally, subsequent provision of the patient and/or his parents/legally authorized representative with the study information and obtaining their subsequent consent as early as possible is mandatory. In the case that these patients decline further participation in the study, the data collected at that point will not be eligible for data inclusion. The manufacturing process requires approximately 32 days and therefore it is critical that the manufacturing is initiated as soon as possible.
Of note, in severe burn patients with limited STSG donor sites, partial thickness biopsies are obtained acutely for the manufacturing of cultured epithelial autograft as part of the standard of care treatment. The additional risk of taking a 4 $cm^2$ larger biopsy would be negligible to a patient with a severe burn.

## 12.9 Study Site Discontinuation

Should conditions requiring further clarification arise before the decision to proceed with or terminate the study can be reached, the study will be suspended until the situation has been resolved.

The Sponsor has the right to terminate this study and remove all study material from the site at any time. Premature termination of this study may occur because of change in opinion of the IRB/IEC, RA decision, safety problems related to the IMP or at the discretion of the Sponsor. Sponsor has the right to discontinue the development of EHSG-KF at any time.

## 12.10 Modifications of the Protocol

All amendments to the protocol must be documented in writing, reviewed, and approved by the Investigator and the Sponsor, and submitted to the RA and/or IEC for approval prior to initiation. If the protocol amendment substantially alters the study design or potential risk to the patient, new written informed consent for continued participation in the study must be obtained from each patient.
When an amendment is created in order to address life threatening safety concerns or other non-life threatening, but significant, safety concerns, the protocol changes will be implemented prior to the approval of the RA and/or IEC. The RA and/or IRB/IEC will be promptly informed thereafter.

## 12.11 Deviations from Study Protocol

No deviation may be made from the protocol unless an amendment has been agreed to in writing by both the Investigator and the Sponsor and approved by the RA and/or IRB/IEC. Investigative sites will contact the medical monitor to request clarifications regarding any aspect of the clinical study or eligibility of patients.
When an emergency occurs that requires a departure from the protocol for an individual, a departure will be only for that patient. The Investigator or other physician in attendance in such an emergency will, if circumstances and time permit, contact the Sponsor or their representatives, immediately by telephone. Such contacts will be made as soon as possible to permit a decision as to whether or not the patient (for whom the departure from protocol was affected) is to continue in the study. The source documents will completely describe the departure from the protocol and state the reasons for such departure. In addition, the IRB/IEC will be notified in writing of such departure from protocol.

## 12.12 Good Clinical Practice (GCP) Compliance

The study will be carried out in accordance with principles enunciated in the current version of the Declaration of Helsinki, the guidelines of GCP issued by the International Conference on Harmonization (ICH), and RA's requirements in Switzerland, The Netherlands, Italy and all further countries involved in this study. IRB/IEC and RA will receive the annual DSUR and will be informed about study stop/end in agreement with local requirements.
In addition, this study will adhere to all local regulatory requirements.

It is the Investigator's responsibility to ensure that adequate time and appropriate resources are available at the study site prior to commitment to participate in this study. The Investigator should also be able to estimate or demonstrate a potential for recruiting the required number of suitable patients within the agreed recruitment period.
The Investigator will maintain a list of appropriately qualified persons to whom the Investigator has delegated significant study-related tasks. An up-to-date copy of the curriculum vitae for the Investigator, sub-Investigator(s) and essential study staff will be provided to the Sponsor (or designee) before starting the study.

If the patient has a primary physician or a general practitioner, the Investigator should, with the patient's consent, inform them of the patient's participation in the study.

Before initiating a study, the Investigator/institution must have obtained written and dated approval/favourable opinion from the IRB/IEC for the study protocol/amendment(s), written informed consent forms, any consent form updates, patient emergency study contact cards, patient recruitment procedures (e.g. advertisements), any written information to be provided to patients and a statement from the IRB/IEC that they comply with GCP requirements. The IRB/IEC approval must identify the protocol version as well as the documents reviewed.

After IRB/IEC approval, changes will require a formal amendment. Once the study has started, amendments should only be made in exceptional circumstances. Changes that do not affect patient safety or data integrity are classified as administrative changes and generally do not require ethical approval. If ethically relevant aspects are concerned, the IRB/IEC must be informed and, if necessary, approval sought prior to implementation. Ethical approval of administrative changes will be obtained if required by local/site IRB/IEC.

All protocol deviations will be recorded on the Protocol Deviation Form. Documentation will include a description of the event, corrective measures taken, reporting duties, and the final disposition of the event.

## 12.13 Study Completion

The IEC and RA in each participating country needs to be notified about the end of the study or early termination of the study (national/global notification).
After the study completion, the Investigator will ensure that additional care is provided to each participating patient if required.

## 13. Publication and Dissemination Policy

Prior to any submission, all manuscripts/abstracts have to be presented to the Sponsor for review and comments. After the statistical analysis of the study, the Sponsor and the Investigators will make every endeavour to publish the data in a medical or life science journal according to DOH Seoul October 2008, item B 30. There are no limitations with regard to publication and/or public disclosure of study data, provided patient anonymity is maintained, or, a written consent regarding publication by the patient and/or patient’s parents/legally authorized representative is obtained.

# 14. Funding and Support

This study is funded by:

Wyss Zurich
ETH Zurich / University of Zurich
Weinbergstrasse 35
8092 Zurich, Switzerland

Project name: "denovoSkin"
Email: info@wysszurich.ch
Phone: +41 44 633 89 79

and

CUTISS AG
Grabenstrasse 11
8952 Schlieren, Switzerland

Email: clinicaltrials@cutiss.swiss
Phone: +41 44 244 36 60

# 15. Insurance

A clinical study insurance policy, in accordance with pertinent regulatory requirements, will be provided for the study in each country and sent to corresponding IECs and RAs. This policy will be issued and funded by the Sponsor or its delegate in the respective country.

Any damage developed in relation to study participation is covered by this insurance. So as not to forfeit their insurance coverage, the patients themselves must strictly follow the instructions of the study personnel. Patients must not be involved in any other medical treatment without permission of the PI (emergency excluded).
Medical emergency treatment must be reported immediately to the Investigator. The Investigator must also be informed instantly, in the event of health problems or other damages during or after the course of study treatment.
The Investigator will allow delegates of the insurance company to have access to the source data/documents as necessary to clarify a case of damage related to study participation. All involved parties will keep the patient data strictly confidential.
A copy of the insurance certificate will be placed in the Investigator's Site File.

CUTISS personalized skin

# 16. Appendix

## 16.1 Appendix 1 - Study Flow Sheet

| Study Schedule TBRU-dS-BA-PIIb | | | |
|---|---|---|---|
| Procedure | Visit | Day | Details of Procedure |
| **Pre-Treatment Phase** | | | |
| Screening | 1 | N/A | • Screening of hospital inpatients, informed consent, medical history, demographic data, physical examination<br>• Skin defect will be excised and covered temporarily (e.g. using donor skin)<br>• Screening laboratory, routine laboratory, pregnancy test (if applicable) |
| Biopsy | 2 | N/A | • Checking of inclusion- and exclusion criteria<br>• Inpatient setting (main operating room or procedure room)<br>• Split-thickness skin biopsy (full-thickness after previous confirmation of sponsor) (Biopsy Nr. 1) |
| Manufacturing | - | - | • Manufacturing of EHSG-KF at the manufacturing site |
| **Treatment Phase** | | | |
| Transplantation | 3 | day 0 | • Checking of inclusion- and exclusion criteria, routine laboratory, physical examination<br>• Surgical wound bed preparation; facultative biopsies of the wound grounds before and after preparation (Biopsies Nr. 2-5)<br>• Wound swabs and clinical evaluation for signs of infection<br>• Grafting of EHSG-KF/ STSG |
| 1. Graft Check | 4 | 6-10 | • Routine laboratory, dressing change<br>• Assessment of local infection incl. wound swabs<br>• Assessment of % graft take |
| 2. Graft Check | 5 | 21 ± 2 | • Dressing change<br>• Assessment of local infection incl. wound swabs<br>• Assessment of % epithelialization |
| 3. Graft Check | 6 | 28 ± 3 | • Dressing change<br>• Assessment of local infection incl. wound swabs<br>• Assessment of % epithelialization<br>• Assessment of wound closure<br>• Assessment of ratio of covered surface area to biopsy site / donor site surface area |
| **Post-Treatment Phase** | | | |
| Follow-up | 7 | 60 ± 3 | • Clinical evaluation in hospital (when patient remains an inpatient) or in routine outpatient clinic<br>• Assessment of % epithelialization<br>• Assessment of wound closure |
| Follow-up | 8 | 90 ± 5 | • Clinical evaluation in hospital (when patient remains an inpatient) or in routine outpatient clinic<br>• Assessment of % epithelialisation<br>• Assessment of wound closure<br>• Cutometer and DMS ColorMeter assessment<br>• POSAS and QOL questionnaires<br>• Optional representative biopsies of each study area (Biopsies Nr. 6-7) |
| Follow-up | 9 | 6m ± 10 | • Clinical evaluation in hospital (when patient remains an inpatient) or in routine outpatient clinic<br>• Assessment of epithelialisation<br>• Cutometer and DMS ColorMeter assessment<br>• POSAS and QOL questionnaires<br>• Health resource utilization |
| Follow-up | 10 | 1y ± 30 | • Clinical evaluation in routine outpatient clinic<br>• Physical examination<br>• Cutometer and DMS ColorMeter assessment<br>• POSAS and QOL questionnaires<br>• Health resource utilization<br>• Optional representative biopsies of each study area (Biopsies Nr. 8-9)<br>• Surface area measurement |
| **Long-Term Follow-up** | | | |
| Long-Term Follow-up | 11 | 2y ± 30 | • Clinical evaluation in routine outpatient clinic<br>• Cutometer and DMS ColorMeter assessment<br>• POSAS and QOL questionnaires<br>• Health resource utilization |
| Long-Term Follow-up | 12 | 3y ± 30 | • Clinical evaluation in routine outpatient clinic<br>• Cutometer and DMS ColorMeter assessment<br>• POSAS and QOL questionnaires<br>• Surface area measurement |
| **General** | | | |
| | | | • Assessment of vital signs at all visits from visit 1 on<br>• Documentation of concomitant therapy at all visits from visit 1 on<br>• Assessment of Adverse Events at all visits from visit 2 on<br>• Photographic documentation at all visits from visits 3 on |

## 16.2 Appendix 2 – Addendum 1 for Sub-Study

# ADDENDUM 1

# SUB-STUDY TO PROTOCOL TBRU-dS-BA-PIIb-STUDY

**Title:** **An open-label, prospective, non-randomised, non-controlled study to evaluate the survival of the adult and adolescent patients with deep partial and full-thickness burns in a life threatening situation treated with EHSG-KF, and to evaluate the safety and efficacy of EHSG-KF**

**Sites:** **a) Azienda Ospedaliera di Rilievo Nazionale Antonio Cardarelli (Italy), b) Santobono Napoli (Italy), and c) Azienda Ospedale Università Padova (Italy)**

CUTISS personalized skin

## Table of Contents
## Sub-Study to Protocol TBRU-dS-BA-PIIb Study


**SPONSOR PROTOCOL APPROVAL SUBSTUDY-** as per main protocol

**COORDINATING INVESTIGATOR AGREEMENT SUBSTUDY-** as per main protocol

**PRINCIPAL INVESTIGATOR AGREEMENT SUBSTUDY-** as per main protocol

**STUDY ADMINISTRATIVE STRUCTURE**

**ABBREVIATIONS AND DEFINITIONS** - as per main protocol

**1. INTRODUCTION AND RATIONALE SUB-STUDY**
1.1 BACKGROUND AND RATIONALE
1.1.1 Non-Clinical Summary
1.1.2 Clinical Summary
1.1.3 Rationale for the Study Product
1.2 INVESTIGATIONAL MEDICINAL PRODUCT
1.3 BENEFIT / RISK ASPECTS
1.3.1 Benefits of EHSG-KF
1.3.2 Risks of EHSG-KF
1.3.3 Risk mitigation strategies
1.4 DOSE RATIONALE
1.5 JUSTIFICATION OF CHOICE OF STUDY POPULATION

**2. SUB-STUDY OBJECTIVES SUB-STUDY**
2.1 PRIMARY OBJECTIVE
2.2 SECONDARY OBJECTIVES

**3. INVESTIGATIONAL MEDICINAL PRODUCT INFORMATION SUB-STUDY**
3.1 PRODUCTION OF IMP
3.2 PACKAGING, LABELLING, SUPPLY, ACCOUNTABILITY, DESTRUCTION
3.3 EXPERIMENTAL INTERVENTION
3.4 CONTROL INTERVENTION (CURRENT GOLD STANDARD)
3.5 RANDOMISATION
3.6 COMPLIANCE WITH STUDY INTERVENTION
3.7 CONCOMITANT MEDICATION

**4. SELECTION OF STUDY POPULATION SUB-STUDY**
4.1 INCLUSION CRITERIA
4.2 EXCLUSION CRITERIA
4.2.1 Contraception Methods
4.3 WITHDRAWAL / DISCONTINUATION OF PATIENTS
4.4 DATA COLLECTION AND FOLLOW-UP FOR WITHDRAWN PATIENTS

personalized skin


**5. STUDY DESIGN AND COURSE OF THE SUB-STUDY**
- 5.1 STUDY CENTRES
- 5.2 STUDY DESIGN
- 5.3 STUDY PROCEDURES
- 5.4 END OF STUDY

**6. ENDPOINTS SUB-STUDY**
- 6.1 PRIMARY ENDPOINT
- 6.2 SECONDARY AND EXPLORATORY ENDPOINTS
- 6.3 DURATION OF PATIENT PARTICIPATION

**7. STUDY ASSESSMENT AND PROCEDURES SUB-STUDY**

**8. ASSESSMENTS OF ENDPOINTS SUB-STUDY**
- 8.1 PRIMARY ENDPOINT
- 8.2 SECONDARY AND EXPLORATORY ENDPOINT

**9. SAFETY SUB-STUDY**

**10. STATISTICAL ANALYSES SUB-STUDY**

**11. DATA QUALITY ASSURANCE AND CONTROL SUB-STUDY**

**12. ETHICAL AND REGULATORY ASPECTS SUB-STUDY**

**13. PUBLICATION AND DISSEMINATION POLICY SUB-STUDY**

**14. FUNDING AND SUPPORT SUB-STUDY**

**15. INSURANCE SUB-STUDY**

## Study Administrative Structure- SUB-STUDY

| Structure | Details |
|---|---|
| **Sponsor** | CUTISS AG<br>Grabenstrasse 11<br>8952 Schlieren, Switzerland<br>Phone: +41 44 244 36 60<br>Email: clinicaltrials@cutiss.swiss |
| **Coordinating Investigator** | Prof Dr. med. Clemens Schiestl<br>Director, Paediatric Burn Centre<br>Department of Surgery<br>University Children’s Hospital Zurich<br>Steinwiesstrasse 75<br>8032 Zurich, Switzerland<br>Phone: +41 44 266 74 13<br>Email: Clemens.Schiestl@kispi.uzh.ch |
| **Statistician** | Jean-Christophe Lemarie<br>Director of Statistics<br>EFFI-STAT<br>22, rue du Pont Neuf<br>75001 Paris, France<br>Phone: +33 15 534 96 00<br>Email: jean-christophe.lemarie@effi-stat.com |
| | Julius Clinical<br>Broederplein 41-43<br>3703 CD Zeist, The Netherlands<br>Phone: +31 (0)30 656 99 00<br>Fax Number: +31 30 656 99 90<br>Email: info@juliusclinical.com |
| **Monitoring Institution** | Julius Clinical<br>Broederplein 41-43<br>3703 CD Zeist, The Netherlands<br>Phone: +31 (0)30 656 99 00<br>Fax Number: +31 30 656 99 90<br>Email: linda.roest@juliusclinical.com; tbru.safety@juliusclinical.com |
| | Sintesi Research Srl<br>C.so di P.ta Romana, 132<br>20122 Milano, Italy<br>Phone:  +39 348 185 20 52<br>Fax: +39 0297374301<br>p.desimoni@sintesiresearch.com |

| | |
|---|---|
| **Independent Data Safety Monitoring Board** | Prof. Dr. Christoph Berger<br>Paediatrician, Infectious Diseases<br>University Children's Hospital Zurich<br>Department of Paediatrics<br>Steinwiesstrasse 75<br>8032 Zurich, Switzerland<br>Phone: +41 44 266 72 50<br>Email: christoph.berger@kispi.uzh.ch |
| | Dr. med. Abdul R. Jandali<br>Director, Hand and Plastic Surgery<br>Kantonsspital Winterthur<br>Brauerstrasse 15<br>Postfach 834<br>8401 Winterthur, Switzerland<br>Phone: +41 52 266 24 08<br>Email: abed.jandali@ksw.ch |
| | Prof. Dr. med. Holger Bannasch<br>ÄD Klinik für Plastische-, Hand-, und Ästhetische Chirurgie Kliniken Donaueschingen<br>Sonnhaldenstr. 2<br>78166 Donaueschingen, Germany<br>Phone: +49 771 88-0<br>Email: holger.bannasch@sbk-vs.de |
| **Oversight Committee** | Univ. Prof. Dr. med. Peter M. Vogt<br>Direktor<br>Medizinische Hochschule Hannover<br>Schwerbrandverletztenzentrum der Klinik für Plastische-, Hand und Wiederherstellungschirurgie<br>Carl-Neuberg-Str. 1<br>30625 Hannover, Germany<br>Phone: +49 511 532 - 0<br>Fax: +49 511 532 - 8890<br>Email: phw@mh-hannover.de |
| | Prof. Dr. med. Hans-Oliver Rennekampff<br>Rhein-Maas Klinikum<br>Klinik für Plastische Chirurgie, Hand- und Verbrennungschirurgie<br>Mauerfeldchen 25<br>52146 Würselen, Germany<br>Phone: +49 2405 62 33 13<br>Email: hans-oliver.rennekampff@rheinmaasklinikum.de |
| | Prof. Dr. med. Matthias Baumgartner<br>Abteilung für Stoffwechselkrankheiten Universitäts-Kinderspital Zurich<br>Steinwiesstrasse 75<br>8032 Zurich, Switzerland<br>Phone: +41 44 266 77 22<br>Email: matthias.baumgartner@kispi.uzh.ch |

# 1. INTRODUCTION AND RATIONALE SUB-STUDY

## 1.1 BACKGROUND AND RATIONALE

In patients with extensive burns, clinicians and researchers face the challenge of providing definitive burn wound coverage in the setting of limited skin graft donor sites. The development of alternatives to autologous split-thickness skin grafts has, thus, become a cornerstone in burn research[5–11] and could prove to be life-saving.

The current main protocol allows adults and adolescents to be included according to the inclusion and exclusion criteria and potentially would exclude patients with extensive and life-threatening burn wounds based on these criteria. In such life-threatening situations, it would not be beneficial and ethical to limit the number of potential grafts with EHSG-KF to those foreseen by the main study protocol, neither would it be feasible to include a control arm. On the other hand, it could be worthwhile studying the life-saving efficacy of EHSG-KF in such patients when no other alternative treatment is applicable.

To allow access and benefit of treatment with the EHSG-KF, patients ≥ 12 years of age and in a life-threatening situation can be enrolled in this sub-study with the final benefit of survival. This sub-study will permit:

- the transplantation of >2 EHSG-KF to increase the potential benefit of survival,
- the exclusion of a control arm (as per main protocol TBRU-dS-BA-PIIb-Study).

### 1.1.1 Non-Clinical Summary

Please refer to main protocol TBRU-dS-BA-PIIb-Study

### 1.1.2 Clinical Summary

Please refer to main protocol TBRU-dS-BA-PIIb-Study

### 1.1.3 Rationale for the Study Product

Please refer to main protocol TBRU-dS-BA-PIIb-Study

## 1.2 INVESTIGATIONAL MEDICINAL PRODUCT

Please refer to main protocol TBRU-dS-BA-PIIb-Study

## 1.3 BENEFIT / RISK ASPECTS

Please refer to main protocol TBRU-dS-BA-PIIb-Study
AND
By allowing a higher number of EHSG-KF grafts to be applied to patients in a life-threatening situation with no alternative treatments and extensive burn wounds, this sub-study will provide the additional benefit of survival, that would be at risk in the absence of these EHSG-KF grafts, if no other treatment options are feasible.

## 1.4 DOSE RATIONALE

Please refer to main protocol TBRU-dS-BA-PIIb-Study
AND
With the successful completion of the phase I study with, now a 5 year follow up completed, and the reassurance that there are no major safety concerns with the use of EHSG-KF, it is important to study the effect of EHSG-KF when grafted over larger areas when possible and to have patients in severe conditions to benefit from wound coverage by EHSG-KF with the aim of preserving life otherwise at risk.

## 1.5 JUSTIFICATION OF CHOICE OF STUDY POPULATION

The current study will be conducted in adult and adolescent patients ≥ 12 years with acute burns, while a separate parallel phase IIb study will be conducted in children < 12 years. It is our goal to elucidate the safety and efficacy of EHSG-KF for a broad patient population since patients of all ages may benefit from a bio-engineered skin analogue in the setting of severe burns.

## 2. SUB-STUDY OBJECTIVES SUB-STUDY

### 2.1 Primary Objective
To evaluate the survival of the adult and adolescent patients with deep partial and full-thickness burns in a life- threatening situation treated with EHSG-KF, and the safety of EHSG-KF.

### 2.2 Secondary Objectives
Please refer to main protocol TBRU-dS-BA-PIIb-Study

## 3. INVESTIGATIONAL MEDICINAL PRODUCT INFORMATION SUB-STUDY

Please refer to main protocol TBRU-dS-BA-PIIb-Study

### 3.1 PRODUCTION OF IMP
Please refer to main protocol TBRU-dS-BA-PIIb-Study

### 3.2 PACKAGING, LABELLING, SUPPLY, ACCOUNTABILITY, DESTRUCTION
Please refer to main protocol TBRU-dS-BA-PIIb-Study

### 3.3 EXPERIMENTAL INTERVENTION
Please refer to main protocol TBRU-dS-BA-PIIb-Study
AND
While in the main protocol only 1 to 2 EHSG-KF grafts are foreseen, for the sub-study each patient, who is in a life- threatening situation, may receive >2 EHSG-KF grafts (graft size: 45 ± 4 cm2 or 52 ± 4cm2; graft thickness: 0.5 – 2 mm).

### 3.4 CONTROL INTERVENTION (CURRENT GOLD STANDARD)
Due to the life-threatening situation no control intervention will be performed but a maximum number of EHSG-KF grafts will be placed during the intervention.

### 3.5 RANDOMISATION
No randomisation will take place in this sub-study. After having received confirmation from the Sponsor that a patient can be admitted to the sub-study the Investigator will assign a chronological site specific patient number to the patient.

### 3.6 COMPLIANCE WITH STUDY INTERVENTION
Issues with patient compliance with the study intervention are not expected given that the graft procedure for the study product will be completed by the Investigator/surgeon. Once the patient has been discharged from hospital post-operatively, compliance issues may arise, but the main purpose of this study is to guarantee survival of the adult or adolescent patient.

### 3.7 CONCOMITANT MEDICATION
Please refer to main protocol TBRU-dS-BA-PIIb-Study

## 4. SELECTION OF STUDY POPULATION FOR THE SUB-STUDY

### 4.1 INCLUSION CRITERIA
- Patients in a life- threatening situation due to their burns and not fully eligible to participate in the TBRU-dS-BA-PIIb-Study or requiring, as per judgement of the Investigator, a higher number of EHSG-KF grafts than foreseen by the TBRU-dS-BA-PIIb-Study
- Age: ≥ 12 years of age
- Deep partial thickness and/or full-thickness burns requiring surgical wound coverage
- The following criteria must be met:
  - Expected that ≥180 cm2 of wound will remain open at 4 weeks post burn despite proceeding with treatment in accordance with the standard of care

- Signed Informed consent from the patient or the parents/ legally authorized representative

### 4.2 EXCLUSION CRITERIA

- Patients tested positive for HBV, HCV, syphilis or HIV
- Patients with known underlying or concomitant medical conditions that may interfere with normal wound healing (e.g. systemic skin and connective tissue diseases, any kind of congenital defect of metabolism including insulin-dependent diabetes mellitus, Cushing syndrome or disease, scurvy, chronic hypothyroidism, congenital or acquired immunosuppressive condition, chronic renal failure, or chronic hepatic dysfunction (Child-Pugh class B or C), severe malnutrition, or other concomitant illness which, in the opinion of the Investigator, has the potential to significantly impact wound healing )
- Pre-existing coagulation disorders as defined by INR outside its normal value, PTT >ULN and fibrinogen <LLN prior to the current hospital admission and / or at the Investigator's discretion
- Patients with known allergies to amphotericin B, gentamicin, penicillin, streptomycin, or bovine collagen

#### 4.2.1 Contraception Methods

Please refer to main protocol TBRU-dS-BA-PIIb-Study

### 4.3 WITHDRAWAL / DISCONTINUATION OF PATIENTS

Please refer to main protocol TBRU-dS-BA-PIIb-Study
AND
All efforts will be made by the Investigator to continue to obtain information about the post-surgery survival, scar quality and quality of life of the patient whenever possible.

### 4.4 DATA COLLECTION AND FOLLOW-UP FOR WITHDRAWN PATIENTS

All efforts will be made by the Investigator to continue to obtain information about the post-surgery survival, scar quality and quality of life of the patient whenever possible.

## 5. STUDY DESIGN AND COURSE OF THE SUB-STUDY

The proposed sub-study of this TBRU-dS-BA-PIIb-Study is a prospective, open label, non- randomised or controlled study.
The purpose is to evaluate the survival of the adult and adolescent patients with deep partial and full-thickness burns with a life-threatening situation and treated with EHSG-KF, and to evaluate the safety and efficacy of EHSG-KF in these patients.

### 5.1 STUDY CENTRES

Only centres participating to the TBRU-dS-BA-PIIb-Study may participate to this sub-study. This sub-study applies only to the sites a) Azienda Ospedaliera di Rilievo Nazionale Antonio Cardarelli (Italy), b) Santobono Napoli (Italy), and c) Azienda Ospedale Università Padova (Italy)

### 5.2 STUDY DESIGN

The sub-study is a prospective open – label non- randomised, non-controlled study.

### 5.3 STUDY PROCEDURES

Please refer to main protocol TBRU-dS-BA-PIIb-Study
AND
The study procedures for the sub-study will follow those of TBRU-dS-BA-PIIb-Study as much as possible, whenever feasible.

### 5.4 END OF STUDY

The end of this sub-study is defined as the last patient's last scheduled visit according to the TBRU-dS-BA-PIIb-Study which will be visit 12 (3 year follow-up visit) of the last patient entered into the study.

# 6. ENDPOINTS SUB-STUDY

## 6.1 PRIMARY ENDPOINT

The primary end point of the sub-study is the occurrence of the death of the patient.

- visit 6 (28 ± 3 days post grafting)

## 6.2 SECONDARY AND EXPLORATORY ENDPOINTS

The secondary and exploratory endpoints will be as much as possible in line with the TBRU-dS-BA-PIIb-Study, acknowledging that no comparison to a control arm will be possible due to the lack of a control arm in the sub-study. Some of the relevant endpoints are:

First secondary efficacy endpoint:

- % Epithelialization at:
  - visit 8 (90 ± 5 days post grafting)

Secondary Endpoints

Safety and efficacy evaluation, as a comparison between the EHSG-KF and control sites, based on:

- Main secondary safety endpoint:
  Clinical and microbiologic signs of infection at:
  - visit 4 (6-10 days post grafting)
  - visit 5 (21 ± 2 days post grafting)
- Main secondary efficacy endpoints:
  Scar quality at the study areas
  - Assessment of elasticity of the study areas using the Cutometer® at visit 10 (1 year ± 30 days post grafting)
  - Assessment of general scar quality at the study areas using the POSAS, a reliable and validated scar assessment tool, at visit 10 (1 year ± 30 days post grafting)
- Other secondary safety endpoint:
  Assessment and reporting of all observed adverse events will be carried out for the full duration of the study from visit 2 on.

- Other secondary efficacy endpoint:
  Epithelialization at:
  - visit 6 (28 ± 3 days post grafting)

AND

Ratio of covered surface area to biopsy site surface area at:

- visit 6 (28 ± 3 days post grafting)

## 6.5 DURATION OF PATIENT PARTICIPATION

The participation of the patients to the sub-study will be as much as possible in line with the dates and timelines of the main study protocol, unless the primary endpoint, i.e. death of the patient, is reached earlier.

# 7. STUDY ASSESSMENT AND PROCEDURES SUB-STUDY

Please refer to main protocol TBRU-dS-BA-PIIb-Study.
AND
Taking into consideration the status of the patient and based on the judgement of the Investigator, the Investigator should aim to follow as much as possible the study assessments and procedures as foreseen by the TBRU-dS-BA-PIIb-Study.

In this sub-study the number of grafts may be > 2 EHSG-KF grafts as in the main TBRU-dS-BA-PIIb-study, but will be each of the same or similar size per graft. Accordingly, more facultative biopsies might be taken.

In contrast to the TBRU-dS-BA-PIIb-Study, this sub-study does not foresee any control arm and therefore, neither any assessment nor preparation for such control arm.

# 8. ASSESSMENTS OF ENDPOINTS SUB-STUDY

## 8.1 PRIMARY ENDPOINT

Patients with extensive burns are a high risk of death. In this sub-study, severe cases who are in a life-threatening situation due to their burns as judged by the Investigator can be enrolled with the aim to prevent their death due to their extensive burn lesions. The primary endpoint of the study is the death of the patients.

## 8.2 SECONDARY AND EXPLORATORY ENDPOINT

This will be as much as possible in line with the TBRU-dS-BA-PIIb-Study taking into consideration that there might be >2 EHSG-FK grafts of and no control arm as comparison.

# 9. SAFETY SUB-STUDY

Please refer to main protocol TBRU-dS-BA-PIIb-Study.

# 10. STATISTICAL ANALYSES SUB-STUDY

Due to the nature of the sub-study it will be possible to perform only a descriptive analysis of the data.

# 11. DATA QUALITY ASSURANCE AND CONTROL SUB-STUDY

Please refer to main protocol TBRU-dS-BA-PIIb-Study.

# 12. ETHICAL AND REGULATORY ASPECTS SUB-STUDY

Please refer to main protocol TBRU-dS-BA-PIIb-Study.

# 13. PUBLICATION AND DISSEMINATION POLICY SUB-STUDY

Please refer to main protocol TBRU-dS-BA-PIIb-Study
AND
Study results of this sub-study might be published ahead of the completion of the TBRU-dS-BA-PIIb-Study.

# 14. FUNDING AND SUPPORT SUB-STUDY

Please refer to main protocol TBRU-dS-BA-PIIb-Study.

# 15. INSURANCE SUB-STUDY

Please refer to main protocol TBRU-dS-BA-PIIb-Study.

## 17. References


1. Peck MD, Jeschke MG, Duda RB. Epidemiology of burn injuries globally. *UpToDate*. 2011:1-11. https://www.uptodate.com/contents/epidemiology-of-burn-injuries-globally. Accessed October 4, 2021.
2. Bloemsma GC, Dokter J, Boxma H, Oen IMMH. Mortality and causes of death in a burn centre. *Burns*. 2008;34(8):1103-1107. doi:10.1016/j.burns.2008.02.010
3. Tompkins RG. Survival from burns in the new millennium 70 years' experience from a single institution. *Ann Surg*. 2015;261(2):263-268. doi:10.1097/SLA.0000000000000623
4. Herndon DN, Spies M. Modern burn care. *Semin Pediatr Surg*. 2001;10(1):28-31. doi:10.1053/spsu.2001.19389
5. Chua AWC, Khoo YC, Tan BK, Tan KC, Foo CL, Chong SJ. Skin tissue engineering advances in severe burns: Review and therapeutic applications. *Burn Trauma*. 2016;4(1). doi:10.1186/s41038-016-0027-y
6. Marino D, Reichmann E, Meuli M. Skingineering. *Eur J Pediatr Surg*. 2014;24(3):205-213. doi:10.1055/s-0034-1376315
7. Nyame TT, Chiang HA, Leavitt T, Ozambela M, Orgill DP. Tissue-Engineered Skin Substitutes. *Plast Reconstr Surg*. 2015;136(6):1379-1388. doi:10.1097/PRS.0000000000001748
8. Biedermann T, Boettcher-Haberzeth S, Reichmann E. Tissue engineering of skin for wound coverage. *Eur J Pediatr Surg*. 2013;23(5):375-382. doi:10.1055/s-0033-1352529
9. Böttcher-Haberzeth S, Biedermann T, Reichmann E. Tissue engineering of skin. *Burns*. 2010;36(4):450-460. doi:10.1016/j.burns.2009.08.016
10. Rowan MP, Cancio LC, Elster EA, et al. Burn wound healing and treatment: Review and advancements. *Crit Care*. 2015;19(1). doi:10.1186/s13054-015-0961-2
11. Fang T, Lineaweaver WC, Sailes FC, Kisner C, Zhang F. Clinical application of cultured epithelial autografts on acellular dermal matrices in the treatment of extended burn injuries. *Ann Plast Surg*. 2014;73(5):509-515. doi:10.1097/SAP.0b013e3182840883
12. Janžekoviĉ Z. A new concept in the early excision and immediate grafting of burns. *J Trauma - Inj Infect Crit Care*. 1970;10(12):1103-1108. doi:10.1097/00005373-197012000-00001
13. Saaiq M, Zaib S, Ahmad S. Early excision and grafting versus delayed excision and grafting of deep thermal burns up to 40% total body surface area: A comparison of outcome. *Ann Burns Fire Disasters*. 2012;25(3):143-147. https://www.ncbi.nlm.nih.gov/pmc/articles/PMC3575152/. Accessed October 4, 2021.
14. Sheridan RL, Hegarty M, Tompkins RG, Burke JF. Artificial skin in massive burns - results to ten years. *Eur J Plast Surg*. 1994;17(2):91-93. doi:10.1007/BF00176923
15. Heimbach DM, Warden GD, Luterman A, et al. Multicenter postapproval clinical trial of Integra® Dermal Regeneration Template for burn treatment. *J Burn Care Rehabil*. 2003;24(1):42-48. doi:10.1097/00004630-200301000-00009
16. Gallico GG, O'Connor NE, Compton CC, Kehinde O, Green H. Permanent Coverage of Large Burn Wounds with Autologous Cultured Human Epithelium. *N Engl J Med*. 1984;311(7):448-451. doi:10.1056/nejm198408163110706
17. O'Connor NE, Mulliken JB, Banks-Schlegel S, Kehinde O, Green H. GRAFTING OF BURNS WITH CULTURED EPITHELIUM PREPARED FROM AUTOLOGOUS EPIDERMAL CELLS. *Lancet*. 1981;317(8211):75-78. doi:10.1016/S0140-6736(81)90006-4
18. Gobet R, Raghunath M, Altermatt S, et al. Efficacy of cultured epithelial autografts in pediatric burns and reconstructive surgery. *Surgery*. 1997;121(6):654-661. doi:10.1016/S0039-6060(97)90054-4
19. Meuli M, Raghunath M. Tops and flops using cultured epithelial autografts in children. *Pediatr Surg Int*. 1997;12(7):471-477. doi:10.1007/BF01258705
20. Wood FM, Kolybaba ML, Allen P. The use of cultured epithelial autograft in the treatment of major burn injuries: A critical review of the literature. *Burns*. 2006;32(4):395-401. doi:10.1016/j.burns.2006.01.008
21. Mcheik JN, Barrault C, Levard G, Morel F, Bernard FX, Lecron JC. Epidermal healing in burns: Autologous keratinocyte transplantation as a standard procedure: Update and perspective. *Plast Reconstr Surg - Glob Open*. 2014;2(9). doi:10.1097/GOX.0000000000000176
22. Atiyeh BS, Costagliola M. Cultured epithelial autograft (CEA) in burn treatment: Three decades later. *Burns*. 2007;33(4):405-413. doi:10.1016/j.burns.2006.11.002

23. Van Zuijlen PM, Van Trier AJM, Vloemans JFPM, Groenevelt F, Kreis RW, Middelkoop E. Graft survival and effectiveness of dermal substitution in burns and reconstructive surgery in a one-stage grafting model. *Plast Reconstr Surg*. 2000;106(3):615-623. doi:10.1097/00006534-200009010-00014
24. Boyce ST, Goretsky MJ, Greenhalgh DG, Kagan RJ, Rieman MT, Warden GD. Comparative assessment of cultured skin substitutes and native skin autograft for treatment of full-thickness burns. *Ann Surg*. 1995;222(6):743-752. doi:10.1097/00000658-199512000-00008
25. Boyce ST, Simpson PS, Rieman MT, et al. Randomized, Paired-Site Comparison of Autologous Engineered Skin Substitutes and Split-Thickness Skin Graft for Closure of Extensive, Full-Thickness Burns. In: *Journal of Burn Care and Research*. Vol 38. ; 2017:61-70. doi:10.1097/BCR.0000000000000401
26. Boyce ST, Kagan RJ, Meyer NA, Yakuboff KP, Warden GD. Cultured skin substitutes combined with integra artificial skin to replace native skin autograft and allograft for the closure of excised full- thickness burns. In: *Journal of Burn Care and Rehabilitation*. Vol 20. ; 1999:453-461. doi:10.1097/00004630-199920060-00006
27. Boyce ST, Kagan RJ, Yakuboff KP, et al. Cultured skin substitutes reduce donor skin harvesting for closure of excised, full-thickness burns. *Ann Surg*. 2002;235(2):269-279. doi:10.1097/00000658-200202000-00016
28. Boyce ST, Kagan RJ, Greenhalgh DG, et al. Cultured skin substitutes reduce requirements for harvesting of skin autograft for closure of excised, full-thickness burns. *J Trauma - Inj Infect Crit Care*. 2006;60(4):821-829. doi:10.1097/01.ta.0000196802.91829.cc
29. Auger FA, López Valle CA, Guignard R, et al. Skin equivalent produced with human collagen. *Vitr Cell Dev Biol - Anim*. 1995;31(6):432-439. doi:10.1007/BF02634255
30. Michel M, L'Heureux N, Pouliot R, Xu W, Auger FA, Germain L. Characterization of a new tissue-engineered human skin equivalent with hair. *Vitr Cell Dev Biol - Anim*. 1999;35(6):318-326. doi:10.1007/s11626-999-0081-x
31. Boa O, Cloutier CB, Genest H, et al. Prospective study on the treatment of lower-extremity chronic venous and mixed ulcers using tissue-engineered skin substitute made by the self-assembly approach. *Adv Ski Wound Care*. 2013;26(9):400-409. doi:10.1097/01.ASW.0000433102.48268.2a
32. Braziulis E, Biedermann T, Hartmann-Fritsch F, et al. Skingineering I: Engineering porcine dermo-epidermal skin analogues for autologous transplantation in a large animal model. *Pediatr Surg Int*. 2011;27(3):241-247. doi:10.1007/s00383-010-2777-0
33. Schiestl C, Biedermann T, Braziulis E, et al. Skingineering II: Transplantation of large-scale laboratory-grown skin analogues in a new pig model. *Pediatr Surg Int*. 2011;27(3):249-254. doi:10.1007/s00383-010-2792-1
34. Montaño I, Schiestl C, Schneider J, et al. Formation of human capillaries in vitro: The engineering of prevascularized matrices. *Tissue Eng - Part A*. 2010;16(1):269-282. doi:10.1089/ten.tea.2008.0550
35. Pontiggia L, Biedermann T, Meuli M, et al. Markers to evaluate the quality and self-renewing potential of engineered human skin substitutes in vitro and after transplantation. *J Invest Dermatol*. 2009;129(2):480-490. doi:10.1038/jid.2008.254
36. Biedermann T, Pontiggia L, Böttcher-Haberzeth S, et al. Human eccrine sweat gland cells can reconstitute a stratified epidermis. *J Invest Dermatol*. 2010;130(8):1996-2009. doi:10.1038/jid.2010.83
37. Tharakan S, Pontiggia L, Biedermann T, et al. Transglutaminases, involucrin, and loricrin as markers of epidermal differentiation in skin substitutes derived from human sweat gland cells. *Pediatr Surg Int*. 2010;26(1):71-77. doi:10.1007/s00383-009-2517-5
38. Richard R, Miller SF, Steinlage R, Finley RK. A comparison of the tanner* and bioplasty† skin mesher systems for maximal skin graft expansion. *J Burn Care Rehabil*. 1993;14(6):690-696. doi:10.1097/00004630-199311000-00016
39. Lyons JL, Kagan RJ. The true meshing ratio of skin graft meshers. *J Burn Care Res*. 2014;35(3):257-260. doi:10.1097/BCR.0b013e3182a3692e
40. Medina A, Riegel T, Nystad D, Tredget EE. Modified Meek Micrografting Technique for Wound Coverage in Extensive Burn Injuries. *J Burn Care Res*. 2016;37(5):305-313. doi:10.1097/BCR.0000000000000244
41. Van Der Wal MBA, Vloemans JFPM, Tuinebreijer WE, et al. Outcome after burns: An observational study on burn scar maturation and predictors for severe scarring. *Wound Repair*

personalized skin

*Regen*. 2012;20(5):676-687. doi:10.1111/j.1524-475X.2012.00820.x
42. Thompson CM, Hocking AM, Honari S, Muffley LA, Ga M, Gibran NS. Genetic risk factors for hypertrophic scar development. *J Burn Care Res*. 2013;34(5):477-482. doi:10.1097/BCR.0b013e3182a2aa41
43. Sood RF, Hocking AM, Muffley LA, et al. Race and Melanocortin 1 Receptor Polymorphism R163Q Are Associated with Post-Burn Hypertrophic Scarring: A Prospective Cohort Study. *J Invest Dermatol*. 2015;135(10):2394-2401. doi:10.1038/jid.2015.197
44. Halim AS, Emami A, Salahshourifar I, Kannan TP. Keloid scarring: Understanding the genetic basis, advances, and prospects. *Arch Plast Surg*. 2012;39(3):184-189. doi:10.5999/aps.2012.39.3.184
45. Church D, Elsayed S, Reid O, Winston B, Lindsay R. Burn wound infections. *Clin Microbiol Rev*. 2006;19(2):403-434. doi:10.1128/CMR.19.2.403-434.2006
46. Bloemen MCT, Van Zuijlen PPM, Middelkoop E. Reliability of subjective wound assessment. *Burns*. 2011;37(4):566-571. doi:10.1016/j.burns.2011.02.004
47. Van Der Wal MBA, Tuinebreijer WE, Bloemen MCT, Verhaegen PDHM, Middelkoop E, Van Zuijlen PPM. Rasch analysis of the Patient and Observer Scar Assessment Scale (POSAS) in burn scars. *Qual Life Res*. 2012;21(1):13-23. doi:10.1007/s11136-011-9924-5
48. Van De Kar AL, Corion LUM, Smeulders MJC, Draaijers LJ, Van Der Horst CMAM, Van Zuijlen PPM. Reliable and feasible evaluation of linear scars by the patient and observer scar assessment scale. *Plast Reconstr Surg*. 2005;116(2):514-522. doi:10.1097/01.prs.0000172982.43599.d6
49. Mustoe TA. The Patient and Observer Scar Assessment Scale: A reliable and feasible tool for scar evaluation. Discussion. *Plast Reconstr Surg*. 2004;113(7):1966-1967. doi:10.1097/01.PRS.0000122208.16595.57
50. Öster C, Willebrand M, Dyster-Aas J, Kildal M, Ekselius L. Validation of the EQ-5D questionnaire in burn injured adults. *Burns*. 2009;35(5):723-732. doi:10.1016/j.burns.2008.11.007
51. Kildal M, Andersson G, Fugl-Meyer AR, Lannerstam K, Gerdin B. Development of a brief version of the burn specific health scale (BSHS-B). *J Trauma - Inj Infect Crit Care*. 2001;51(4):740-746. doi:10.1097/00005373-200110000-00020
52. Van Loey NE, Van De Schoot R, Gerdin B, Faber AW, Sjöberg F, Willebrand M. The burn specific health scale-brief: Measurement invariant across European countries. *J Trauma Acute Care Surg*. 2013;74(5):1321-1326. doi:10.1097/TA.0b013e31828cca84
53. Sanchez JLA, Bastida JL, Martínez MM, Moreno JMM, Chamorro JJ. Socio-economic cost and health-related quality of life of burn victims in Spain. *Burns*. 2008;34(7):975-981. doi:10.1016/j.burns.2007.12.011
54. Ridyard CH, Hughes DA. Methods for the collection of resource use data within clinical trials: A systematic review of studies funded by the UK health technology assessment program. *Value Heal*. 2010;13(8):867-872. doi:10.1111/j.1524-4733.2010.00788.x
