# Supplementary material for: Safety and efficacy of bio-engineered, autologous dermo-epidermal skin grafts in adolescent and adult burn patients: 1-year results of a prospective, randomized, controlled, multicenter phase IIB clinical trial
Source: eClinicalMedicine. 2025 Nov 28;90:103665. doi: 10.1016/j.eclinm.2025.103665 (PMC12702297; doi:10.1016/j.eclinm.2025.103665)
Supplement: Table 4 [file mmc2.docx]

**Table 1:** Inclusion and Exclusion Criteria

| **Inclusion Criteria** | **Exclusion Criteria** |
| --- | --- |
| - Age: ≥ 12 years of age - Deep partial thickness and/or full-thickness burns requiring surgical wound coverage - Expected that ≥ 90 cm2 of wound (not counting head and neck area for study patients in The Netherlands) will remain open at 4 weeks post burn despite proceeding with treatment in accordance with the standard of care. > 20% TBSA burns can be taken as guideline, but TBSA is not an inclusion criterion. - Signed Informed consent from the patient or the parents/legally authorized representative | - Patients tested positive for HBV, HCV, syphilis, or HIV - Patients with known underlying or concomitant medical conditions that may interfere with normal wound healing (e.g. systemic skin and connective tissue diseases, any kind of congenital defect of metabolism including insulin-dependent diabetes mellitus, Cushing syndrome or disease, scurvy, chronic hypothyroidism, congenital or acquired immunosuppressive condition, chronic renal failure, or chronic hepatic dysfunction (Child-Pugh class B or C), severe malnutrition, or other concomitant illness which, in the opinion of the Investigator, has the potential to significantly delay wound healing) - Severe drug and alcohol abuse - Pre-existing coagulation disorders as defined by INR outside its normal value, PTT >ULN and fibrinogen <LLN prior to the current hospital admission and / or at the Investigator’s discretion - Patients with known allergies to amphotericin B, gentamicin, penicillin, streptomycin, or bovine collagen - Previous enrolment of the patient into the current phase II study - Participation of the patient in another study with conflicting endpoints within 30 days preceding and during the present study - Patients expected not to comply with the study protocol (including patients with severe cognitive dysfunction/impairment and severe psychiatric disorders) - Suspicion of non-accidental injury - Pregnant or breast feeding females - Intention to become pregnant during the clinical course of the study (12 months) - Wounds in the head and neck area as study target area (only applicable for study patients in The Netherlands) - Enrolment of the Investigator, his/her family members, employees, and other dependent persons |
