# Supplementary material for: Safety and efficacy of bio-engineered, autologous dermo-epidermal skin grafts in adolescent and adult burn patients: 1-year results of a prospective, randomized, controlled, multicenter phase IIB clinical trial
Source: eClinicalMedicine. 2025 Nov 28;90:103665. doi: 10.1016/j.eclinm.2025.103665 (PMC12702297; doi:10.1016/j.eclinm.2025.103665)
Supplement: Supplementary Table 5 [file mmc4.docx]

**Supplementary Table 5:** Secondary and Exploratory Outcome Measures with 95% Confidence Intervals

| **Endpoint** | **mFAS** | | | **PPS** | | |
| --- | --- | --- | --- | --- | --- | --- |
|  | **N** | **Mean value** | **95% Confidence Interval** | **N** | **Mean value** | **95% Confidence Interval** |
| Ratio of Expansion Ratio’s | 15 | 7.41 | [4.71 , 10.10] | 13 | 8.25 | [5.47 , 11.04] |
| Epithelialisation 3 months | 15 | -10.4 | [-25.8 , 5.0] | 13 | -8.2 | [-24.8 , 8.5] |
| Epithelialisation 6 months | 15 | -10.1 | [-25.6 , 5.4] | 13 | -7.8 | [-24.5 , 9.0] |
| Cutometer Pliability 3 months | 12 | 0.138 | [-0.066 , 0.343] | 11 | 0.151 | [-0.073 , 0.375] |
| Cutometer Pliability 6 months | 12 | 0.047 | [-0.100 , 0.193] | 11 | 0.048 | [-0.114 , 0.211] |
| Cutometer Pliability 12 months | 12 | 0.101 | [-0.174 , 0.376] | 10 | 0.097 | [-0.245 , 0.438] |
| Cutometer Retraction 3 months | 12 | 0.116 | [-0.166 , 0.397] | 11 | 0.140 | [-0.166 , 0.447] |
| Cutometer Retraction 6 months | 12 | 0.031 | [-0.104 , 0.165] | 11 | 0.036 | [-0.113 , 0.184] |
| Cutometer Retraction 12 months | 12 | 0.075 | [-0.235 , 0.385] | 10 | 0.092 | [-0.292 , 0.477] |
| Cutometer Elasticity 3 months | 12 | 0.173 | [-0.071 , 0.417] | 11 | 0.194 | [-0.072 , 0.460] |
| Cutometer Elasticity 6 months | 12 | 0.132 | [-0.050 , 0.315] | 11 | 0.133 | [-0.069 , 0.336] |
| Cutometer Elasticity 12 months | 12 | 0.063 | [-0.094 , 0.220] | 10 | 0.049 | [-0.144 , 0.242] |
| Cutometer Viscoelasticity 3 months | 12 | 0.072 | [-0.162 , 0.307] | 11 | 0.170 | [0.064 , 0.275] |
| Cutometer Viscoelasticity 6 months | 12 | 0.010 | [-0.143 , 0.162] | 11 | 0.024 | [-0.142 , 0.189] |
| Cutometer Viscoelasticity 12 months | 12 | 0.040 | [-0.134 , 0.214] | 10 | -0.006 | [-0.205 , 0.194] |
| Cutometer Maximum Extension 3 months | 12 | 0.161 | [-0.032 , 0.353] | 11 | 0.180 | [-0.027 , 0.388] |
| Cutometer Maximum Extension 6 months | 12 | 0.103 | [-0.051 , 0.257] | 11 | 0.107 | [-0.064 , 0.278] |
| Cutometer Maximum Extension 12 months | 12 | 0.195 | [-0.131 , 0.520] | 10 | 0.203 | [-0.202 , 0.608] |
| POSAS Patient Pain 3 months | 12 | 0 | [-0.7 , 0.7] | 11 | 0.0 | [-0.8 , 0.8] |
| POSAS Patient Pain 6 months | 13 | -0.1 | [-0.2 , 0.1] | 12 | -0.1 | [-0.3 , 0.1] |
| POSAS Patient Pain 12 months | 13 | 0.2 | [-0.2 , 0.5] | 11 | 0.2 | [-0.2 , 0.6] |
| POSAS Patient Itching 3 months | 12 | -0.7 | [-2.3 , 0.9] | 11 | -0.8 | [-2.6 , 0.9] |
| POSAS Patient Itching 6 months | 13 | -0.6 | [-1.8 , 0.6] | 12 | -0.7 | [-2.0 , 0.7] |
| POSAS Patient Itching 12 months | 13 | -0.5 | [-1.8 , 0.9] | 11 | -0.5 | [-2.1 , 1.1] |
| POSAS Patient Color 3 months | 12 | 0.5 | [-1.1 , 2.1] | 11 | 0.5 | [-1.3 , 2.2] |
| POSAS Patient Color 6 months | 13 | -0.3 | [-1.8 , 1.2] | 12 | -0.2 | [-1.8 , 1.5] |
| POSAS Patient Color 12 months | 13 | 1.2 | [-0.3 , 2.8] | 11 | 1.3 | [-0.4 , 2.9] |
| POSAS Patient Stiffness 3 months | 11 | -0.6 | [-2.4 , 1.1] | 10 | -1.0 | [-2.7 , 0.7] |
| POSAS Patient Stiffness 6 months | 13 | -1.8 | [-3.3 , -0.3] | 12 | -1.7 | [-3.3 , -0.1] |
| POSAS Patient Stiffness 12 months | 12 | -1.2 | [-2.3 , 0.0] | 10 | -1.5 | [-2.8 , -0.2] |
| POSAS Patient Thickness 3 months | 10 | -1.2 | [-3.2 , 0.8] | 9 | -0.7 | [-2.5 , 1.2] |
| POSAS Patient Thickness 6 months | 13 | -1.0 | [-2.2 , 0.2] | 12 | -0.8 | [-2.1 , 0.5] |
| POSAS Patient Thickness 12 months | 12 | -0.7 | [-2.6 , 1.3] | 10 | -0.8 | [-3.2 , 1.6] |
| POSAS Patient Regularity 3 months | 11 | -2.4 | [-4.8 , 0.0] | 10 | -1.7 | [-3.8 , 0.4] |
| POSAS Patient Regularity 6 months | 13 | -2.0 | [-3.8 , -0.2] | 12 | -1.9 | [-3.9 , 0.0] |
| POSAS Patient Regularity 12 months | 12 | -0.5 | [-2.4 , 1.4] | 10 | -0.5 | [-2.9 , 1.9] |
| POSAS Patient Overall Opinion 3 months | 12 | -1.9 | [-3.5 , -0.3] | 11 | -1.5 | [-2.8 , -0.1] |
| POSAS Patient Overall Opinion 6 months | 13 | -1.0 | [-2.6 , 0.6] | 12 | -0.8 | [-2.4 , 0.9] |
| POSAS Patient Overall Opinion 12 months | 12 | -0.1 | [-2.1 , 2.0] | 10 | -0.5 | [-2.8 , 1.8] |
| POSAS Observer Vascularity 3 months | 12 | 0.8 | [-0.3 , 1.9] | 11 | 0.8 | [-0.4 , 2.1] |
| POSAS Observer Vascularity 6 months | 13 | 0.3 | [-0.7 , 1.3] | 12 | 0.4 | [-0.6 , 1.4] |
| POSAS Observer Vascularity 12 months | 13 | 0.5 | [-0.5 , 1.5] | 11 | 0.5 | [-0.7 , 1.8] |
| POSAS Observer Pigmentation 3 months | 12 | -0.3 | [-1.8 , 1.3] | 11 | -0.1 | [-1.7 , 1.6] |
| POSAS Observer Pigmentation 6 months | 13 | 0.1 | [-1.4 , 1.6] | 12 | 0.0 | [-1.6 , 1.6] |
| POSAS Observer Pigmentation 12 months | 13 | 0.9 | [-0.8 , 2.6] | 11 | 1.1 | [-0.9 , 3.1] |
| POSAS Observer Thickness 3 months | 12 | -0.6 | [-2.1 , 0.9] | 11 | -0.5 | [-2.1 , 1.2] |
| POSAS Observer Thickness 6 months | 13 | -0.5 | [-1.6 , 0.5] | 12 | -0.6 | [-1.7 , 0.5] |
| POSAS Observer Thickness 12 months | 13 | -0.2 | [-1.3 , 0.9] | 11 | -0.5 | [-1.4 , 0.5] |
| POSAS Observer Relief 3 months | 12 | -1.5 | [-3.3 , 0.3] | 11 | -1.4 | [-3.3 , 0.6] |
| POSAS Observer Relief 6 months | 13 | -1.9 | [-3.0 , -0.8] | 12 | -2.0 | [-3.2 , -0.8] |
| POSAS Observer Relief 12 months | 13 | -2.8 | [-3.8 , -1.7] | 11 | -2.7 | [-3.9 , -1.5] |
| POSAS Observer Pliability 3 months | 12 | -0.4 | [-1.5 , 0.6] | 11 | -0.4 | [-1.5 , 0.8] |
| POSAS Observer Pliability 6 months | 13 | -1.4 | [-2.5 , -0.3] | 12 | -1.3 | [-2.6 , -0.1] |
| POSAS Observer Pliability 12 months | 13 | -0.5 | [-1.1 , 0.2] | 11 | -0.5 | [-1.3 , 0.2] |
| POSAS Observer Surface Area 3 months | 11 | -0.7 | [-1.9 , 0.4] | 10 | -0.6 | [-1.8 , 0.6] |
| POSAS Observer Surface Area 6 months | 13 | -0.5 | [-1.7 , 0.6] | 12 | -0.3 | [-1.5 , 0.8] |
| POSAS Observer Surface Area 12 months | 13 | -0.1 | [-0.4 , 0.2] | 11 | -0.1 | [-0.5 , 0.3] |
| POSAS Observer Overall Opinion 3 months | 12 | -0.9 | [-1.7 , -0.1] | 11 | -0.8 | [-1.7 , 0.1] |
| POSAS Observer Overall Opinion 6 months | 13 | -1.0 | [-2.0 , 0.0] | 12 | -1.0 | [-2.1 , 0.1] |
| POSAS Observer Overall Opinion 12 months | 13 | -0.8 | [-1.5 , -0.0] | 11 | -0.7 | [-1.6 , 0.2] |
| POSAS Observer Total Score 3 months | 12 | -3.5 | [-9.7 , 2.7] | 11 | -2.8 | [-9.5 , 3.8] |
| POSAS Observer Total Score 6 months | 13 | -5.0 | [-10.8 , 0.8] | 12 | -4.8 | [-11.2 , 1.5] |
| POSAS Observer Total Score 12 months | 13 | -2.9 | [-7.3 , 1.4] | 11 | -2.9 | [-8.1 , 2.3] |
| Graft Take at Day 6-10 | 15 | 0.78 | [0.58 , 0.98] | / | / | / |
| Colormeter Erythem 3 months | 12 | -0.256 | [-2.152 , 1.641] | / | / | / |
| Colormeter Erythem 6 months | 13 | -0.558 | [-2.334 , 1.229] | / | / | / |
| Colormeter Erythem 12 months | 13 | 0.683 | [-1.048 , 2.414] | / | / | / |
| Colormeter Melanin 3 months | 12 | -0.653 | [-3.862 , 2.557] | / | / | / |
| Colormeter Melanin 6 months | 13 | 0.051 | [-2.926 , 3.027] | / | / | / |
| Colormeter Melanin 12 months | 13 | -1.310 | [-4.096 , 1.476] | / | / | / |
